# How many genera and species of Galerucinae *s. str.* do we know? Updated statistics (Coleoptera, Chrysomelidae)

**DOI:** 10.3897/zookeys.720.13517

**Published:** 2017-12-11

**Authors:** Rui-E Nie, Jan Bezděk, Xing-Ke Yang

**Affiliations:** 1 Key Laboratory of Zoological Systematics and Evolution, Institute of Zoology, Chinese Academy of Sciences, Beijing, 100101, China; 2 Mendel University, Department of Zoology, Zemědělská, 1, 613 00 Brno, Czech Republic

**Keywords:** Biodiversity, checklist, Chrysomeloidea, leaf beetles, worldwide

## Abstract

Galerucinae *s. str.* is a rich group of leaf beetles. A new, up-to date checklist of Galerucinae genera in the world is provided, including the number of valid species of each genus. Genera and species were counted in literature published before the end of 2016. In summary, 7145 species (7132 recent, 13 fossils) and 192 subspecies from 543 genera (542 recent, 1 fossil) were quantified in Galerucinae *s. str.* In comparison with the previous catalogue of worldwide Galerucinae (Wilcox 1971–1973), an additional 91 valid genera, 1341 valid species (1337 recent, 4 fossils) and 38 subspecies have been published; 43 genera were synonymized, four genera were transferred into Alticini, two subgenera were elevated to genus rank, and one genus was downgraded to subgenus rank. The updated list of references to taxonomic publications on Galerucinae *s. str.* from the period 1971–2016 is provided.

## Introduction

Galerucinae *sensu stricto* (i.e., not including Alticini) belongs to Chrysomelidae (Coleoptera) and is one of the largest groups of leaf beetles (Yang et al. 2015). Adult Galerucinae can be identified by an oval to oblong body, with the head visible from above and inserted into the prothorax. The front coxal cavity is either open or closed. Tarsi are pseudotetramerous with the third segment bifid, and the fourth segment very small in size, and nested in the third one. The hind femur is slender without a femoral spring. The antenna has eleven segments; the antennal insertions are situated close together in front or between the eyes. Frontal tubercles are usually present and well developed. The elytral sensilla patch is usually single (Samuelson 1996; Nadein and Bezděk 2014).

Galerucinae *s. str.* is a key group to study the phylogeny of Polyphaga. The adults and larvae of Galerucinae *s. str.* are herbivorous, and most of them show host specificity. The special relationship of Galerucinae *s. str.* and its host plants makes the group a good model to study the evolution of herbivorous beetles, the convergent evolution of insects and plants (Futuyma and McCafferty 1990), and the evolutionary mechanisms of biodiversity (Farrell et al. 1992; Mitter and Farrell 1991). In addition, many species are used for biological control of weeds or are important pest species of agriculture (Vencl and Morton 1998; Xue et al. 2007; Bunnige et al. 2008; Xue and Yang 2008; Nie et al. 2012).

Among Chrysomelidae, the closest relative to Galerucinae *s. str.* is Alticinae (or flea beetles) discussed further below. Both groups have very rich diversity. Nadein and Bezděk (2014) estimated 6500 species in ca. 600 genera within Galerucinae *s. str.*; and about 8000 species in 534 genera within Alticinae *s. str.* The two groups have very similar morphological characters. The key morphological character used to distinguish both groups is the metafemoral extensor tendon (MET) in the hind femora (also known as metafemoral spring, metafemoral apodeme, or Maulik’s organ), which is a structure that permits large jumps for predator evasion (Furth and Suzuki 1990; Furth and Suzuki 1998; Nadein and Betz 2016). The presence of MET was not always mentioned in the descriptions of genera or species. Actually some species with slender hind femora have MET, such as *Mandarella* Duvivier, 1892. In contrast, some species with swell hind femora are without MET such as *Orthaltica* Crotch, 1873 (Furth and Suzuki 1994; Konstantinov and Prathapan 2008). Some genera are called “problematic genera” with the presence or absence of a MET and not fitting other characters. Recently, some researchers found that the MET may have multiple origins, evolving at least two or three times (Ge et al. 2011; 2012; Nie et al. 2017).

**Figure 1.**
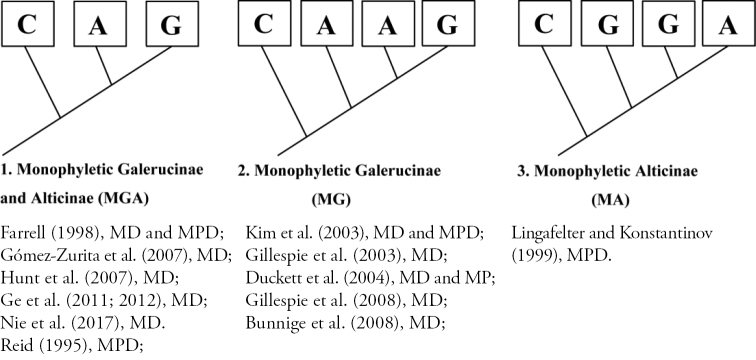
The three hypotheses of the phylogeny of Galerucinae and Alticinae. The supporters of each hypothesis are listed below each. Note: MD = Molecular Data; MPD = Morphological Data.

The phylogenetic relationship of Galerucinae *s. str.* and flea beetles has been disputed for many decades and is still unclear and controversial. Some recent investigations support the inclusion of the traditional alticines in Galerucinae, yet classification remains a challenge as neither the traditional “Galerucinae” nor the traditional “Alticinae” are monophyletic (Bouchard et al. 2011; Nadein and Bezděk 2014; Reid 2014). Other studies suggest considering both groups as subfamilies (e.g. Löbl and Smetana 2010, Ge et al. 2011, 2012; Haddad and McKenna 2016). So far, three hypotheses of evolutionary relationships have been proposed based on morphological or molecular data (Fig. 1). Among these tree hypotheses, a sister group relationship of Galerucinae and Alticinae was supported by the most molecular or morphological analyses (Seeno and Wilcox 1982; Doguet 1994; Farrell 1998; Gómez-Zurita et al. 2007; Hunt et al. 2007; Bouchard et al. 2011; Ge et al. 2011, 2012; Nie et al. 2017). Some of the recently established groupings, based on DNA sequences, still need further in-depth analysis because they are phylogenetically and biogeographically incomplete (Biondi and D´Alessandro 2012). In this study there is no attempt to resolve the relationship of both groups. The reason Galerucinae and Alticinae are treated as two equal groups is strictly technical in order to count the genera and the species correctly.

Some important catalogues of Galerucinae *s. str.* have been published during the 20^th^ century. Weise (1924) catalogued 3678 species from 305 genera. The last comprehensive Galerucinae catalogue published by Wilcox (1971–1973) included 5802 species (including fossil taxa) in 476 genera. The summarized generic arrangement (495 genera) was presented by Seeno and Wilcox (1982). However, the taxonomy of Galerucinae *s. str.* has not been summarized during the last 40 years. Many new species, new genera, new names, or new synonymies have been proposed. This work seeks to provide a new detailed, up-to date, summary of global Galerucinae *s. str.* taxonomy.

## Methods

All the currently valid genera names (in nomenclatorial sense, both recent and fossil) of subfamily Galerucinae *s. str.* in the world published before December 31, 2016 are listed. The references are mainly based on the database Zoological records and Jan Bezděk´s personal catalogue. Each genus includes the present number of recent species, subspecies and fossil species, generic distribution, list of subgenera and generic synonyms, and references to publications which influenced the number of genera and species from (Wilcox´s 1971–1973) catalogues to present (including important redescriptions). The references omitted in (Wilcox´s 1971–1973) catalogue are included.

For each genus, an outline of its present geographic distribution (based on Löbl and Smetana 2010) is provided. The abbreviation of fauna is as following:

**AFR** Afrotropical Region;

**AUR** Australian Region;

**NAR** Nearctic Region;

**NTR** Neotropical Region;

**ORR** Oriental Region;

**PAR** Palaearctic Region.

For genera with restricted distributions, the countries are listed. For the genera (e.g. *Pyrrhalta*, *Xanthogaleruca*, *Tricholochmaea*, *Galerucella*, *Galeruca*) with controvertible classified rank, we follow the Palaearctic catalogue (Beenen 2010). The authorship of the genera published in Dejean (1836) follows the paper by Bousquet and Bouchard (2013).

Wilcox published his catalogue in four fascicles. The fascicles 1–3 (Wilcox 1971–1973) comprise the catalogue itself including precisely documented species and genera. The last fascicle (Wilcox 1975) included addenda, index, and references to the papers published in several previous years. For comparison of genera and species, we used only fascicles 1–3. The fourth fascicle will be included in subsequent publications.

## Results

Wilcox (1971–1973) published 5802 species (5793 recent species + 9 fossil ones) and 154 subspecies in 476 genera. As of the end of December 2016, Galerucinae *s. str.* contains 7145 species (7132 recent, 13 fossils) and 192 subspecies from 543 genera (542 recent, one fossil). Among these 543 genera, 91 novel valid genera (including one fossil) have been published since 1974. Since 1974, 1341 valid species (including four fossils) and 38 subspecies have been added. A total of 194 genera is listed in synonymy, of which 145 were listed as synonyms in Wilcox (1971–1973). After 1973, 43 genera were synonymized, four genera were transferred into Alticini, two subgenera were elevated to genus rank, and one genus was downgraded to subgenus. The detailed statistics on the number of genera, species and subspecies, geographic distribution, as well as the subgenera, the generic synonyms and references can be seen in Supplementary information/data 1.

Since 1974, some genera have been increased by many species e.g. *Monolepta* (113 species), *Paleosepharia* (63 species), *Apophylia* (47 species), *Paridea* (41 species), *Pyrrhalta* (38 species), while 292 genera have not increased. Surprisingly, the species number decreased from 259 to 246 in African Monoleptites, a group deeply revised in last twenty years, because of many new synonyms. Similar decrease in species number is expected in other species-rich genera with color variability, e.g., *Diacantha* Chevrolat, 1836 (see Wagner 2017). The major contribution of new generic descriptions since (Wilcox´s 1971–1973) catalogue has been made by Medvedev (22 genera), Wagner (17 genera), Chen (eleven genera), Kimoto (seven genera), Mohamedsaid (seven genera), Silfverberg (six genera), Bezděk (five genera), Beenen (five genera), Clark (four genera), Shute (three genera), and Lopatin (two genera).

**Figure 2.**
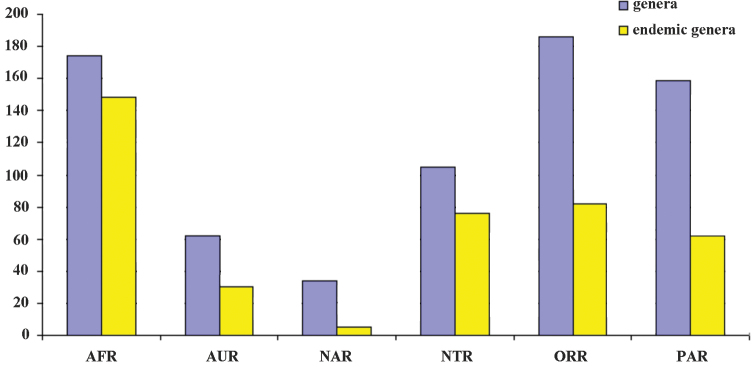
The numbers of genera and endemic genera in geographical regions.

The distribution of Galerucinae *s. str.* is worldwide. Altogether 186 genera (34.3%) are distributed in the Oriental Region, followed by Afrotropical Region (174 genera, 32.0%), Palaearctic Region (159 genera, 29.1%), Neotropical Region (105 genera, 19.3%), Australian Region (62 genera, 11.4%), and Nearctic Region (34 genera, 6.3%). A very high level of generic endemism is exhibited for the Afrotropical Region where 148 genera from total 174 are endemic (31 genera are endemic for Madagascar) and for Neotropical Region with 76 endemic genera from total 105. In the Afrotropical Region high level of generic endemism in Galerucinae *s. str.* (85%) corresponds to Alticinae with 71% (Biondi and D´Alessandro 2010). In all other regions the level of generic endemism of Galerucinae *s. str.* is below 50%. For the total numbers of genera and endemic genera in all the regions see Fig. 2.

There are no cosmopolitan genera in Galerucinae *s. str.* The most diverse and most widely distributed genus is *Monolepta* with more than 700 species occurring in almost all the regions but missing in the Nearctic Region (Riley et al. 2003). Additional species-rich genera with wide distribution like *Luperus* Geoffroy, 1762 (97 species), *Luperodes* Motschulsky, 1858 (77 species), and *Calomicrus* Dillwyn, 1829 (85 species) are evidently polyphyletic and the future revisions will lead to the geographical restriction of these genera.

**Figures 3–8.**
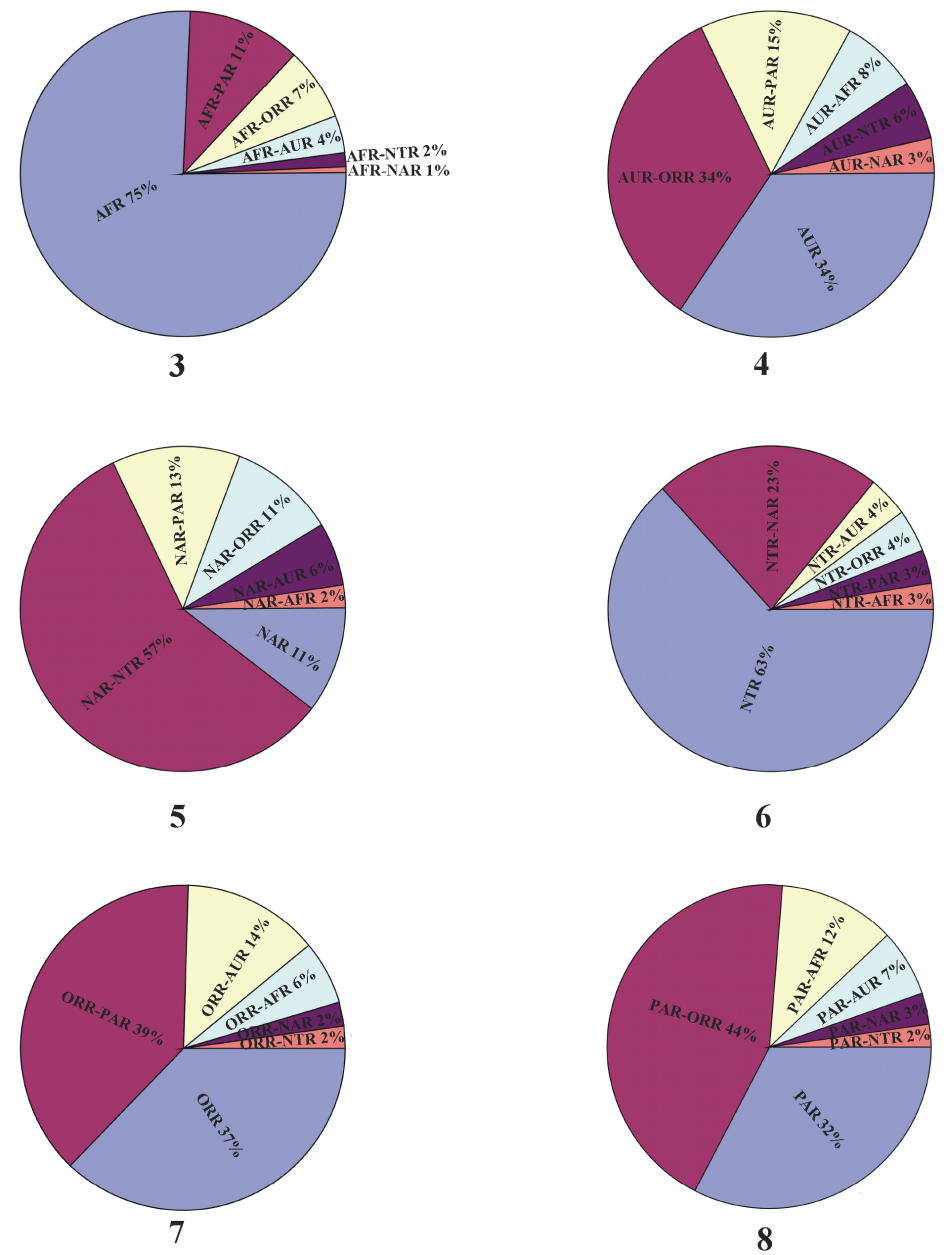
Distribution of genera of Galerucinae *s. str.* in the different zoogeographical regions showing generic endemism percentage and percentage of the genera shared with other regions. **3** Afrotropical Region (AFR) **4** Australian Region (AUR) **5** Nearctic Region (NAR) **6** Neotropical Region (NTR) **7** Oriental Region (ORR) **8** Palaearctic Region (PAR).

The distribution of many genera is shared with adjacent regions. For example, 37% of Oriental genera are endemic while 39% are shared with Palaearctic Region and additional 14% with Australian fauna. As expected only a low percentage (2%) of genera occurs in Nearctic/Neotropical regions and some another region (ORR-AFR 6%). On the other hand, 27 genera are shared with both Nearctic and Neotropical regions. It is necessary to mention that distribution of some genera is often only marginal in adjacent region (for example in the eastern border of Palaearctic and Oriental Regions). The generic endemism percentage in comparison with the percentage of the genera shared with other regions is graphed in Figs 3–8.

Among 92 genera proposed after Wilcox´s (1971–1973) catalogue, the fauna of the Oriental region has increased by 36 genera (39.1%), followed by Palaearctic Region (24 genera, 26.1%), Afrotropical Region (22 genera, 23.9%), Neotropical Region (eight genera, 8.7%), Australian Region (six genera, 6.5%), and Nearctic Region (one genus, 1.1%).

The following taxa listed by Wilcox (1971–1973) or described later in Galerucinae *s. str.* have been transferred to other Chrysomelidae groups:

– *Hildebrandtina* Weise, 1910 belongs to Alticinae *s. str.* (see Biondi and D´Alessandro 2010, 2012).

– *Borbaita* Bechyné, 1958, *Micrantipha* Blackburn, 1896, *Neoclitena* Abdullah & Qureshi, 1968, *Philocalis* Dejean, 1836, and *Scelidopsis* Jacoby, 1888 were transferred to Alticinae *s. str.* by Wilcox (1975) and their position is widely accepted (see Seeno and Wilcox 1982; Döberl 2010).

– *Lochmaeina* Medvedev, 1956 and *L. rosea* Medvedev, 1956 are synonyms of *Sangariola* and *S. punctatostriata* Motschulsky (Alticinae *s. str.*) (see Wilcox 1975).

– *Stenoluperus* Ogloblin, 1936 was synonymized with *Mandarella* Duvivier, 1892 which belongs to Alticinae *s. str.* (see e.g., Wilcox 1975; Medvedev 2012; Lee et al. 2016). However, its position is still questionable (Nie et al. 2017).

– *Luperodes antillarum* Blake, 1937 was synonymized with *Lysathia ludoviciana* Fall, 1910 which belongs to Alticinae *s. str.* (see Wilcox 1975, Virkki 1979).

– *Luperus uenoi* Kimoto, 1969 was transferred to *Mandarella* Duvivier, 1892 which belongs to Alticinae *s. str.* (see Lee et al. 2016).

– *Chaloenus* Westwood, 1862 belongs to Alticinae *s. str.* (see Konstantinov and Prathapan 2008).

– *Calomicrus sibiricus* (Csiki, 1916) was transferred to *Luperomorpha* Weise, 1887 which belongs to Alticinae *s. str.* by Bezděk (2007).

– *Oides neobengalensis* Rizvi & Kamaluddin, 2011 is synonym of *Clytra subfasciata* Lacordaire, 1848 which belongs to Clytrini of Cryptocephalinae (see Bezděk 2012, 2016).

– *Paramerista* Lopatin, 2011 is synonym of *Podontia* Dalman, 1824 and *Paramerista luteola* Lopatin, 2011 is synonym of *Podontia lutea* (Olivier, 1790) which belongs to Alticinae *s. str.* (see Bezděk 2012).

## Supplementary materials

Supplementary material 1 Genera list and related files Data type: species data Explanation note: The list of all valid genera (both recent and fossil) of subfamily Galerucinae s. str. in the world. This is an open access article distributed under the terms of the Creative Commons Attribution License (CC BY 4.0), which permits unrestricted use, distribution, and reproduction in any medium, provided the original author and source are credited.Rui-E Nie, Jan Bezděk, Xing-Ke Yang

## References

[B1] Beenen R (2010) Chrysomelidae: Galerucinae. In: Löbl I Smetana A (Eds) Catalogue of Palaearctic Coleoptera, Volume 6. Chrysomeloidea. Apollo books, Stenstrup, 443–491.

[B2] Bezděk J (2007) Taxonomical changes in Palaearctic Luperini (Coleoptera: Chrysomelidae: Galerucinae). Annales Zoologici 57: 257–266.

[B3] Bezděk J (2012) Taxonomic and faunistic notes on Oriental and Palaearctic Galerucinae and Cryptocephalinae (Coleoptera: Chrysomelidae). Genus 23: 375–418. http://www.biol.uni.wroc.pl/cassidae/Bezdek_Taxonomic%20notes%20on%20Chrysomelidae_low.pdf

[B4] Bezděk J (2016) Revision of the Clytra subfasciata species group (Coleoptera: Chrysomelidae: Cryptocephalinae: Clytrini). Zoology in the Middle East 62: 148–157. http://dx.doi.org/10.1080/09397140.2016.1182772

[B5] Biondi M D´Alessandro P (2010) Genus-group names of Afrotropical flea beetles (Coleoptera: Chrysomelidae: Alticinae): Annotated catalogue and biogeographical notes. European Journal of Entomology 107: 401–424. https://doi.org/10.14411/eje.2010.049

[B6] Biondi M D´Alessandro P (2012) Afrotropical flea beetle genera: a key to their identification, updated catalogue and biogeographical analysis (Coleoptera, Chrysomelidae, Galerucinae, Alticini). ZooKeys 253: 1–58. https://doi.org/10.3897/zookeys.253.3414 10.3897/zookeys.253.3414 PMC3560840 23378812

[B7] Bouchard P Bousquet Y Davies AE Alonso-Zarazaga MA Lawrence JF Lyal CHC Newton AF Reid CAM Schmitt M Ślipiński SA (2011) Family-group names in Coleoptera (Insecta). ZooKeys 88: 1–972. https://doi.org/10.3897/zookeys.88.807 10.3897/zookeys.88.807 PMC3088472 21594053

[B8] Bousquet Y Bouchard P (2013) The genera in the second catalogue (1833–1836) of Dejean’s Coleoptera collection. ZooKeys 282: 1–219. https://doi.org/10.3897/zookeys.282.4401 10.3897/zookeys.282.4401 PMC3677338 23794836

[B9] Bunnige M Hilker M Dobler S (2008) Convergent evolution of chemical defence in galerucine larvae. Biological Journal of the Linnean Society 93: 165–175. https://doi.org/10.1111/j.1095-8312.2007.00912.x

[B10] Dejean PFAM (1836) Catalogue des coléoptères de la collection de M. le Comte Dejean. Deuxième édition. Méquignon-Marvis Père et Fils, Paris, 442 pp.

[B11] Döberl M (2010) Chrysomelidae: Alticinae. In: Löbl I Smetana A (Eds) Catalogue of Palaearctic Coleoptera, Volume 6. Chrysomeloidea. Apollo books, Stenstrup, 491–563.

[B12] Doguet S (1994) Coléoptères Chrysomelidae. Volume 2. Alticinae. Faune de France 80. Fédération Française des Sociétés de Science Naturelles, Paris, 694 pp.

[B13] Duckett CN Gillespie JJ Kjer KM (2004) Relationships among the subfamilies of Chrysomelidae inferred from small subunit ribosomal DNA and morphology, with special emphasis on the relationship among the flea beetles and the Galerucinae. In: Jolivet P Santiago-Blay JA Schmitt M (Eds) New developments in the biology of Chrysomelidae. SPB Academic Publishing, The Hague, 3–18.

[B14] Farrell BD (1998) “Inordinate fondness” explained: Why are there so many beetles? Science 281: 555–559. https://doi.org/10.1126/science.281.5376.555 10.1126/science.281.5376.555 9677197

[B15] Farrell BD Mitter C Futuyma D (1992) Diversification at the insect-plant interface. BioScience 42: 34–42. https://doi.org/10.2307/1311626

[B16] Furth DG Suzuki K (1990) The metatibial extensor and flexor tendons in Coleoptera. Systematic Entomology 15: 443–448. https://doi.org/10.1111/j.1365-3113.1990.tb00076.x

[B17] Furth DG Suzuki K (1994) Character correlation studies of problematic genera of Alticinae in relation to Galerucinae (Coleoptera: Chrysomelidae). Proceedings of the third international symposium on the Chrysomelidae, Beijing, 116–135.

[B18] Furth DG Suzuki K (1998) Studies of Oriental and Australian Alticinae genera based on the comparative morphology of the metafemoral spring, genitalia, and hind wing venation. Proceedings of the Fourth International Symposium on the Chrysomelidae Proceedings of a symposium 20^th^ International Congress of Entomology, Museo Regionale di Scienze Naturali, 1–327.

[B19] Futuyma DJ McCafferty SS (1990) Phylogeny and the evolution of host plant associations in the leaf beetle genus *Ophraella* (Coleoptera, Chrysomelidae). Evolution 44: 1885–1913. https://doi.org/10.2307/2409602 28564433 10.1111/j.1558-5646.1990.tb04298.x

[B20] Ge DY Chesters D Gómez-Zurita J Zhang LJ Yang XK Vogler AP (2011) Anti-predator defence drives parallel morphological evolution in flea beetles. Proceedings of the Royal Society B: Biological Sciences 2011: 2133–2141. https://doi.org/10.1098/rspb.2010.1500 10.1098/rspb.2010.1500 PMC3107618 21159678

[B21] Ge DY Gómez-Zurita J Chesters D Yang XK Vogler AP (2012) Suprageneric systematics of flea beetles (Chrysomelidae: Alticinae) inferred from multilocus sequence data. Molecular Phylogenetics and Evolution 62: 793–805. https://doi.org/10.1016/j.ympev.2011.11.028 22197803 10.1016/j.ympev.2011.11.028

[B22] Gillespie JJ Kjer KM Duckett CN Tallamy DW (2003) Convergent evolution of cucurbitacin feeding in spatially isolated rootworm taxa (Coleoptera: Chrysomelidae; Galerucinae, Luperini). Molecular Phylogenetics and Evolution 29: 161–175. https://doi.org/10.1016/S1055-7903(03)00256-2 12967617 10.1016/s1055-7903(03)00256-2

[B23] Gillespie JJ Tallamy DW Riley EG Cognato AI (2008) Molecular phylogeny of rootworms and related galerucine beetles (Coleoptera: Chrysomelidae). Zoologica Scripta 37: 195–222. https://doi.org/10.1111/j.1463-6409.2007.00320.x

[B24] Gómez-Zurita J Hunt T Kopliku F Vogler AP (2007) Recalibrated tree of leaf beetles (Chrysomelidae) indicates independent diversification of angiosperms and their insect herbivores. PLoS ONE 2: e360. https://doi.org/10.1371/journal.pone.0000360 10.1371/journal.pone.0000360 PMC1832224 17426809

[B25] Haddad S McKenna DD (2016) Phylogeny and evolution of the superfamily Chrysomeloidea (Coleoptera: Cucujiformia). Systematic Entomology 41: 697–716. https://doi.org/10.1111/syen.12179

[B26] Hunt T Bergsten J Levkanicova Z Papadopoulou A John OS Wild R Hammond PM Ahrens D Balke M Caterino MS Gómez-Zurita J Ribera I Barraclough TG Bocakova M Bocak L Vogler AP (2007) A comprehensive phylogeny of beetles reveals the evolutionary origins of a superradiation. Science 318: 1913–1916. https://doi.org/10.1126/science.1146954 18096805 10.1126/science.1146954

[B27] Kim SJ Kjer KM Duckett CN (2003) Comparison between molecular and morphological-based phylogenies of galerucine/alticine leaf beetles (Coleoptera: Chrysomelidae). Insect Systematics & Evolution 34: 53–64. https://doi.org/10.1163/187631203788964890

[B28] Konstantinov AS Prathapan KD (2008) New generic synonyms in the Oriental flea beetles (Coleoptera: Chrysomelidae). Coleopterists Bulletin 62: 381–418. https://doi.org/10.1649/1089.1

[B29] Lee CF Tsai CL Konstantinov A Yeh WB (2016) Revision of *Mandarella* Duvivier from Taiwan, with a new species, new synonymies and identities of highly variable species (Insecta, Chrysomelidae, Galerucinae, Alticini). Zookeys 568: 23–49. https://doi.org/10.3897/zookeys.568.7125 10.3897/zookeys.568.7125 PMC4829668 27103872

[B30] Lingafelter S Konstantinov AS (1999) The monophyly and relative rank of alticine and galerucine leaf beetles: A cladistic analysis using adult morphological characters. Entomologica Scandinavica 30: 397–416. https://doi.org/10.1163/187631200X00525

[B31] Löbl I Smetana A (2010) Catalogue of Palaearctic Coleoptera, Volume 6, Chrysomeloidea. Apollo books, Stenstrup, 924 pp.

[B32] Medvedev LN (2012) To the knowledge of the genera *Mandarella* Duvivier and *Stenoluperus* Ogloblin (Insecta: Chrysomelidae: Alticinae) from the Himalayas. In: Hartmann M Weipert J (Eds) Biodiversität und Naturausstattung im Himalaya IV. Verein der Freunde und Förderer des Naturkundemuseums Erfurt e.V., Erfurt, 423–427.

[B33] Mitter C Farrell B (1991) Macroevolutionary aspects of insect-plant relationships. Insect-plant Interactions 3: 35–78.

[B34] Nadein K Bezděk J (2014) Galerucinae Latreille, 1802. In: Leschen RAB Beutel RG (Eds) Handbook of Zoology. Coleoptera, beetles. Morphology and systematics. Volume 3. Walter de Gruyter, Berlin/Boston, 251–259.

[B35] Nadein K Betz O (2016) Jumping mechanisms and performance in beetles. I. Flea beetles (Coleoptera: Chrysomelidae: Alticini). Journal of Experimental Biology 219: 2015–2027. https://doi.org/10.1242/jeb.140533 27385755 10.1242/jeb.140533

[B36] Nie RE Xue HJ Hua Y Yang XK Vogler AP (2012) Distinct species or colour polymorphism? Life history, morphology and sequence data separate two species of elm leaf beetles (Coleoptera: Chrysomelidae). Systematics and Biodiversity 10: 133–146. https://doi.org/10.1080/14772000.2012.687783

[B37] Nie RE Breeschoten T Timmermans MJTN Nadein K Xue HJ Bai M Huang Y Yang XK Vogler AP (2017) The phylogeny of Galerucinae (Coleoptera: Chrysomelidae) and the performance of mitochondrial genomes in phylogenetic inference compared to nuclear rRNA genes. Cladistics 33: 1–18. https://doi.org/10.1111/cla.12196 10.1111/cla.12196 34645082

[B38] Reid CAM (1995) A cladistic analysis of subfamilial relationships in the Chrysomelidae sensu lato (Chrysomeloidea). In: Pakaluk J Slipinski SA (Eds) Biology, phylogeny and classification of Coleoptera: papers celebrating the 80th birthday of Roy A. Crowson. Muzeum i Instytut Zoologii PAN, Warsaw, 559–631.

[B39] Reid CAM (2014) Chrysomeloidea. In: Leschen RAB Beutel RG (Eds) Handbook of Zoology. Coleoptera, beetles. Morphology and systematics. Volume 3. Walter de Gruyter, Berlin/Boston, 11–15.

[B40] Riley EG Clark SM Seeno TN (2003) Catalog of leaf beetles of America north of Mexico. The Coleopterists Society, Sacramento, 290 pp.

[B41] Samuelson GE (1996) Binding sites: elytron-to-body meshing structures of possible significance in the higher classification of Chrysomeloidea. In: Jolivet PHA Cox ML (Eds) Chrysomelidae Biology. Volume 1: The classification, phylogeny and genetics. SPB Academic Publishing, Amsterdam, 267–290.

[B42] Seeno TN Wilcox JA (1982) Leaf beetle genera (Coleoptera: Chrysomelidae). Entomography 1: 1–222.

[B43] Vencl FV Morton TC (1998) The shield defense of the sumac flea beetle, *Blepharida rhois* (Chrysomelidae: Alticinae). Chemoecology 8: 25–32. https://doi.org/10.1007/PL00001800

[B44] Virkki N (1979) Brief notes on the cytology of Neotropical Coleoptera. III. “*Luperodes antillarum* Blake” = *Lysathia ludoviciana* (Fall). Journal of Agriculture of the University of Puerto Rico 63: 100–101.

[B45] Wagner T (2017) Quo vadis biodiversity? Species richness following twenty years of taxonomic revisions on Afrotropical Galerucinae *s. str.* (Coleoptera, Chrysomelidae). In: Schmitt M Chaboo CS (Eds) Research on Chrysomelidae 7. ZooKeys 720: 131–137. https://doi.org/10.3897/zookeys.720.14011 10.3897/zookeys.720.14011 PMC5740478 29290730

[B46] Weise J (1924) Chrysomelidae: 13. Galerucinae. In: Junk W, Schenkling S (Eds) Coleopterorum Catalogus, Pars 78. W. Junk, Berlin, 225 pp.

[B47] Wilcox JA (1971) Coleopterum Catalogus Supplementa (Chrysomelidae: Galerucinae, Oidini, Galerucini, Metacyclini, Sermylini), Pars 78, Fasc. 1. 2^nd^ edition. Dr. W. Junk, Gravenhage, 1–220.

[B48] Wilcox JA (1972) Coleopterum Catalogus Supplementa (Chrysomelidae: Galerucinae, Luperini: Aulacophorina, Diabroticina), Pars 78, Fasc. 2. 2^nd^ edition. Dr. W. Junk, Gravenhage, 221–431.

[B49] Wilcox JA (1973) Coleopterum Catalogus Supplementa (Chrysomelidae: Galerucinae, Luperini: Luperina), Pars 78, Fasc. 3. 2^nd^ edition. Dr. W. Junk, Gravenhage, 433–664.

[B50] Wilcox JA (1975) Coleopterum Catalogus Supplementa (Chrysomelidae: Galerucinae Addenda et index). Pars 78, Fasc. 4. 2^nd^ edition. Dr. W. Junk, Gravenhage, 667–770.

[B51] Xue HJ Yang XK (2008) Common volatiles are major attractants for neonate larvae of the specialist flea beetle *Altica koreana* (Coleoptera: Chrysomelidae). Naturwissenschaften 95: 639–645. https://doi.org/10.1007/s00114-008-0367-y 18330535 10.1007/s00114-008-0367-y

[B52] Xue HJ Egas M Yang XK (2007) Development of a positive preference-performance relationship in an oligophagous beetle: adaptive learning? Entomologia Experimentalis et Applicata 125: 119–124. https://doi.org/10.1111/j.1570-7458.2007.00605.x

