# Supplementary material for: How many genera and species of Galerucinae s. str. do we know? Updated statistics (Coleoptera, Chrysomelidae)
Source: Zookeys. 2017 Dec 11;(720):91–102. doi: 10.3897/zookeys.720.13517 (PMC5740445; doi:10.3897/zookeys.720.13517)
Supplement: Supplementary material 1 — Genera list and related files [file zookeys-720-091-s001.doc]

Supplementary 1 The list of all valid genera (both recent and fossil) of subfamily Galerucinae s. str. in the world. All the listed genera were published before December 31, 2016. The references which taxonomically influenced the number of genera and species from Wilcox´s catalogues to present (including important redescriptions), including these omitted in Wilcox´s catalogue, are listed.

| **Genus** | **Present species** | **Present subspecies** | **Fossil species** | **Distribution** | **Subgenera (^∆^) and generic synonyms (**^*^**)** | **References** |
| --- | --- | --- | --- | --- | --- | --- |
| *Abdullahius* Abdullah and Qureshi, 1968 | 1 | 0 | 0 | PAR (Pakistan) |  | Abdullah and Qureshi (1968b) |
| *Acalymma* Barber, 1947 | 72 | 12 | 0 | NAR, NTR |  | Cabrera (1999b, 2001c), Gilbert and Clark (2007), Munroe and Smith (1980) |
| *Acroxena* Baly, 1879 | 9 | 0 | 0 | ORR | ^*^*Neochrolea* Jacoby, 1887 | Kimoto (1989a, 2003), Kimoto and Chu (1996), Kimoto and Takizawa (1972), Medvedev (1990, 1992a), Takizawa (1978) |
| *Adoxia* Broun, 1880 | 62 | 0 | 0 | AUR |  |  |
| *Aelianus* Jacoby, 1892 | 1 | 0 | 0 | AFR (Madagascar) |  |  |
| *Afrocandezea* Wagner and Scherz, 2002 | 12 | 0 | 0 | AFR |  | Bryant (1956), Scherz and Wagner (2007), Wagner and Scherz (2002) |
| *Afrocrania* Hincks, 1949 | 18 | 0 | 0 | AFR | ^*^*Pseudocrania* Weise, 1892 | Bryant (1956), Dalstein, Schulze and Wagner (2016), Middelhauve and Wagner (2001), Wagner (2007a) |
| *Afromaculepta* Hasenkamp and Wagner, 2000 | 6 | 0 | 0 | AFR, PAR (Yemen) |  | Hasenkamp and Wagner (2000) |
| *Afromegalepta* Schmitz and Wagner, 2001 | 1 | 0 | 0 | AFR |  | Schmitz and Wagner (2001) |
| *Afronaumannia* Steiner and Wagner, 2005 | 5 | 0 | 0 | AFR |  | Steiner and Wagner (2005) |
| *Afropachylepta* Esch and Wagner, 2009 | 1 | 0 | 0 | AFR |  | Esch and Wagner (2009) |
| *Afrorudolphia* Bolz and Wagner, 2014 | 2 | 0 | 0 | AFR |  | Bolz and Wagner (2014c), Selman (1963) |
| *Afrosoma* Wilcox, 1973 | 1 | 0 | 0 | AFR | ^*^*Exosomorpha* Laboissière, 1940 |  |
| *Afrotizea* Stapel and Wagner, 2001 | 1 | 0 | 0 | AFR |  | Stapel and Wagner (2001) |
| *Agelacida* Jacoby, 1898 | 1 | 0 | 0 | AFR |  |  |
| *Agelasa* Motschulsky, 1861 | 1 | 0 | 0 | PAR |  |  |
| *Agelastica* Chevrolat, 1836 | 6 | 1 | 0 | AFR?, AUR?, ORR?, PAR |  | Bezděk (2015a) |
| *Agelopsis* Jacoby, 1896 | 2 | 0 | 0 | ORR, PAR (Nepal) |  |  |
| *Agetocera* Hope, 1831 | 28 | 0 | 0 | ORR, PAR | ^*^*Aegelocerus* Hope, 1831 | Bezděk (2009d, 2010b), Chen (1964), Jiang (1992), Kimoto (1989a), Lee, Bezděk and Staines (2010), Medvedev (1981), Samoderzhenkov (1992), Yang, Ge and Li (2001), Yang, Li and Yao (1997), Zhang and Yang (2005a) |
| *Allastena* Broun, 1893 | 4 | 0 | 0 | AUR (New Zealand) |  |  |
| *Alopena* Baly, 1864 | 1 | 0 | 0 | AUR (New Guinea, Aru Is.) |  |  |
| *Alphidia* Clark, 1865 | 7 | 0 | 0 | AFR (Madagascar) | ^*^*Hovalia* Fairmaire, 1884 |  |
| *Amandus* Jacoby, 1886 | 1 | 0 | 0 | ORR (Ternate) |  |  |
| *Amphelasma* Barber, 1947 | 11 | 1 | 0 | NAR, NTR |  |  |
| *Anadimonia* Ogloblin, 1936 | 3 | 0 | 0 | ORR, PAR | ^*^*Trichocerophysa* Gressitt and Kimoto, 1963 | Kimoto (1989a), Medvedev (2005a) |
| *Anatela* Silfverberg, 1982 | 1 | 0 | 0 | AFR |  | Silfverberg (1982a) |
| *Androlyperus* Crotch, 1873 | 6 | 0 | 0 | NAR, NTR (Mexico) | ^*^*Malacamerus* Wilcox, 1951 | Clark (1999) |
| *Anisobrotica* Bechyné and Bechyné, 1970 | 5 | 1 | 0 | NTR |  | Cabrera (1991a) |
| *Anoides* Weise, 1913 | 8 | 0 | 0 | AUR | ^*^*Doryphoroides* Lea, 1925 |  |
| *Anomalonyx* Weise, 1903 | 2 | 0 | 0 | AFR |  |  |
| *Anthiphula* Jacoby, 1892 | 4 | 0 | 0 | ORR |  | Medvedev (2001b) |
| *Antsianaka* Duvivier, 1891 | 10 | 0 | 0 | AFR (Madagascar) |  |  |
| *Aplosonyx* Chevrolat, 1836 | 56 | 1 | 0 | ORR, PAR | ^*^*Haplonyx* Jacobson, 1896; ^*^*Berecyntha* Baly, 1865; ^*^*Caritheca* Baly, 1877 | Aslam (1972), Bezděk (2012b), Chen (1964), Jacoby (1895), Jiang (1992), Kimoto (1989a), Lopatin (2005c), Medvedev (2009a, 2015a), Mohamedsaid (1990, 1999c, 2008, 2011), Reid (1998b), Yang (1995a), Zhang et al (2008) |
| *Apophylia* Thomson, 1858 | 141 | 2 | 0 | AFR, AUR (New Guinea), ORR, PAR | ^*^*Malaxia* Fairmaire, 1878; ^*^*Glyptolus* Jacoby, 1884; ^*^*Galerucesthis* Weise, 1896; ^*^*Malaxioides* Fairmaire, 1888; ^*^*Bequaertinia* Laboissière, 1922 | Bezděk (2003a, 2003b, 2003c, 2003d, 2004b, 2004c, 2005a, 2005c, 2005d, 2005e, 2006a, 2006b, 2006c, 2007b, 2008b), Bezděk and Lee (2009), Bezděk and Zhang (2006a), Chen et al. (1976), Chûjô (1962), Kimoto (1977, 1989a), Lee and Bezděk (2014c), Medvedev (1992b, 1993), Medvedev and Sprecher-Uebersax (1998), Samoderzhenkov (1988), Takizawa (1985c) |
| *Apteraulamorphus* Beenen, 2010 | 1 | 0 | 0 | AFR (Malawi) |  | Beenen (2010a) |
| *Apterogaleruca* Chûjô, 1962 | 2 | 0 | 0 | PAR (China: Taiwan) |  |  |
| *Apteromicrus* Chen and Jiang, 1981 | 1 | 0 | 0 | PAR (China: Xizang) |  | Chen and Jiang (1981) |
| *Arcastes* Baly, 1865 | 6 | 0 | 0 | ORR |  | Hazmi and Wagner (2010b, 2010c), Mohamedsaid (2000a) |
| *Arima* Chapuis, 1875 | 4 | 7 | 0 | PAR |  |  |
| *Arimetus* Jacoby, 1903 | 5 | 0 | 0 | AFR |  | Aslam (1972) |
| *Aristobrotica* Bechyné, 1956 | 17 | 0 | 0 | NTR |  | Moura (1997, 2011) |
| *Arthrotidea* Chen, 1942 | 7 | 0 | 0 | PAR |  | Chen and Jiang (1981), Jiang (1988c), Kimoto and Takizawa (1972), Medvedev (2009f), Yang (1996) |
| *Arthrotus* Motschulsky, 1858 | 48 | 0 | 0 | ORR, PAR | ^*^*Dercestra* Chûjô, 1962;^*^*Cerotrus* Jacoby, 1884; ^*^*Anastena* Maulik, 1936; ^*^*Anicera* Jacoby, 1884, ^*^*Taphinella* Jacoby, 1889 | Beenen (2011a), Chen et al. (1976), Chûjô (1966), Jacoby (1889), Kimoto (1977, 1984, 1989a, 1996, 2004), Laboissière (1936b), Lee and Bezděk (2013b), Lopatin and Konstantinov (2009), Medvedev (1992b, 2000b, 2001d, 2012b), Medvedev and Sprecher-Uebersax (1998), Mohamedsaid (1997f, 1999d, 2001c, 2001e), Takizawa (1986b) |
| *Asbecesta* Harold, 1877 | 58 | 0 | 0 | AFR, PAR |  | Aslam (1972), Berti (1990b), Bryant (1956) |
| *Ashrafia* Abdullah and Qureshi, 1968 | 1 | 0 | 0 | PAR (Pakistan) |  | Abdullah and Qureshi (1968c) |
| *Astena* Baly, 1865 | 2 | 0 | 0 | AUR (New Guinea), ORR (India) |  |  |
| *Astridella* Laboissière, 1932 | 2 | 0 | 0 | AUR (New Guinea) |  |  |
| *Atrachya* Dejean, 1836 | 27 | 0 | 0 | AFR, ORR, PAR | ^*^*Iphidea* Baly, 1865; ^*^*Cnecodes* Motschulsky, 1858 | Jacoby (1892), Kimoto (1976, 1989a, 2005), Medvedev (2007d), Mohamedsaid (1999c), Takizawa (1978, 1985b), Wagner and Bieneck (2012) |
| *Atysa* Baly, 1864 | 29 | 0 | 0 | AUR, ORR, PAR | ^*^*Triaplatarthris* Fairmaire, 1878; ^*^*Formosogalerucella* Pic, 1928; ^*^*Falsoplatyxantha* Pic, 1927 | Aslam (1972), Beenen (2010b), Chen (1978), Kimoto (1982a, 1989a), Medvedev (2000b, 2005a, 2005b, 2013f), Mohamedsaid (2004), Beenen (2008a), Reid and Beatson (2010), Samoderzhenkov (1988) |
| *Aulacophora* Chevrolat, 1836 | 176 | 2 | 0 | AFR, AUR, PAC, PAR, ORR | ^*^*Triaplatys* Fairmaire, 1877; ^*^*Acutipalpa* Rosenhauer, 1856; ^*^*Ceratia* Chapuis, 1876; ^*^*Orthaulaca* Weise, 1892; ^*^*Cerania* Weise, 1892; ^*^*Sphaerarthra* Weise, 1892; ^*^ *Pachypalpa* Weise, 1892; ^*^*Raphidopalpa* Chevrolat, 1836; ^*^*Rhaphidopalpa* Rosenhauer, 1856 | Abdullah and Qureshi (1968a), Anand and Cox (1986), Barroga (2001a, 2001b, 2001c, 2002a, 2002b), Barroga and Mohamedsaid (2002), Beenen (2008b, 2010b, 2013a), Berti (1990b), Bezděk (2011), Chapuis (1876), Kimoto (1989a), Lee and Beenen (2015a), Medvedev (2001a, 2002a), Mohamedsaid (1994f, 1994h, 2009b), Ohno (1963), Samoderzhenkov (1992), Silfverberg (1978c) |
| *Aulamorphoides* Laboissière, 1926 | 1 | 0 | 0 | AFR (Congo) |  |  |
| *Aulamorphus* Jacoby, 1897 | 9 | 1 | 0 | AFR |  |  |
| *Austrochorina* Bechyné, 1963 | 1 | 0 | 0 | NTR |  |  |
| *Austrotella* Silfverberg, 1975 | 2 | 0 | 0 | AFR |  | Silfverberg (1975a) |
| *Azlania* Mohamedsaid, 1996 | 4 | 0 | 0 | ORR |  | Mohamedsaid (1996b, 1999e) |
| *Bacteriaspis* Weise, 1905 | 3 | 0 | 0 | AFR, PAR (Yemen) |  | Medvedev (2012d) |
| *Bangprella* Kimoto, 1989 | 5 | 0 | 0 | ORR |  | Kimoto (1989a), Medvedev (2007b), Medvedev and Romantsov (2013) |
| *# Barombiella* Laboissière, 1931 | 8 | 0 | 0 | AFR | ^*^*Barombia* Jacoby, 1903 | Aslam (1972), Bolz and Wagner (2012), Bryant (1958), Freund and Wagner (2003), Wagner and Freund (2003) |
| *Beenenia* Bezděk, 2012 | 2 | 0 | 0 | PAR (Socotra) |  | Bezděk (2012c) |
| *Beiratia* Jacoby, 1906 | 2 | 0 | 0 | AFR |  | Freund and Wagner (2003) |
| *Belarima* Reitter, 1913 | 1 | 0 | 0 | PAR |  |  |
| *Bicolorizea* Heunemann, Dalstein, Schulze and Wagner, 2015 | 4 | 0 | 0 | AFR |  | Bryant (1956), Heunemann et al (2015) |
| *Bipleura* Laboissière, 1932 | 2 | 0 | 0 | AFR (Madagascar) |  |  |
| *Bonesia* Baly, 1865 | 7 | 0 | 0 | AFR | ^*^*Aethonea* Baly, 1865 | Grobbelaar (2008) |
| *Bonesioides* Laboissière, 1925 | 21 | 0 | 0 | AFR |  | Aslam (1972), Freund and Wagner (2003) |
| Borneola Mohamedsaid, 1998 | 4 | 0 | 0 | ORR |  | Lee and Bezděk (20016b), Mohamedsaid (1998b), Takizawa (2011) |
| *Bradamina* Fairmaire, 1904 | 1 | 0 | 0 | AFR (Madagascar) |  |  |
| *Brachyphora* Jacoby, 1890 | 2 | 0 | 0 | PAR |  | Medvedev (1999) |
| *Brachyruca* Fairmaire, 1898 | 1 | 0 | 0 | AFR (Madagascar) |  |  |
| *Brucita* Wilcox, 1965 | 1 | 0 | 0 | NAR (Texas), NTR (Guatemala) |  |  |
| *Bryantiella* Medvedev, 2009 | 1 | 1 | 0 | AUR (Fiji) | ^*^*Bryantia* Medvedev, 2005 | Medvedev (2005b, 2009d) |
| *Bryobates* Broun, 1886 | 4 | 0 | 0 | AUR (New Zealand) |  |  |
| *Buckibrotica* Bechyné and Bechyné, 1970 | 1 | 0 | 0 | NTR |  |  |
| *Buphonella* Jacoby, 1903 | 4 | 1 | 0 | AFR |  | Biondi and D´Alessandro (2012), Wilcox (1975) |
| *Buphonida* Baly, 1865 | 6 | 0 | 0 | AUR (New Guinea), ORR |  | Medvedev (2002a) |
| *Byblitea* Baly, 1864 | 6 | 0 | 0 | NTR |  |  |
| *Calaina* Schaufuss, 1887 | 2 | 0 | 0 | ORR (Sulawesi) |  | Aslam (1972) |
| *Calomicrella* Medvedev and Bezděk, 2002 | 1 | 0 | 0 | ORR |  | Medvedev and Bezděk (2002) |
| *Calomicroides* Nadein, 2016 | 0 | 0 | 1 | fossil, Danish amber |  | Nadein, Perkovsky and Moseyko (2016) |
| *Calomicrus* Dillwyn, 1829 | 85 | 2 | 1 | AFR, ORR, PAR | ^*^*Trichelytron* Apfelbeck, 1912 | Apfelbeck (1912), Beenen (2010b, 2010c, 2011b), Bezděk (1998, 2006d, 2007a, 2012b, 2013a, 2016d), Bezděk, Sen and Gök (2013), Bolz and Wagner (2005), Borowiec (2005), Bukejs and Bezděk (2014), Chen and Jiang (1981), Csiki (1940), Gök, Aslan and Aslan (2005), Israelson (1980), Kimoto (1977, 1983, 1984, 1989a, 1996, 2004), Lee and Beenen (2012), Lopatin (1975, 1983, 1984, 1988, 1990, 2001, 2002a, 2002c, 2005c, 2006b, 2006c), Lopatin and Konstantinov (2009), Lopatin and Nesterova (2006, 2013), Medvedev (1974, 1975, 1985, 1992b, 1996, 1998b, 1999, 2011b, 2013a), Medvedev and Sprecher-Uebersax (1998, 1999), Takizawa (1988a, 1988b), Tomov (1975), Vela and Bastazo (1990), Vela and García Beccera (1996), Wagner and Bieneck (2012), Warchałowski (1991) |
| *Candezeososia* Laboissière, 1932 | 1 | 0 | 0 | AFR (Madagscar) |  |  |
| *Candezea* Chapuis, 1879 | 40 | 1 | 0 | AFR, AUR | ^*^*Alaotra* Duvivier, 1891 | Aslam (1972), Beenen (2013a), Wagner (2011), Wagner and Kurtscheid (2005) |
| *Candezoides* Duvivier, 1891 | 1 | 0 | 0 | AFR (Madagscar) |  |  |
| *Cannonia* Hincks, 1949 | 4 | 1 | 0 | AFR | ^*^*Belona* Weise, 1901 | Silfverberg (1971) |
| *Capula* Jacobson, 1925 | 3 | 0 | 0 | PAR |  | Chen, Wang and Jiang (1986b) |
| *Caraguata* Bechyné, 1954 | 38 | 6 | 0 | NTR |  | Bechyné and Springlová-Bechyné (1969, 1970), Cabrera and Durante (2004), Moura (2005, 2016) |
| *Cassena* Weise, 1892 | 49 | 1 | 0 | AUR, PAR, ORR | ^∆^*Nepalocassena Medvedev*, 2009. ^*^*Euphyma* Baly, 1879; ^*^*Solenia* Jacoby, 1886; ^*^*Solephyma* Maulik, 1936; ^*^*Taphinellina* Maulik, 1936 | Aslam (1972), Beenen (2010b), Bezděk (2016a), Kimoto (1989a), Lee and Bezdek (2016c), Medvedev (1995, 2009b), Takizawa (1986b, 1988b) |
| *Cassenoides* Kimoto, 1989 | 1 | 0 | 0 | ORR (Thailand) |  | Kimoto (1989a) |
| *Ceratocalymma* Hincks, 1949 | 1 | 0 | 0 | AUR (New Guinea) | ^*^*Ceratotrix* Weise, 1917 |  |
| *Cerochroa* Gerstaecker, 1855 | 12 | 0 | 0 | AFR |  |  |
| *Cerophysa* Chevrolat, 1836 | 47 | 0 | 0 | AUR (Fiji), ORR, PAR | ^*^*Cerophyta* Strand, 1935; ^*^*Ozomena* Chevrolat, 1845; ^*^*Oedicerus* Kollar and Redtenbacher, 1844; ^*^*Taumaceroides* Lopatin, 2011 | Beenen (2005a, 2008a, 2010b, 2010c), Bezděk (2012b), Kimoto (1977, 1989a, 2004, 2005), Lee (2014a), Lee, Beenen and Staines (2009), Lee and Bezdek (2016a), Lopatin and Konstantinov (2009), Medvedev (2012b), Medvedev and Sprecher-Uebersax (1998), Mohamedsaid (1996c, 2001a, 2001e, 2003), Takizawa and Basu (1987) |
| *Cerophysella* Laboissière, 1930 | 6 | 0 | 0 | ORR, PAR | ^*^*Chogania* Laboissière, 1930 | Kimoto (1989a), Medvedev (2015b), Mohamedsaid (2001e) |
| *Cerotoma* Chevrolat, 1836 | 14 | 6 | 0 | NAR, NTR | ^*^*Andrector* Horn, 1872 | Ruppel (1978) |
| *Clerotilia* Jacoby, 1885 | 8 | 0 | 0 | PAR |  | Kimoto and Chu (1996), Lopatin (2002c), Takizawa (1978) |
| *Clitena* Baly, 1864 | 2 | 0 | 0 | ORR, PAR (China: Gansu) | ^*^*Mesodonta* Baly, 1865 |  |
| *Clitenella* Laboissière, 1927 | 7 | 0 | 0 | ORR, PAR | ^*^*Callopistria* Chevrolat, 1836 | Mohamedsaid (2001c), Samoderzhenkov (1988), Yang, Li and Yao (1997), Zhang, Beenen and Yang (2008) |
| *Clitenososia* Laboissière, 1931 | 4 | 0 | 0 | AFR |  | Beenen (2014a) |
| *Cneorane* Baly, 1865 | 42 | 0 | 0 | AUR (New Guinea), PAR, ORR |  | Beenen (2005b, 2008a, 2010b), Chen (1964), Chen et al. (1976), Chûjô (1966), Jacoby (1894), Kimoto (1977, 2004), Kimoto and Takizawa (1972), Lee and Bezděk (2013c), Medvedev (1992b, 2002a, 2004d, 2009b, 2011a, 2013d) |
| *Cneoranidea* Chen, 1942 | 11 | 0 | 0 | PAR, ORR | ^*^*Neocrane* Chûjô, 1962 | Bezděk (2010b), Chen and Jiang (1984), Kimoto (1976, 1989a), Lee (2011), Medvedev (2005b), Yang (1991d, 1992d), Yang, Wang and Wu (1998), Yang et al (1997), Zhang and Yang (2005b) |
| *Cneorella* Medvedev and Dang, 1981 | 14 | 0 | 0 | ORR, PAR |  | Bezděk (2005b, 2012b), Kimoto (1989a), Lee and Bezděk (2012), Lopatin (2003b), Medvedev (2000c), Medvedev and Dang (1981) |
| *Cneorides* Jacoby, 1896 | 1 | 0 | 0 | ORR |  |  |
| *Coeligetes* Jacoby, 1884 | 5 | 0 | 0 | ORR |  | Bezděk (2016b), Mohamedsaid (1994a) |
| *Coeligetoides* Bezděk, 2016 | 1 | 0 | 0 | ORR |  | Bezděk (2016b) |
| *Coelocrania* Jacoby, 1886 | 2 | 0 | 0 | AUR (New Guinea), ORR (Seram) |  | Mohamedsaid (1993c), Shute (1983) |
| *Coelomera* Chevrolat, 1836 | 33 | 2 | 0 | NTR |  | Bechyné and Springlová-Bechyné (1970), Moura (2009) |
| *Cochabamba* Bechyné, 1955 | 10 | 0 | 0 | NTR |  | Cabrera (1999a), Moura (2003) |
| *Conchocera* Laboissière, 1922 | 1 | 0 | 0 | AFR |  |  |
| *Coraia* Clark, 1865 | 4 | 0 | 0 | NTR (Middle America) |  |  |
| *Cornubrotica* Bechyné and Bechyné, 1970 | 2 | 0 | 0 | NTR |  | Moura (2005) |
| *Coronabrotica* Moura, 2010 | 1 | 0 | 0 | NTR |  | Moura (2010) |
| *Crampelia* Laboissière, 1922 | 2 | 0 | 0 | AFR |  |  |
| *Craniotectus* Laboissière, 1932 | 1 | 0 | 0 | ORR (Peninsular Malaysia) |  |  |
| *Cyclantipha* Laboissière, 1932 | 2 | 0 | 0 | ORR |  | Kimoto (1989a), Medvedev (2004a) |
| *Cyclotrypema* Blake, 1966 | 1 | 0 | 0 | NAR (Texas), NTR (Mexico) |  |  |
| *Cydippa* Chapuis, 1875 | 1 | 0 | 0 | AUR |  | Beenen and Hawkeswood (2004) |
| *Cynortella* Duvivier, 1891 | 2 | 0 | 0 | AFR (Madagscar) |  |  |
| *Cynortina* Weise, 1905 | 1 | 0 | 0 | AFR (Zimbabwe) |  |  |
| *Decarthrocera* Laboissière, 1937 | 1 | 0 | 0 | PAR (Algeria) |  | Biondi and D´Alessandro (2012), Furth and Suzuki (1994), Wilcox (1975) |
| *Deinocladus* Blake, 1966 | 3 | 0 | 0 | NTR |  |  |
| *Dercetina* Gressitt and Kimoto, 1963 | 90 | 0 | 0 | ORR, PAR | ^*^*Dercetes* Hincks, 1949; ^*^*Dercetis* Clark, 1865; ^*^*Antipha* Baly, 1865 | Jacoby (1886), Kimoto (1970a, 1977, 1989a, 2001, 2004), Kimoto and Takizawa (1972), Lee and Bezděk (2013b), Medvedev (1995, 2000c, 2001d, 2011c), Medvedev and Sprecher-Uebersax (1998), Mohamedsaid (1995c, 1997f, 1999c, 1999d, 2001e), Takizawa (1985a, 1990) |
| *Dercetisoma* Maulik, 1936 | 7 | 0 | 0 | ORR, PAR |  | Beenen (2008a, 2010b), Kimoto (1989a, 2004), Medvedev (2012b), Medvedev and Sprecher-Uebersax (1998), Mohamedsaid (2002b) |
| *Derospidea* Blake, 1931 | 3 | 0 | 0 | NAR, NTR (Mexico) |  |  |
| *Desbordesius* Laboissière, 1933 | 2 | 0 | 0 | ORR |  | Kimoto (1989a) |
| *Diabrotica* Chevrolat, 1836 | 370 | 27 | 4 | NAR, NTR |  | Bechyné (1971, 1997), Bechyné and Springlová-Bechyné (1969, 1970, 1976), Blake (1971), Cabrera (2001a, 2001b), Cabrera and Cabrera Walsh (2004b, 2010), Cabrera, Sosa Gómez and Micheli (2008), Derunkov and Konstantinov (2013), Derunkov, Konstantinov and Tishechkin (2013), Derunkov et al (2015), ICZN (1993), Krysan and Smith (1987a, 1987b), Krysan, Smith and Guss (1983), Krysan et al (1980, 1984), Tubbs (1991) |
| *Diacantha* Chevrolat, 1836 | 88 | 0 | 0 | AFR, PAR (Yemen) | ^*^*Hyperacantha* Chapuis, 1880; ^*^*Idacantha* Fairmaire, 1869; ^*^*Corynopalpa* Dejean, 1836 | Bechyné (1964), Beenen (2008a, 2014b, 2016), Berti (1990b), Gahan (1896) |
| *Dicoelotrachelus* Blake, 1941 | 5 | 0 | 0 | NTR (Carribean Islands) |  |  |
| *Dilinosa* Weise, 1906 | 3 | 0 | 0 | AFR |  |  |
| *Dimalianella* Laboissière, 1940 | 7 | 0 | 0 | AFR | ^*^*Dimalia* Laboissière, 1926 |  |
| *Diorhabda* Weise, 1883 | 11 | 0 | 0 | ORR (India), PAR |  | Aslam (1972), Beenen (2008a, 2010b, 2011a, 2014c), Berti and Rapilly (1973), Kimoto and Takizawa (1981), Medvedev (2001d), Reid and Nally (2008), Silfverberg (1974), Tracy and Robbins (2009) |
| *Dircema* Clark, 1865 | 25 | 2 | 0 | NTR |  | Bechyné (1997), Moura (2009) |
| *Dircemella* Weise, 1902 | 4 | 0 | 0 | AFR |  | Aslam (1972), Beenen (2016) |
| *Doryida* Baly, 1865 | 5 | 0 | 0 | ORR, PAR |  | Kimoto (1989a), Wilcox (1975) |
| *Doryidella* Laboissière, 1940 | 3 | 0 | 0 | ORR |  | Kimoto (1989a), Medvedev (2002a, 2015b) |
| *Doryidomorpha* Laboissière, 1931 | 7 | 0 | 0 | ORR, PAR |  | Medvedev (2007a) |
| *Doryscus* Jacoby, 1887 | 6 | 0 | 0 | ORR, PAR |  | Jiang (1992), Kimoto (1991b), Medvedev and Sprecher-Uebersax (1998), Mohamedsaid (1999c), Takizawa (1978) |
| *Doryxena* Baly, 1861 | 3 | 0 | 0 | ORR, PAR |  | Takizawa (1990), Kimoto (2004) |
| *Doryxenoides* Laboissière, 1927 | 3 | 0 | 0 | ORR, PAR |  | Takizawa (1990) |
| *Drasa* Bryant, 1941 | 13 | 0 | 0 | AUR (Solomon Islands, New Britain, New Hebrides) | ^*^*Sarda* Baly, 1865; ^*^*Callisarda* Hincks, 1949 | Aslam (1972), Vachon (1977) |
| *Dreeus* Shute, 1983 | 1 | 0 | 0 | AUR (New Guinea) |  | Shute (1983) |
| *Duvivieria* Weise, 1903 | 5 | 0 | 0 | AFR |  | Bryant (1956) |
| *Dyolania* Laboissière, 1931 | 5 | 0 | 0 | AFR |  | Aslam (1972), Bauer and Wagner (2010) |
| *Dyserythra* Weise, 1902 | 1 | 0 | 0 | AFR (Tamzania) | ^*^*Hyperyrthra* Weise, 1902 |  |
| *Dysiodes* Weise, 1908 | 1 | 0 | 0 | AUR (New Guinea) |  |  |
| *Eccoptopsis* Blake, 1966 | 12 | 0 | 0 | NTR |  |  |
| *Ectmesopus* Blake, 1940 | 15 | 0 | 0 | NTR (Carribean Islands) |  |  |
| *Eleona* Fairmaire, 1902 | 3 | 0 | 0 | AFR (Madagascar) |  | Bechyné (1964) |
| *Ellopidia* Hincks, 1949 | 4 | 0 | 0 | AUR | ^*^*Ellopia* Chapuis, 1875 |  |
| *Elyces* Jacoby, 1888 | 6 | 0 | 0 | NTR |  |  |
| *Emathea* Baly, 1865 | 10 | 0 | 0 | ORR, PAR |  | Kimoto (1989a) |
| *Ensiforma* Jacoby, 1876 | 9 | 0 | 0 | NTR |  |  |
| *Epaenidea* Gressitt and Kimoto, 1963 | 3 | 0 | 0 | ORR, PAR |  | Medvedev (2004b) |
| *Epiluperodes* Gressitt and Kimoto, 1963 | 1 | 0 | 0 | PAR (Ryukyu Islands) |  |  |
| *Erganoides* Jacoby, 1903 | 11 | 0 | 0 | PAR |  | Beenen (2008a, 2010c), Beenen and Lee (2010), Kimoto (1977, 1989a, 2000a, 2000b), Medvedev (2007d) |
| *Erynephala* Blake, 1936 | 6 | 0 | 0 | NAR, NTR | ^*^*Sarigueia* Bechyné, 1956 | Blake (1970a) |
| *Erythrobapta* Weise, 1902 | 8 | 0 | 0 | AFR |  | Beenen (2016) |
| *Estcourtiana* Jacoby, 1900 | 6 | 0 | 0 | AFR |  |  |
| *Eubeiratia* Laboissière, 1931 | 1 | 0 | 0 | AFR (Mozambique) |  |  |
| *Eucerotoma* Laboissière, 1939 | 22 | 1 | 0 | NTR |  | Bechyné and Springlová-Bechyné (1970) |
| *Eugaleruca* Laboissière, 1922 | 5 | 0 | 0 | AFR | ^*^*Protogaleruca* Laboissière, 1932 | Aslam (1972) |
| *Euliroetis* Ogloblin, 1936 | 9 | 0 | 0 | ORR (Vietnam), PAR |  | Yang (1992e) |
| *Euluperus* Weise, 1886 | 7 | 1 | 0 | AFR, PAR |  | Bezděk (2015a), Lopatin (1997), Medvedev (2012e) |
| *Eumelepta* Jacoby, 1892 | 2 | 0 | 0 | ORR, PAR (Nepal) |  | Beenen (2011a), Kimoto (1977) |
| *Eupachytoma* Laboissière, 1940 | 11 | 0 | 0 | AFR | ^*^*Pachytoma* Clark, 1865 | Aslam (1972), Beenen (2002, 2012), Mohamedsaid (1998a) |
| *Eurycycla* Jacoby, 1903 | 1 | 0 | 0 | AFR |  |  |
| *Eusattodera* Schaeffer, 1906 | 6 | 0 | 0 | NAR, NTR (Mexico) |  |  |
| *Eustena* Baly, 1879 | 1 | 0 | 0 | ORR (Assam) |  |  |
| *Exora* Chevrolat, 1836 | 14 | 6 | 0 | NTR |  | Moura (2009) |
| *Exosoma* Jacoby, 1903 | 86 | 0 | 0 | AFR, ORR, PAR | ^*^*Malacosoma* Chevrolat, 1836; ^*^*Malacoptera* Hope, 1840; ^*^*Malacodora* Bedel, 1905 | Aslam (1972), Beenen (2016), Bryant (1956, 1958), Chûjô (1966), Jacoby (1884), Kimoto (1996, 2001, 2004), Kortenhaus and Wagner (2010, 2011, 2013), Medvedev (1998b), Schmitz and Wagner (2001), Takizawa (1990), Wilcox (1975) |
| *Exosomella* Laboissière, 1932 | 1 | 0 | 0 | AFR (Madagascar) |  |  |
| *Exosomorpha* Laboissière, 1932 | 2 | 0 | 0 | AFR (Madagascar) |  |  |
| *Falsoexosoma* Pic, 1926 | 1 | 0 | 0 | PAR |  |  |
| *Farsogaleruca* Lopatin, 1981 | 2 | 0 | 0 | PAR (Iran) |  | Lopatin (1981) |
| *Fleutiauxia* Laboissière, 1933 | 11 | 1 | 0 | ORR, PAR |  | Kimoto (1989a), Yang (1993b), Yang and Li (1998), Yang et al (1997) |
| *Furusawaia* Chûjô, 1962 | 2 | 0 | 0 | PAR |  | Lopatin (2008b) |
| *Galenaria* Medvedev, 2007 | 1 | 0 | 0 | ORR (Laos) |  | Medvedev (2007c) |
| *Galerosastra* Laboissière, 1929 | 2 | 0 | 0 | ORR (Sumatra) |  |  |
| *Galerotella* Maulik, 1936 | 4 | 0 | 0 | ORR |  | Aslam (1972), Kimoto (1979), Takizawa (1986b) |
| *Galeruca* Geoffroy, 1762 | 71 | 12 | 0 | NAR, ORR (India), PAR | ^∆^*Galemira* Beenen, 2003; ^∆^*Galerima* Reitter, 1903; ^∆^*Emarhopa* Weise, 1886; ^∆^*Galerotoma* Reitter, 1903; ^∆^*Haptoscelis* Weise, 1886; ^∆^*Fassatia* Havelka, 1954. ^*^*Adimonia* Laicharting, 1781 | Beenen (1999, 2002, 2003a, 2005b, 2007b, 2007c, 2008a, 2010b), Beenen and Yang (2007), Berti and Rapilly (1983), Chen and Jiang (1981, 1987a), Franz (1974), ICZN (1994), Kimoto (1983), Mandl (1970, 1976, 1981, 1986), Medvedev (1973, 2011b), Medvedev and Mirzoeva (1969), Medvedev and Sprecher-Uebersax (1997), Silfverberg (1978d), Yang, Li and Yao (1997) |
| *Galerucella* Crotch, 1873 | 39 | 1 | 1 | AUR, ORR, PAR, NAR, NTR | ^∆^*Neogalerucella* Chûjô, 1962. ^*^*Hydrogaleruca* Laboissière, 1922 | Aslam (1972), Bechyné and Springlová-Bechyné (1970), Beenen (1998, 2008a, 2010b, 2014c), Blake (1970b, 1971), Kimoto (1979, 1989a), Kimoto and Takahashi (1992), Komiya (2005), Lohse (1989), Lopatin (2002b), Makhan (2012), Medvedev (2015a), Mohamedsaid (1999c), Moura (1998c, 2016), Reid (2001b), Reid and Nally (2008), Samoderzhenkov (1988), Shute (1983), Silfverberg (1974, 1990), Xue and Yang (2010) |
| *Galerudolphia* Hincks, 1949 | 16 | 0 | 0 | AFR, PAR (Arabian Peninsula) | ^*^*Rudolphia* Jacoby, 1899 | Bolz and Wagner (2005), Medvedev (1996) |
| *Galerumaea* Hincks, 1949 | 12 | 0 | 0 | AUR, ORR | ^*^*Eumaea* Baly, 1865 | Aslam (1972), Jaocby (1886), Shute (1983), Wilcox (1975) |
| *Galerusoma* Jacoby, 1892 | 1 | 0 | 0 | AFR (Madagascar) |  |  |
| *Gallerucida* Motschulsky, 1861 | 87 | 0 | 0 | ORR, PAR | ^∆^*Coptomesa* Weise, 1912; ^*^*Eustetha* Baly, 1861; ^*^*Melospila* Baly, 1861; ^*^*Stethidea* Baly, 1890; ^*^*Hylaspes* Baly, 1865 | Beenen (2010b), Bezděk (2012b), Chen and Yang (1992), Kimoto (1989a), Kimoto and Chu (1996), Lee and Bezděk (2013a), Lopatin (2004b, 2005c), Medvedev (2003a, 2009a), Takizawa (1978, 1988b), Yang (1992c, 1992e, 1994a, 1994b), Yang et al (1997) |
| *Gastrida* Chapuis, 1879 | 5 | 1 | 0 | AFR |  | Furth and Suzuki (1994), Wilcox (1975) |
| *Geinella* Strand, 1935 | 13 | 0 | 0 | PAR | ^*^*Geina* Jacobson, 1925; ^*^*Swargia* Maulik, 1936 | Beenen (2011a), Chen and Jiang (1981, 1987a, 1987b), Chen, Jiang and Wang (1987), Warchałowski (2001) |
| *Geinula* Ogloblin, 1936 | 9 | 0 | 0 | PAR |  | Chen, Jiang and Wang (1987), Lopatin (2008b) |
| *Glaucorhabda* Weise, 1910 | 3 | 0 | 0 | AFR (Madagascar) | ^*^*Antsianacida* Laboissière, 1932 | Aslam (1972) |
| *Gonaives* Clark, 1987 | 1 | 0 | 0 | NTR (Haiti) |  | Clark (1987) |
| *Goudotina* Weise, 1910 | 5 | 0 | 0 | AFR (Madagascar) |  |  |
| *Gronovius* Jacoby, 1905 | 1 | 0 | 0 | AUR (New Guinea) |  | Shute (1983) |
| *Gynandrobrotica* Bechyné, 1955 | 31 | 7 | 0 | NTR |  | Bechyné (1997) |
| *Haemodoryida* Chen, 1942 | 1 | 0 | 0 | PAR (China: Xizang) |  |  |
| *Halinella* Bechyné, 1956 | 9 | 0 | 0 | NTR |  | Bechyné (1997) |
| *Hallirhotius* Jacoby, 1888 | 6 | 1 | 0 | AFR |  | Beenen (2016) |
| *Halticopsis* Fairmaire, 1883 | 1 | 0 | 0 | AFR (Ethiopia) |  | Biondi and D´Alessandro (2012) |
| *Halysacantha* Laboissière, 1922 | 1 | 0 | 0 | AFR |  |  |
| *Hamushia* Chûjô, 1956 | 2 | 0 | 0 | PAR (Japan) |  |  |
| *Haplosomoides* Duvivier, 1890 | 30 | 1 | 0 | ORR, PAR | ^*^*Hoplasomedia* Maulik, 1936 | Basu (1985), Beenen (2010b), Bezděk (2009a), Bezděk and Zhang (2007), Jiang (1988a), Kimoto (1984, 1989a), Lee and Bezděk (2015), Lee, Bezděk and Staines (2011), Medvedev (2000d, 2002b), Mohamedsaid (1994c, 1994g, 2001e, 2007), Takizawa (1985a) |
| *Haplotia* Jacoby, 1887 | 1 | 0 | 0 | ORR (Sri Lanka) |  |  |
| *Hatita* Fairmaire, 1891 | 2 | 0 | 0 | AFR |  |  |
| *Hecataeus* Jacoby, 1888 | 3 | 0 | 0 | NTR |  | Bechyné (1997) |
| *Hemiphracta* Weise, 1902 | 3 | 0 | 0 | AFR |  | Aslam (1972) |
| *Hemistus* Jacoby, 1886 | 1 | 0 | 0 | ORR (Borneo) |  |  |
| *Hemygascelis* Jacoby, 1896 | 1 | 0 | 0 | ORR |  |  |
| *Hesperomorpha* Ogloblin, 1936 | 8 | 0 | 0 | ORR, PAR |  | Kimoto (1970b, 1996, 2000a, 2000b) |
| *Hesperopenna* Medvedev and Dang, 1981 | 36 | 1 | 0 | ORR, PAR | ^*^*Liroetiella* Kimoto, 1989; ^*^*Levnma* Özdikmen, 2008; ^*^*Martinella* Medvedev, 2000 | Bezděk (2013a, 2016d), Kimoto (1977, 1989a, 2004), Lopatin (2013), Medvedev (1995, 2000c, 2002a, 2007b, 2012c, 2013a, 2013c), Medvedev and Dang (1981), Medvedev and Romantsov (2013), Mohamedsaid (1998g, 2001e), Mohamedsaid and Kimoto (1993), Özdikmen (2008), Warchałowski (1991) |
| *Hildebrandtianella* Laboissière, 1932 | 1 | 0 | 0 | AFR (Madagascar) |  |  |
| *Himaplosonyx* Chen, 1976 | 1 | 0 | 0 | PAR (China: Xizang) |  | Chen et al (1976) |
| *Hirtigaleruca* Chûjô, 1962 | 1 | 0 | 0 | PAR (China: Taiwan) |  |  |
| *Hirtomimastra* Medvedev, 2009 | 1 | 0 | 0 | ORR (Vietnam) |  | Medvedev (2009a) |
| *Hoplasoma* Jacoby, 1884 | 36 | 0 | 0 | ORR, PAR | ^*^*Paraulacophora* Csiki, 1953; ^*^*Haplomela* Chen, 1942 | Bezděk (2006e, 2008a, 2010b, 2010c, 2012a, 2012b, 2014), Bezděk and Zhang (2007), Jiang (1988a), Kimoto (1970a, 1977, 1989a), Medvedev (2000a, 2000c, 2002d, 2004b, 2005b, 2007a), Samoderzhenkov (1992), Silfverberg (1978c), Takizawa (1987) |
| *Hoplosaenidea* Laboissière, 1933 | 87 | 0 | 0 | AUR, ORR, PAR | ^*^*Micraenidea* Laboissière, 1933; ^*^*Cynorita* Laboissière, 1940; ^*^ *Diaphaenidea* Laboissière, 1933; ^*^*Cynorta* Baly, 1865; ^*^*Cynortana* Strand, 1942 | Beenen (2008a, 2010b), Bezděk (2009a), Chûjô (1966), Kimoto (1977, 1984, 1989a, 1989c, 2003), Lee (2014a), Lee, Beenen and Staines (2009), Medvedev (1974, 1995, 2011c, 2016), Mohamedsaid (1995c, 1997a, 1997d, 1998c, 1998e, 1999c, 1999d, 2000a, 2000c, 2001c, 2001e, 2002a, 2004, 2009a), Reid (2003), Takizawa (1985a, 1988b), Takizawa and Mohamedsaid (2015) |
| *Hoplostines* Blackburn, 1890 | 1 | 0 | 0 | AUR |  | Reid (2001b) |
| *Hovaliana* Laboissière, 1932 | 1 | 0 | 0 | AFR (Madagascar) |  |  |
| *Huillania* Laboissière, 1921 | 4 | 0 | 0 | AFR |  | Aslam (1972) |
| *Hylaspoides* Duvivier, 1892 | 1 | 0 | 0 | PAR (Sikkim) |  |  |
| *Hymenesia* Clark, 1865 | 1 | 0 | 0 | ORR |  |  |
| *Hyperbrotica* Bechyné and Bechyné, 1968 | 1 | 1 | 0 | NTR |  |  |
| *Hyphaenia* Baly, 1865 | 53 | 0 | 0 | ORR, PAR | ^*^*Trichocerastes* Motschulsky, 1866 | Jacoby (1886, 1894), Kimoto (1989a, 2003), Lopatin (2004b, 2006a, 2013), Lopatin and Konstantinov (2009), Medvedev (2000b, 2001b, 2007d, 2008a, 2011a, 2012b, 2014a, 2015b), Medvedev and Romantsov (2013), Medvedev and Sprecher-Uebersax (1997), Mohamedsaid (1998g, 1999c), Takizawa (1985b, 1988b) |
| *Hystiopsis* Blake, 1966 | 19 | 0 | 0 | NTR |  |  |
| *Chapuisia* Duvivier, 1885 | 54 | 0 | 0 | AFR | ^*^*Paralepta* Chapuis, 1879; ^*^*Hemixantha* Jacoby, 1899 | Aslam (1972), Bezděk (2006c), Brynt (1960) |
| *Charaea* Baly, 1878 | 43 | 0 | 0 | ORR, PAR |  | Beenen (1992, 2010b), Beenen and Warchałowski (2010), Bezděk (2012b, 2015b), Bezděk and Lee (2014), Chûjô (1966), Kimoto (1983, 1996, 2001, 2004), Lopatin and Konstantinov (2009), Medvedev (1998b, 2011a), Medvedev and Sprecher-Uebersax (1998), Takizawa (1986b, 1988a) |
| *Chlamigala* Medvedev and Bezděk, 2002 | 1 | 0 | 0 | ORR (Thailand) |  | Medvedev and Bezděk (2002) |
| *Chlorolochmaea* Bechyné and Bechyné, 1969 | 1 | 0 | 0 | NTR |  | Moura (1998a) |
| *Chorina* Baly, 1866 | 3 | 0 | 0 | NTR |  |  |
| *Chosnia* Berti, 1990 | 3 | 0 | 0 | AFR |  | Berti (1990b) |
| *Chthoneis* Baly, 1864 | 28 | 1 | 0 | NTR |  | Bechyne and Springlova de Bechyne (1961, 1967, 1970) |
| *Chujoa* Gressitt and Kimoto, 1963 | 1 | 0 | 0 | PAR (Japan) |  |  |
| *Ikopista* Fairmaire, 1901 | 1 | 0 | 0 | AFR (Madagascar) |  |  |
| *Impensa* Wilcox, 1971 | 2 | 0 | 0 | AFR |  | Berti (1987, 1988) |
| *Inbioluperus* Clark, 1993 | 2 | 0 | 0 | NTR (Costa Rica) |  | Clark (1993) |
| *Interbrotica* Bechyné and Bechyné, 1965 | 1 | 0 | 0 | NTR |  |  |
| *Isotes* Weise, 1922 | 181 | 5 | 0 | NTR | ^*^*Synbrotica* Bechyné, 1956 | Aslam (1972), Cabrera (1995), Moura (2003, 2009), Rodrigues and Mermudes (2015) |
| *Itaitubana* Bechyné, 1963 | 9 | 0 | 0 | NTR |  | Cabrera (1991b) |
| *Itylus* Jacoby, 1904 | 5 | 0 | 0 | ORR |  | Medvedev and Romantsov (2014), Mohamedsaid (1995a) |
| *Iucetima* Moura, 1998 | 3 | 0 | 0 | NTR |  | Moura (1998b) |
| *Jacobya* Weise, 1901 | 5 | 0 | 0 | AFR |  |  |
| *Jacobyanella* Laboissière, 1924 | 11 | 0 | 0 | AFR (Madagascar) |  | Bechyné (1964) |
| *Japonitata* Strand, 1935 | 33 | 0 | 0 | ORR, PAR | ^*^*Japonia* Weise, 1922 | Beenen (2011c), Bezděk (2012b), Chen and Jiang (1981, 1986a), Jiang (1989), Kimoto (1970a, 1996, 2004), Medvedev (2000d, 2002b, 2012b), Medvedev and Sprecher-Uebersax (1998, 1999), Takizawa and Basu (1987), Wu, Yang and Li (1998), Yang (1992e), Yang and Li (1998), Yang et al (1997), Zhang, Li and Yang (2008b) |
| *Jolibrotica* Lee and Bezděk, 2015 | 2 | 0 | 0 | PAR (China: Taiwan) |  | Lee and Bezděk (2015) |
| *Kanahiiphaga* Laboissière, 1931 | 7 | 0 | 0 | AFR |  | Laboissière (1936a) |
| *Kanarella* Jacoby, 1896 | 2 | 0 | 0 | ORR | ^*^*Cneoranella* Maulik, 1936 |  |
| *Keitheatus* Wilcox, 1965 | 1 | 0 | 0 | NAR (Texas) |  | Andrews and Gilbert (2005) |
| *Khasia* Jacoby, 1899 | 5 | 0 | 0 | AUR (Fiji), ORR | ^*^*Harmandinia* Laboissière, 1932 | Aslam (1972), Kimoto (1984), Kimoto and Chu (1996), Mohamedsaid (2010) |
| *Kinabalua* Mohamedsaid, 1997 | 2 | 0 | 0 | ORR (Borneo) |  | Mohamedsaid (1997b, 2010) |
| *Konbirella* Duvivier, 1892 | 1 | 0 | 0 | ORR |  |  |
| *Kumbalia* Mohamedsaid and Takizawa, 2007 | 1 | 0 | 0 | ORR (Bali) |  | Mohamedsaid and Takizawa (2007) |
| *Kumbornia* Mohamedsaid, 2006 | 1 | 0 | 0 | ORR (Borneo) |  | Mohamedsaid (2006) |
| *Laetana* Baly, 1864 | 1 | 0 | 0 | AFR |  | Silfverberg (1975a, 1978a, 1982a) |
| *Laetiacantha* Laboissière, 1921 | 11 | 1 | 0 | AFR | ^∆^*Zinjotella* Silfverberg, 1975 | Silfverberg (1973b, 1975b, 1975c, 1982b) |
| *Lamprocopa* Hincks, 1949 | 10 | 0 | 0 | AFR, PAR (Yemen) | ^*^*Copa* Weise, 1892 | Berti (1989a, 1989b, 1990a), Silfverberg (1972) |
| *Lanolepta* Kimoto, 1991 | 1 | 0 | 0 | PAR (China: Taiwan) |  | Kimoto (1991a) |
| *Laosixantha* Kimoto, 1989 | 2 | 0 | 0 | ORR |  | Bezděk (2012b), Kimoto (1989a), Medvedev (2007c, 2009a) |
| *Laphris* Baly, 1864 | 7 | 1 | 0 | ORR (Vietnam), PAR | ^*^*Neohylaspes* Chûjô, 1962 | Kimoto (1989a), Lee and Beenen (2009), Yang (1992c, 1992e, 1993d), Yang and Li (2004) |
| *Leptarthra* Baly, 1861 | 6 | 0 | 0 | ORR, PAR |  | Bezděk (2012b), Kimoto (1979), Lee and Bezděk (2013a), Vazirani (1970) |
| *Leptarthroides* Beenen, 2009 | 1 | 0 | 0 | ORR (Myanmar) |  | Beenen (2009) |
| *Leptaulaca* Weise, 1902 | 15 | 0 | 0 | AFR, PAR (Yemen) |  | Berti (1990a, 1990b) |
| *Leptomona* Bechyné, 1958 | 4 | 0 | 0 | PAR |  | Bieńkowski and Orlova-Bienkowskaja (2013), Schlich and Wagner (2010) |
| *Leptonesiotes* Blake, 1958 | 3 | 0 | 0 | NTR (Cuba) |  |  |
| *Leptoxena* Baly, 1888 | 1 | 0 | 0 | ORR (Andaman Islands) |  |  |
| *Lesnella* Laboissière, 1932 | 1 | 0 | 0 | AFR (Mozambique) |  |  |
| *Lilophaea* Bechyné, 1958 | 23 | 0 | 0 | NTR |  | Bechyné (1997), Bechyné and Springlová-Bechyné (1970), Groll and Moura (2016) |
| *Liroegala* Medvedev, 2015 | 1 | 0 | 0 | PAR (China: Yunnan) |  | Medvedev (2015b) |
| *Liroetina* Medvedev, 2013 | 2 | 0 | 0 | ORR |  | Medvedev (2013d) |
| *Liroetis* Weise, 1889 | 34 | 1 | 0 | ORR, PAR | ^∆^*Liroetinus* Lopatin, 2004 | Bezděk (2016d), Bezděk, Romantsov and Medvedev (2014), Chen and Jiang (1986b), Chûjô (1966), Hartmann and Medvedev (2003), Jiang (1988b), Kimoto (1989a), Lopatin (2004a, 2004b, 2013), Medvedev (2004b, 2007b, 2009f, 2011a), Takizawa (1988a), Warchałowski (2008), Zhang, Li and Yang (2008c) |
| *Liroetoides* Kimoto, 1989 | 2 | 0 | 0 | ORR |  | Bezděk (2013b), Kimoto (1989a) |
| *Lochmaea* Weise, 1883 | 12 | 0 | 0 | PAR | ^*^*Lochmaeata* Strand, 1935 | Aslam (1972), Beenen (1996, 2010b), Bezděk (2004a), Gök et al (2006), Kimoto (1979, 1996), Medvedev (2005b), Silfverberg (1974), Takizawa (1990), Yang, Wang and Wu (1998) |
| *Lomirana* Laboissière, 1932 | 6 | 0 | 0 | AUR (New Guinea), ORR |  | Kimoto (1989a) |
| *Luisiadia* Medvedev, 1979 | 1 | 0 | 0 | AUR (Louisiada Archipelago) |  | Medvedev (1979) |
| *Luperacantha* Laboissière, 1932 | 2 | 0 | 0 | AFR (Madagscar) |  |  |
| *Luperocella* Jacoby, 1900 | 3 | 0 | 0 | ORR | ^*^*Alafia* Maulik, 1936 | Aslam (1972), Medvedev (2001d) |
| *Luperocida* Medvedev and Dang, 1981 | 1 | 0 | 0 | ORR (Vietnam) |  | Medvedev and Dang (1981) |
| *Luperodes* Motschulsky, 1858 | 77 | 0 | 1 | AFR, AUR, ORR, NTR |  | Bezděk (2012b), Bauer and Wagner (2010), Bolz and Wagner (2005), Hazmi and Wagner (2013), Kimoto (1989a), Medvedev (2007d), Mertgen and Wagner (2006), Wagner and Bieneck (2012), Wagner and Kurtscheid (2005), Wilcox (1975) |
| *Luperogala* Medvedev and Samoderzhenkov, 1989 | 4 | 0 | 0 | ORR |  | Bezděk (2012b), Bezděk, Romantsov and Medvedev (2014), Medvedev (2004b), Medvedev and Samoderzhenkov (1989), Warchałowski (2008) |
| *Luperolophus* Fairmaire, 1876 | 3 | 0 | 0 | AFR (Madagascar) |  |  |
| *Luperosoma* Jacoby, 1891 | 13 | 0 | 0 | NAR, NTR | ^*^*Deuterobrotica* Bechyné, 1958 |  |
| *Luperososia* Laboissière, 1935 | 3 | 0 | 0 | AFR |  | Laboissière (1940) |
| *Luperus* Geoffroy, 1762 | 97 | 1 | 1 | AFR, AUR (New Guinea), ORR, PAR, NAR, NTR |  | Aslam (1972), Bechyné and Springlová-Bechyné (1967), Beenen (2008a, 2010b, 2010c, 2011b, 2016d), Bezděk (2006d, 2007a, 2015a), Bieńkowski (1997), Bolz and Wagner (2005), Fogato (1979), Iablokoff-Khnzorian (1970), ICZN (1984), Kimoto (1983, 1989a, 2004), Lee et al. (2016), Lopatin (1975, 1983, 1984, 1990, 2005c), Medvedev (1975, 1978, 1998a, 2007d, 2011b, 2012c), Mohamedsaid (1999d), Pic (1903), Romantsov (2004), Schlechtendal (1894), Silfverberg (1979), Tomov (1975), Vela and Bastazo (1990), Warchałowski (1991) |
| *Lusingania* Laboissière, 1919 | 4 | 0 | 0 | AFR |  | Aslam (1972), Bryant (1958) |
| *Lygistus* Wilcox, 1965 | 1 | 0 | 0 | NAR (Arizona) |  |  |
| *Macrima* Baly, 1878 | 12 | 0 | 0 | PAR | ^*^*Sepharia* Fairmaire, 1889; ^*^*Glechonis* Weise, 1889 | Jiang (1990b), Kimoto (1989a), Medvedev (1995c, 2009e, 2011c), Mohamedsaid (1999c, 1999d, 2000c), Yang (1992b) |
| *Madurasia* Jacoby, 1896 | 2 | 0 | 0 | AFR, ORR, PAR | ^*^*Neorudolphia* Laboissière, 1926 | Aslam (1972), Prathapan (2016), Wagner and Bieneck (2012) |
| *Maevatanania* Bechyné, 1964 | 1 | 0 | 0 | AFR (Madagascar) |  |  |
| *Mahutia* Laboissière, 1917 | 10 | 0 | 0 | AFR |  | Beenen (2010a, 2015, 2016), Silfverberg (1973a, 1980) |
| *Malaconida* Fairmaire, 1886 | 4 | 0 | 0 | AFR (Madagascar) | ^*^*Otacilus* Jacoby, 1888; ^*^*Adialyta* Fairmaire, 1902 |  |
| *Malacorhinus* Jacoby, 1887 | 23 | 0 | 0 | NAR, NTR |  | Mignot (1970) |
| *Malacotheria* Fairmaire, 1881 | 6 | 0 | 0 | AUR (Fiji, New Caledonia, New Britain) |  | Beenen (2008b, 2013a) |
| *Marmina* Shute, 1983 | 2 | 0 | 0 | AUR (New Guinea) |  | Shute (1983) |
| *Marseulia* Joannis, 1865 | 1 | 0 | 0 | PAR |  |  |
| *Masurius* Jacoby, 1888 | 1 | 0 | 0 | NTR (Panama) |  |  |
| *Medythia* Jacoby, 1887 | 19 | 0 | 0 | AFR, ORR, PAR | ^*^*Paraluperodes* Ogloblin, 1936 | Aslam (1972), Berti (1983), Bryant (1959a), Medvedev (2012d), Kimoto (1981a, 1989a), Medvedev (2007c), Mohamedsaid (1997f, 1999a), Wagner and Bieneck (2012), Wilcox (1975) |
| *Megaleruca* Laboissière, 1922 | 17 | 0 | 0 | AFR |  |  |
| *Megalognatha* Baly, 1878 | 93 | 1 | 0 | AFR |  | Aslam (1972), Bryant (1958), Grobbelaar (1993, 1995), Weise (1926) |
| *Mellesia* Weise, 1902 | 4 | 0 | 0 | AFR | ^*^*Mellesianella* Laboissière, 1922 | Aslam (1972) |
| *Menippus* Clark, 1864 | 28 | 0 | 0 | AUR, ORR, PAR | ^*^*Issikia* Chûjô, 1961 | Aslam (1972), Kimoto (1979, 1989a), Kimoto and Takizawa (1981), Lee, Bezděk and Suenaga (2012), Medvedev (2001d, 2013b), Mohamedsaid (1999g), Reid and Nally (2008) |
| *Meristata* Strand, 1935 | 9 | 4 | 0 | ORR, PAR | ^*^*Merista* Chapuis, 1875 | Bezděk (2012b), Medvedev (1999), Silfverberg (1990), Vazirani (1970), Zhang and Yang (2002) |
| *Meristoides* Laboissière, 1929 | 6 | 0 | 0 | ORR, PAR | ^∆^*Sichuanella* Lopatin, 2008; ^*^*Sichuania* Lopatin, 2002 | Lee and Bezděk (2014b), Lopatin (2002b, 2008a, 2011b) |
| *Metacoryna* Jacoby, 1888 | 7 | 0 | 0 | NTR | ^*^*Cyphotarsis* Jacoby, 1892 | Clark (1998) |
| *Metacycla* Baly, 1861 | 7 | 0 | 0 | NTR | ^*^*Gastrogyna* Leconte, 1865 | Beenen (2013b) |
| *Metalepta* Baly, 1861 | 3 | 0 | 0 | NTR |  | Beenen (2013b) |
| *Metopoedema* Duvivier, 1891 | 2 | 0 | 0 | AFR (Madagascar) |  |  |
| *Metrioidea* Fairmaire, 1881 | 39 | 2 | 0 | AUR, NAR, NTR, ORR |  | Beenen (2008b, 2013a), Mohamedsaid (1994b, 1997c, 2004) |
| *Metrobrotica* Bechyné, 1958 | 1 | 0 | 0 | NTR |  |  |
| *Metrogaleruca* Bechyné and Bechyné, 1969 | 4 | 0 | 0 | NTR |  | Moura (2016) |
| *Microbrotica* Jacoby, 1887 | 1 | 0 | 0 | NTR (Panama) |  |  |
| *Microexosoma* Laboissière, 1931 | 2 | 0 | 0 | AFR |  | Aslam (1972) |
| *Microlepta* Jacoby, 1886 | 8 | 0 | 0 | AUR, ORR |  | Bezděk (2013a), Mohamedsaid (1997c, 2001e) |
| *Microscelida* Clark, 1998 | 11 | 0 | 0 | NTR (Mexico) |  | Clark (1998) |
| *Miltina* Chapuis, 1875 | 1 | 0 | 0 | ORR, PAR |  |  |
| *Mimagitocera* Maulik, 1936 | 1 | 0 | 0 | ORR, PAR (Nepal) |  |  |
| *Mimastra* Baly, 1865 | 73 | 0 | 0 | ORR, PAR | ^*^*Anthraxantha* Fairmaire, 1878; *Brachita* Allard, 1889 | Bezděk (2007c, 2009c, 2010c, 2011, 2013c), Bezděk and Lee (2011), Hartmann and Medvedev (2003), Kimoto (1970a, 1982a, 1989a), Kimoto and Takizawa (1972, 1983), Lopatin (2004b), Medvedev (1995, 2000d, 2002a, 2009c, 2011c, 2013c, 2015d), Mohamedsaid (1992), Takizawa (1986a, 1990), Zhang et al (2006) |
| *Mimastracella* Jacoby, 1903 | 12 | 0 | 0 | ORR, PAR | ^*^ *Eriosarda* Jacoby, 1903 | Bezděk (2007c, 2012b), Kimoto (1984, 1989a), Lopatin (2002c), Medvedev (1972), Samoderzhenkov (1988), Takizawa (1978, 1988a), Yu, Wang and Yang (1996) |
| *Mimastroides* Jacoby, 1892 | 2 | 0 | 0 | AFR (Madagascar) |  |  |
| *Mimastrosoma* Medvedev, 2004 | 1 | 0 | 0 | ORR (Vietnam) |  | Medvedev (2004b) |
| *Mindana* Allard, 1889 | 8 | 0 | 0 | ORR (Philippines) |  | Medvedev (1995) |
| *Mindella* Medvedev, 1995 | 2 | 0 | 0 | ORR (Philippines) |  | Medvedev (1995) |
| *Mindorina* Laboissière, 1940 | 4 | 0 | 0 | ORR (Philippines) |  |  |
| *Miraces* Jacoby, 1888 | 6 | 0 | 0 | NAR, NTR | ^*^*Halticidea* Horn, 1893 |  |
| *Momaea* Baly, 1865 | 8 | 0 | 0 | AUR, ORR |  | Aslam (1972), Mohamedsaid (1999c), Shute (1983) |
| *Mombasa* Fairmaire, 1884 | 3 | 0 | 0 | AFR | ^*^*Mombasica* Fairmaire, 1887 |  |
| *Monardia* Laboissière, 1939 | 2 | 0 | 0 | AFR |  | Bolz and Wagner (2012) |
| *Monocesta* Clark, 1865 | 29 | 2 | 0 | NTR |  |  |
| *Monocestoides* Duvivier, 1891 | 3 | 0 | 0 | AFR (Madagascar) |  |  |
| *Monocida* Jacoby, 1899 | 7 | 0 | 0 | AFR |  |  |
| *Monolepta* Chevrolat, 1836 | 708 | 6 | 0 | AFR, AUR, NTR, ORR, PAR | ^*^*Damais* Jacoby, 1903; ^*^*Aemulaphthona* Scherer, 1969; ^*^*Chimporia* Laboissière, 1931 | Aslam (1972), Basu (1996), Beenen (2008a, 2008b, 2010b, 2010c, 2013a), Bezděk (1998, 2007a, 2009a, 2012c, 2013a), Bieńkowski and Orlova-Bienkowskaja (2013), Bolz and Wagner (2005, 2012, 2014c), Chen et al. (1976), Esch and Wagner (2009), Freund and Wagner (2003), Gök, Aslan and Aslan (2005), Grobbelaar (2008), Hasenkamp and Wagner (2000), Hazmi and Wagner (2010a, 2013), Heunemann et al (2015), Kimoto (1974b, 1977, 1982a, 1989a, 1996, 2004, 2005), Kimoto and Takizawa (1983), Konstantinov and Lingafelter (2002), Kortenhaus and Wagner (2010), Lee (2009, 2016b), Lee and Beenen (2012), Lee and Staines (2010), Lee, Tian and Staines (2010), Li and Wang (1993), Lopatin (2003b), Medvedev (1972, 1979, 1985, 1990, 1992b, 1996, 1997a, 1997b, 1998b, 1999, 2000b, 2000c, 2000d, 2002d, 2005b, 2005c, 2007b, 2007c, 2007d, 2009a, 2009d, 2012b, 2012e, 2013d, 2014a, 2015a, 2015b), Medvedev and Javorskaya (2008), Medvedev and Sprecher-Uebersax (1998), Mertgen and Wagner (2006), Middelhauve and Wagner (2001), Mohamedsaid (1993b, 1997c, 1997d, 1997f, 1998g, 1998h, 1999f, 2000a, 2000b, 2000c, 2001c, 2001e, 2002a, 2005), Mohamedsaid and Constant (2007), Mohamedsaid and Kimoto (1993), Prathapan (2016), Radford (1981), Rizki et al (2014), Scherer (1969), Schlich and Wagner (2010), Schmitz and Wagner (2001), Stapel and Wagner (2000, 2001), Steiner and Wagner (2005), Takizawa (1986b, 1988b), Takizawa and Kimoto (1990), Wagner (2000a, 2000b, 2001a, 2001b, 2002, 2003, 2005, 2007a, 2007b, 2007c, 2011, 2016), Wagner and Bieneck (2012), Wagner and Freund (2003), Wagner and Kurtscheid (2005), Wagner and Scherz (2002), Warchałowski (2004), Yang and Gan (1993) |
| *Monoleptinia* Laboissière, 1932 | 4 | 0 | 0 | AFR (Madagascar) |  |  |
| *Monoleptocrania* Laboissière, 1940 | 1 | 0 | 0 | AFR |  | Stapel and Wagner (2000) |
| *Monoleptoides* Wagner, 2011 | 9 | 0 | 0 | AFR |  | Wagner (2011) |
| *Monoxia* Leconte, 1865 | 18 | 0 | 0 | NAR, NTR |  | Clark, Rattu and Cillo (2014) |
| *Morokasia J*acoby, 1904 | 1 | 0 | 0 | AUR (New Guinea) |  |  |
| *Morphosphaera* Baly, 1861 | 12 | 0 | 0 | ORR, PAR |  | Chen et al. (1976), Kimoto (1989a), Lee and Bezděk (20016b) |
| *Morphosphaeroides* Jacoby, 1903 | 1 | 0 | 0 | AFR |  |  |
| *Munina* Chen, 1976 | 2 | 0 | 0 | ORR, PAR |  | Bezděk (2009b), Chen et al. (1976), Yang, Li and Yao (1997) |
| *Nadrana* Baly, 1865 | 9 | 0 | 0 | ORR | ^*^*Batusia* Jacoby, 1897 | Kimoto (1989a), Medvedev (2016), Mohamedsaid (1998c, 1998h, 2000a, 2001e), Zulfadli, Izfa and Alia (2015) |
| *Nancita* Allard, 1889 | 1 | 0 | 0 | ORR (Phillippines) |  |  |
| *Narichona* Kirsch, 1883 | 3 | 0 | 0 | NTR |  |  |
| *Neoatysa* Abdullah and Qureshi, 1968 | 1 | 0 | 0 | PAR (Pakistan) |  | Abdullah and Qureshi (1968a) |
| *Neobarombiella* Bolz and Wagner, 2012 | 36 | 0 | 0 | AFR, PAR (Socotra) |  | Bolz and Wagner (2012, 2014a), Bryant (1958) |
| *Neobrotica* Jacoby, 1887 | 63 | 1 | 0 | NTR |  | Bechyné and Springlová-Bechyné (1970) |
| *Neodrana* Jacoby, 1886 | 4 | 0 | 0 | AUR (New Guinea) |  |  |
| *Neolepta* Jacoby, 1884 | 8 | 0 | 0 | AUR (New Guinea), ORR |  | Hazmi and Wagner (2010b, 2013), Kimoto (1989a), Mohamedsaid (1997d, 2001e) |
| *Neolochmaea* Laboissière, 1939 | 4 | 0 | 0 | NTR |  | LeSage (1986), Moura (1998a, 1998b, 1998c), White (1979) |
| *Neomahutia* Laboissière, 1936 | 1 | 0 | 0 | AFR (RSA) |  |  |
| *Neophaestus* Hincks, 1949 | 1 | 0 | 0 | NTR (Panama) | ^*^*Phaestus* Jacoby, 1887 |  |
| *Neorupilia* Blackburn, 1888 | 6 | 0 | 0 | AUR |  |  |
| *Neosastra* Abdullah and Qureshi, 1968 | 1 | 0 | 0 | PAR (Pakistan) |  | Abdullah and Qureshi (1968a) |
| *Nepalogaleruca* Kimoto, 1970 | 7 | 0 | 0 | PAR (Nepal, Xizang) |  | Chen and Jiang (1987a), Kimoto (1970a), Kimoto and Takizawa (1972), Medvedev (1990, 2003b), Medvedev and Sprecher-Uebersax (1997), Silfverberg (1990) |
| *Nepalolepta* Medvedev, 1992 | 1 | 0 | 0 | PAR (Nepal) |  | Medvedev (1992b) |
| *Nestinus* Clark, 1865 | 8 | 0 | 0 | NTR (Mexico, Guatemala) |  |  |
| *Niasia* Jacoby, 1889 | 3 | 0 | 0 | ORR |  | Reid (1998a) |
| *Nirina* Weise, 1892 | 5 | 0 | 0 | AFR |  | Aslam (1972) |
| *Nirinoides* Jacoby, 1903 | 4 | 1 | 0 | AFR |  |  |
| *Notonicea* Hincks, 1949 | 7 | 0 | 0 | AUR (New Guinea) | ^*^*Nicea* Baly, 1865 | Aslam (1972), Bezděk (2012b) |
| *Nototrichaspis* Hincks, 1949 | 2 | 0 | 0 | AUR | ^*^*Trichaspis* Weise, 1923 |  |
| *Nyctidromas* Semenov, 1896 | 4 | 1 | 0 | PAR | ^*^*Nyctiphantus* Semenov, 1902 | Beenen (2010b), Medvedev (2005b) |
| *Nyctiplanctus* Blake, 1963 | 8 | 0 | 0 | NTR (Carribean Islands) |  | Clark, Lillrose and Belo Neto (2013) |
| *Nymphius* Weise, 1900 | 11 | 2 | 0 | PAR |  | Beenen (2010b), Bezděk (2007a, 2008c, 2012b), Fogato (1981), Lopatin (2002a, 2006c), Medvedev (1996) |
| *Ochralea* Clark, 1865 | 2 | 0 | 0 | ORR |  | Hazmi and Wagner (2010a), Mohamedsaid (2005) |
| *Oides* Weber, 1801 | 162 | 3 | 0 | AFR, AUR, PAR, ORR | ^*^*Adorium* Fabricius, 1801; ^*^*Boisduvalia* Montrouzier, 1856; ^*^*Botanoctona* Fairmaire, 1877; ^*^*Callipepla* Dejean, 1836; ^*^*Isosoma* Billberg, 1820; ^*^*Ochralea* Chevrolat, 1836; ^*^*Rhombopalpa* Chevrolat, 1836 | Aslam (1972), Beenen (2004, 2007b), Berti (1986a, 1987, 1993), Chen and Jiang (1981), Kimoto (1989a), Medvedev (2015a), Samoderzhenkov (1992), Vachon (1977, 1980a, 1980b) |
| *Oidomorpha* Laboissière, 1924 | 1 | 0 | 0 | AFR (Kenya) |  |  |
| *Oorlogia* Silfverberg, 1978 | 1 | 0 | 0 | AFR (Namibia) |  | Silfverberg (1978b) |
| *Oosagitta* Kortenhaus and Wagner, 2013 | 6 | 0 | 0 | AFR |  | Kortenhaus and Wagner (2013) |
| *Ootheca* Dejean, 1836 | 26 | 0 | 0 | AFR | ^*^*Ergana* Chapuis, 1879 | Aslam (1972), Bolz and Wagner (2012), Grobbelaar (2008), Kortenhaus and Wagner (2010, 2012, 2013) |
| *Oothecoides* Kortenhaus and Wagner, 2011 | 6 | 0 | 0 | AFR |  | Kortenhaus and Wagner (2011) |
| *Ootibia* Kortenhaus and Wagner, 2012 | 5 | 0 | 0 | AFR |  | Kortenhaus and Wagner (2012) |
| *Ophraea* Jacoby, 1886 | 12 | 0 | 0 | NAR, NTR |  |  |
| *Ophraella* Wilcox, 1965 | 17 | 0 | 0 | NAR, NTR |  | Bechyné (1997), Futuyuma (1990, 1991), LeSage (1986), Moura (2016), White (1979) |
| *Ornithognathus* Thomson, 1858 | 6 | 0 | 0 | AFR |  |  |
| *Oroetes* Jacoby, 1888 | 2 | 0 | 0 | NTR |  |  |
| *Orthoneolepta* Hazmi and Wagner, 2013 | 2 | 0 | 0 | ORR |  | Hazmi and Wagner (2013), Mohamedsaid (1997d) |
| *Orthoxia* Clark, 1865 | 1 | 0 | 0 | ORR |  |  |
| *Orthoxioides* Laboissière, 1922 | 3 | 0 | 0 | AFR |  |  |
| *Pachytomellina* Hincks, 1949 | 4 | 0 | 0 | AFR | ^*^*Pachytomella* Laboissière, 1922 |  |
| *Palaeophylia* Jacoby, 1903 | 9 | 0 | 0 | AFR |  |  |
| *Palaeosastra* Jacoby, 1906 | 1 | 0 | 0 | AUR (New Guinea) |  | Bezděk (2009a) |
| *Paleosepharia* Laboissière, 1936 | 76 | 1 | 0 | ORR, PAR |  | Beenen (2008a, 2010b), Bezděk (2009a), Chen and Jiang (1984, 1986b), Jiang (1990b), Kimoto (1982a, 1989a), Kimoto and Takizawa (1983), Medvedev (2001b, 2004a, 2004b, 2007b, 2009a, 2012b, 2013d, 2014b), Medvedev and Sprecher-Uebersax (1998), Mohamedsaid (1996a, 1997c, 1998h, 1999c, 2000a, 2000c, 2001e), Mohamedsaid and Constant (2007), Rizki et al (2014, 2016), Takizawa and Basu (1987), Wagner and Bieneck (2012), Wang and Yang (1998), Yang (2002) |
| *Pallasiola* Jacobson, 1925 | 1 | 0 | 0 | PAR | ^*^*Pallasia* Weise, 1886 | Beenen (2008a, 2010b, 2014c), Mandl (1970, 1976, 1981) |
| *Palmaria* Bechyné, 1956 | 1 | 0 | 0 | NTR |  |  |
| *Palpaenidea* Laboissière, 1933 | 3 | 0 | 0 | AUR (New Guinea), ORR |  | Mohamedsaid (1997c, 1999d) |
| *Palpoxena* Baly, 1861 | 55 | 0 | 0 | AFR, ORR, PAR | ^*^*Aenidea* Baly 1874; ^*^*Paraenidea* Laboissière, 1933 | Bezděk (2012b), Bryant (1955, 1958), Jacoby (1879), Kimoto (1989a, 1990, 2003), Kizub (2016), Medvedev (1972, 2001d, 2015b), Mohamedsaid (1996b, 1997a, 1997c, 1999d), Reid (1999) |
| *Panafrolepta* Mertgen and Wagner, 2006 | 1 | 0 | 0 | AFR |  | Mertgen and Wagner (2006) |
| *Papuania* Jacoby, 1906 | 1 | 0 | 0 | AUR (New Guinea) |  |  |
| *Parabrotica* Bechyné and Bechyné, 1961 | 3 | 0 | 0 | NTR | SYN: *Neotrichota* Blake, 1966 |  |
| *Paraclitena* Medvedev, 1972 | 1 | 0 | 0 | ORR (Sri Lanka) |  | Medvedev (1972) |
| *Parageina* Laboissière, 1936 | 2 | 0 | 0 | ORR |  |  |
| *Paragetocera* Laboissière, 1929 | 11 | 1 | 0 | PAR |  | Jiang (1992), Zhang and Yang (2004) |
| *Paranapiacaba* Bechyné, 1958 | 58 | 2 | 0 | NTR |  | Bechyné (1997), Blake (1971), Cabrera (1994), Derunkov and Konstantinov (2013), Moura (2003, 2009) |
| *Paraneolepta* Hazmi and Wagner, 2013 | 3 | 0 | 0 | ORR |  | Hazmi and Wagner (2013) |
| *Paranoides* Vachon, 1976 | 2 | 0 | 0 | AUR (New Guinea) |  | Vachon (1976) |
| *Parantongila* Laboissière, 1932 | 1 | 0 | 0 | AFR (Madagascar) |  |  |
| *Paraplotes* Laboissière, 1933 | 20 | 0 | 0 | ORR, PAR |  | Beenen (2011c), Chen and Jiang (1986a), Jiang (1989), Lee (2015), Medvedev (2002b, 2008b, 2015b), Medvedev and Sprecher-Uebersax (1998), Takizawa and Basu (1987), Wu, Yang and Li (1998), Zhang, Li and Yang (2008b) |
| *Parapophylia* Laboissière, 1922 | 2 | 0 | 0 | AFR |  |  |
| *Parasbecesta* Laboissière, 1940 | 10 | 0 | 0 | AFR |  | Aslam (1972) |
| *Paraspitiella* Chen and Jiang, 1981 | 2 | 0 | 0 | PAR (Xizang) |  | Chen and Jiang (1981), Yang and Li (2004) |
| *Parastetha* Baly, 1879 | 1 | 0 | 0 | ORR (Assam) |  |  |
| *Paratriarius* Schaeffer, 1906 | 52 | 0 | 0 | NAR, NTR | ^*^*Chanchamayia* Bechyne, 1956 | Bechyné and Springlová-Bechyné (1967) |
| *Paraxenoda* Mohamedsaid, 1999 | 1 | 0 | 0 | ORR (Borneo) |  | Mohamedsaid (1999c) |
| *Parexosoma* Laboissière, 1932 | 9 | 1 | 0 | ORR, PAR | ^*^*Bijukta* Maulik, 1936 | Beenen (2009), Chûjô (1966), Döberl (1995), Jiang (1990b), Mohamedsaid (2001d) |
| *Paridea* Baly, 1886 | 87 | 1 | 0 | ORR, PAR | ^#^*Semacia* Fairmaire, 1889. ^*^*Aeropa* Weise, 1889; ^*^*Carapaula* Chûjô, 1962; ^*^*Paraulaca* Baly, 1888; ^*^*Semacianella* Laboissière, 1930 | Chen and Jiang (1981, 1987b), Kimoto (1970a, 1974a, 1977, 1989a, 2004), Lee and Bezděk (2014a), Medvedev (2004b, 2005b), Medvedev and Samoderzhenkov (1989, 1998), Medvedev and Sprecher-Uebersax (1998), Mohamedsaid (1998i), Takizawa (1990), Yang (1991a, 1992e, 1993c) |
| *Paumomua* Jacoby, 1904 | 1 | 0 | 0 | AUR (New Guinea) |  |  |
| *Periclitena* Weise, 1902 | 4 | 0 | 0 | ORR, PAR |  | Kimoto (1989a), Samoderzhenkov (1988) |
| *Philastra* Medvedev, 1995 | 1 | 0 | 0 | ORR (Philippines) |  | Medvedev (1995) |
| *Phyllecthris* Dejean, 1836 | 3 | 0 | 0 | NAR |  |  |
| *Phyllobrotica* Chevrolat, 1836 | 28 | 2 | 0 | PAR, ORR, NAR |  | Bezděk (2002, 2010a), Gilbert (2008), Hatch (1971), Lee and Bezděk (2015), Riley (1979), Takizawa (1985b), Warchałowski (1998) |
| *Phyllobroticella* Jacoby, 1894 | 12 | 0 | 0 | AFR |  | Beenen (2016) |
| *Phyllocleptis* Weise, 1913 | 6 | 0 | 0 | AUR (New Guinea), ORR |  |  |
| *Pimentelia* Laboissière, 1932 | 4 | 1 | 0 | AFR |  | Bolz and Wagner (2014b) |
| *Plagiasma* Weise, 1903 | 1 | 0 | 0 | AFR (Cameroon) |  |  |
| *Platybrotica* Cabrera and Cabrera Walsh, 2004 | 1 | 0 | 0 | NTR |  | Cabrera and Cabrera Walsh (2004a) |
| *Platymorpha* Jacoby, 1888 | 4 | 0 | 0 | NTR |  |  |
| *Platynocera* Blanchard, 1846 | 3 | 0 | 0 | NTR | ^*^*Corynocesta* Bechyné, 1956 |  |
| *Pleronexis* Weise, 1908 | 2 | 0 | 0 | AUR | ^*^*Plesistia* Maulik, 1929 | Beenen (2008b) |
| *Polexima* Weise, 1903 | 31 | 0 | 0 | AFR, ORR | ^*^*Haplotes* Weise, 1903; ^*^*Platyhaplotes* Hincks, 1949 | Beenen (2011b), Berti (1990b), Mohamedsaid (1999c, 2004) |
| *Polysastra* Shute, 1983 | 22 | 1 | 0 | AUR |  | Shute (1983) |
| *Poneridia* Weise, 1908 | 9 | 0 | 0 | AUR, ORR |  | Aslam (1972), Medvedev (2013f), Reid (2001b) |
| *Porechontes* Blake, 1966 | 3 | 0 | 0 | NTR |  |  |
| *Porphytoma* Jacoby, 1903 | 1 | 0 | 0 | AFR |  |  |
| *Potamobrotica* Blake, 1966 | 3 | 0 | 0 | NTR |  |  |
| *Prasyptera* Baly, 1878 | 24 | 0 | 0 | AUR, ORR |  | Allard (1890) |
| *Priapina* Jacoby, 1887 | 1 | 0 | 0 | ORR (Sri Lanka) |  | Wagner and Bieneck (2012) |
| *Proegmena* Weise, 1889 | 5 | 0 | 0 | PAR |  | Chûjô (1966), Kimoto (1996), Takizawa (1978) |
| *Prosmidia* Weise, 1901 | 19 | 3 | 0 | AFR | ^*^*Idacanthina* Silfverberg, 1973; ^*^*Megalaetana* Silfverberg, 1973; ^*^*Paracanthina* Hincks, 1949; ^*^*Trichacanthina* Silfverberg, 1982; ^*^*Neolaetana* Laboissière, 1921. ^*^*Paracantha* Laboissière, 1921 | Silfverberg (1972, 1973b, 1975b, 1975c, 1982b) |
| *Protocoelocrania* Laboissière, 1931 | 2 | 0 | 0 | AFR |  | Berti (1970b) |
| *Protoleptonyx* Laboissière, 1932 | 1 | 0 | 0 | AFR (Madagascar) |  |  |
| *Pseudadimonia* Duvivier, 1891 | 15 | 0 | 0 | ORR, PAR |  | Beenen (2013b), Jiang (1991), Mandl (1974, 1986), Medvedev (2015c), Medvedev and Sprecher-Uebersax (1997), Samoderzhenkov (1988) |
| *Pseudaenidea* Laboissière, 1938 | 3 | 0 | 0 | AFR |  |  |
| *Pseudaplosonyx* Duvivier, 1884 | 1 | 0 | 0 | ORR |  |  |
| *Pseudapophylia* Jacoby, 1903 | 1 | 0 | 0 | AFR |  |  |
| *Pseudespera* Chen, Wang and Jiang, 1985 | 6 | 0 | 0 | PAR |  | Beenen (2008a), Chen, Wang and Jiang (1985), Jiang (1992) |
| *Pseudeustetha* Jacoby, 1899 | 4 | 0 | 0 | ORR | ^*^*Avinasa* Maulik, 1936 | Aslam (1972), Kimoto (1989a), Medvedev (2002d), Mohamedsaid (1995c) |
| *Pseudikopista* Laboissière, 1932 | 1 | 0 | 0 | AFR (Madagscar) |  |  |
| *Pseudocneorane* Medvedev and Romantsov, 2012 | 1 | 0 | 0 | ORR (Thailand) |  | Medvedev and Romantsov (2012) |
| *Pseudocophora* Jacoby, 1884 | 27 | 1 | 0 | ORR, PAR |  | Chen et al. (1976), Kimoto (1989a), Samoderzhenkov (1992), Yang (1991c) |
| *Pseudodiabrotica* Jacoby, 1892 | 1 | 0 | 0 | NTR (Mexico) |  |  |
| *Pseudoides* Jacoby, 1892 | 5 | 0 | 0 | ORR |  | Beenen (2008a), Chûjô (1966), Kimoto (1989a, 2000a, 2000b, 2001), Medvedev (2007d), Wagner and Bieneck (2012) |
| *Pseudolognatha* Jacoby, 1903 | 3 | 0 | 0 | AFR |  | Aslam (1972), Bryant (1955) |
| *Pseudoluperus* Beller and Hatch, 1932 | 15 | 0 | 0 | NAR, NTR |  | Andrews and Gilbert (2005), Clark (1996, 1998), Riley, Clark and Gilbert (2001) |
| *Pseudomalaxia* Laboissière, 1926 | 1 | 0 | 0 | AFR |  |  |
| *Pseudorupilia* Jacoby, 1893 | 9 | 0 | 0 | AFR | ^*^*Rhabdocneorane* Laboissière, 1935 | Beenen (2016), Grobbelaar (1995), Silfverberg (1969, 1973a) |
| *Pseudosastra* Jacoby, 1904 | 7 | 0 | 0 | ORR |  | Aslam (1972), Mohamedsaid (1993c) |
| *Pseudoscelida* Jacoby, 1894 | 5 | 0 | 0 | ORR |  | Medvedev (2001b), Mohamedsaid (2001e) |
| *Pseudosepharia* Laboissière, 1936 | 4 | 0 | 0 | PAR |  | Beenen (2010b), Jiang (1990a, 1992) |
| *Pseudoshaira* Beenen, 2007 | 2 | 0 | 0 | ORR (Borneo) |  | Beenen (2007a, 2013b) |
| *Pteleon* Jacoby, 1888 | 3 | 0 | 0 | NAR, NTR |  | Riley, Clark and Gilbert (2001) |
| *Pterophthinus* Gressitt and Kimoto, 1963 | 1 | 0 | 0 | PAR |  |  |
| *Pubibrotica* Medvedev, 2002 | 1 | 0 | 0 | ORR (India) |  | Medvedev (2002b) |
| *Pyesia* Clark, 1865 | 14 | 4 | 0 | NTR |  | Bechyne and Springlova de Bechyne (1961, 1970) |
| *Pyrrhalta* Joannis, 1865 | 84 | 0 | 0 | ORR, PAR | ^*^*Decoomanius* Laboissière, 1927; ^*^*Chapalia* Laboissière, 1929 | Beenen (2003b, 2008a, 2010b), Bezděk (2003a, 2007c), Chen and Jiang (1981), Kimoto (1974a, 1976, 1977, 1979, 1981b, 1984, 1989a, 1994, 1996, 2001), Kimoto and Takizawa (1981), Lopatin (2002b, 2005b), Medvedev (1995, 2000b, 2002b, 2005a, 2010, 2012b, 2012e, 2013f, 2014a), Medvedev and Sprecher-Uebersax (1999), Mohamedsaid (1995c, 1997f, 1999c), Nie et al (2013), Reid (2001b), Riley, Clark and Seeno (2003), Samoderzhenkov (1988), Silfverberg (1974), Skomorokhov (2011), Sprecher-Uebersax and Zoia (2002), Takizawa (1990), Xue and Yang (2010), Yang (1992e) |
| *Radymna* Reitter, 1913 | 8 | 0 | 0 | PAR | ^*^*Prophyllis* Reitter, 1913; ^*^*Galerupipla* Maulik, 1936 | Aslam (1972), Beenen (2008a, 2010b, 2014c) |
| *Rachicephala* Blake, 1966 | 1 | 0 | 0 | NTR (Mexico) |  |  |
| *Rhabdotilla* Jacobson, 1911 | 1 | 0 | 0 | distr. unknown |  | Mandl (1986), Beenen (2008a) |
| *Rohania* Laboissière, 1921 | 11 | 0 | 0 | AFR |  | Beenen (2011b), Bryant (1959b) |
| *Rohaniella* Laboissière, 1940 | 2 | 0 | 0 | AFR |  |  |
| *Romanita* Bechyné, 1958 | 5 | 0 | 0 | NTR |  |  |
| *Rubrarcastes* Hazmi and Wagner, 2010 | 1 | 0 | 0 | ORR |  | Hazmi and Wagner (2010c) |
| *Rupilia* Clark, 1864 | 15 | 0 | 0 | AUR |  |  |
| *Ruwenzoria* Laboissière, 1919 | 2 | 0 | 0 | AFR |  | Beenen (2011b) |
| *Sakaramya* Bechyné, 1952 | 1 | 0 | 0 | AFR (Madagascar) |  |  |
| *Salaminia* Heller, 1898 | 2 | 0 | 0 | ORR (Sulawesi) | ^*^*Salamis* Chapuis, 1875 | Heller (1898), Aslam (1972) |
| *Samoria* Silfverberg, 1982 | 8 | 0 | 0 | AFR | ^∆^*Laetanella* Silfverberg, 1982 | Silfverberg (1982a) |
| *Sarawakiola* Mohamedsaid, 1997 | 1 | 0 | 0 | ORR (Borneo) |  | Mohamedsaid (1997e) |
| *Sardoides* Jacoby, 1895 | 3 | 0 | 0 | AFR |  |  |
| *Sastra* Baly, 1865 | 14 | 0 | 0 | AUR, ORR, PAR (Nepal) | ^*^*Eriosardella* Chûjô, 1935 | Aslam (1972), Beenen (2010b), Kimoto (1979), Medvedev (2003b, 2015b), Mohamedsaid (1997f), Shute (1983), Takizawa (1988b) |
| *Sastracella* Jacoby, 1899 | 14 | 0 | 0 | ORR, PAR |  | Aslam (1972), Kimoto (1989a, 2001), Medvedev (2007b), Shute (1983), Yang et al (1995) |
| *Sastroides* Jacoby, 1884 | 23 | 0 | 0 | ORR, PAR |  | Aslam (1972), Bezděk (2009a, 2012b), Kimoto (1979, 1989a, 2003, 2004), Medvedev (2000b, 2009a), Mohamedsaid (1994e, 1999c), Shute (1983), Yu, Wang and Yang (1996) |
| *Scelida* Chapuis, 1875 | 13 | 0 | 0 | NAR, NTR |  | Riley, Clark and Gilbert (2001) |
| *Scelidacne* Clark, 1998 | 1 | 0 | 0 | NTR (Mexico) |  | Clark (1998) |
| *Scelolyperus* Crotch, 1874 | 35 | 0 | 0 | NAR, NTR (Mexico), PAR | ^*^*Eugalera* Brancsik, 1899; ^*^*Tuomuria* Chen and Jiang, 1985; ^*^*Tuomeria* Chen and Jiang, 1986 | Beenen and Bezděk (2007), Bezděk (2007a, 2015a), Borowiec (2005), Chen and Jiang (1985), Chen, Wang and Jiang (1986a), Clark (1996), Gilbert and Andrews (1999), Hatch (1971), Medvedev (1975, 2012e) |
| *Scruptoluperus* Laboissière, 1925 | 3 | 0 | 0 | AFR |  |  |
| *Sermylassa* Reitter, 1913 | 1 | 0 | 0 | PAR | ^*^*Sermyla* Chapuis, 1875 |  |
| *Sermyloides* Jacoby, 1884 | 41 | 1 | 0 | ORR, PAR | ^*^*Praeochralea* Duvivier, 1885 | Bolz and Wagner (2014b), Kimoto (1989a), Li and Yang (2002), Medvedev (2000c, 2013d, 2015a), Mohamedsaid (1997c, 2001c, 2001e, 2002b), Reid (1998b), Yang (1991b, 1991e, 1993a, 1995b) |
| *Sesselia* Laboissière, 1931 | 7 | 0 | 0 | AFR |  | Bryant (1958) |
| *Shaira* Maulik, 1936 | 9 | 0 | 0 | ORR, PAR |  | Beenen (2011a, 2013b), Chen and Jiang (1987b), Chen, Jiang and Wang (1987), Kimoto (1982b), Lopatin (2006b) |
| *Shairella* Chûjô, 1962 | 1 | 0 | 0 | PAR (China: Taiwan) |  | Kimoto (1984), Kimoto and Chu (1996) |
| *Shairoidea* Beenen, 2013 | 3 | 0 | 0 | ORR (Borneo) |  | Beenen (2013b) |
| *Shamshera* Maulik, 1936 | 1 | 0 | 0 | ORR, PAR |  |  |
| *Shensia* Chen, 1964 | 1 | 0 | 0 | PAR (Shaanxi) |  |  |
| *Shungwayana* Silfverberg, 1975 | 1 | 0 | 0 | AFR |  | Silfverberg (1975a) |
| *Schematiza* Chevrolat, 1836 | 37 | 3 | 0 | NTR |  |  |
| *Schematizella* Jacoby, 1888 | 9 | 1 | 0 | AFR | ^*^*Hystaspes* Jacoby, 1903 |  |
| *Siemssenius* Weise, 1922 | 13 | 0 | 0 | ORR (Vietnam), PAR | ^*^*Pseudoliroetis* Laboissière, 1929 | Bezděk (2012b), Bezděk, Romantsov and Medvedev (2014), Jiang (1992), Kimoto (1977, 1989a), Lee (2016a), Warchałowski (2008), Zhang, Li and Yang (2008a) |
| *Sikkimia* Duvivier, 1891 | 9 | 0 | 0 | ORR, PAR (China: Yunnan, Taiwan) | ^*^*Taiwanolepta* Kimoto, 1989; ^*^*Yunomela* Chen, 1964; ^*^*Vietocerus* Lopatin, 2003 | Bezděk and Zhang (2006b), Kimoto (1989b), Lee and Bezděk (2016a), Lopatin (2003a) |
| *Simopsis* Blake, 1966 | 1 | 0 | 0 | NTR |  |  |
| *Sinoluperoides* Kimoto, 1989 | 5 | 0 | 0 | ORR |  | Kimoto (1989a), Medvedev (2012b) |
| *Sinoluperus* Gressitt and Kimoto, 1963 | 4 | 0 | 0 | ORR, PAR |  | Lopatin (2008c), Mohamedsaid (1999b), Yang, Wang and Wu (1998) |
| *Socorroita* Bechyné, 1956 | 2 | 0 | 0 | NTR (Colombia) |  |  |
| *Sonchia* Weise, 1901 | 1 | 0 | 0 | AFR |  | Berti (1990b) |
| *Sonyadora* Bechyné, 1958 | 11 | 0 | 0 | NTR |  |  |
| *Sosibiella* Jacoby, 1896 | 1 | 0 | 0 | ORR |  |  |
| *Sphenoraia* Clark, 1865 | 24 | 0 | 0 | ORR, PAR | ^*^*Neosermylassa* Chûjô, 1956; ^*^*Sphenoraioides* Laboissière, 1934 | Beenen (2005b, 2010b), Jacoby (1895), Jiang (1992), Kimoto (1982a, 1986, 1989a), Kimoto and Takizawa (1972, 1983), Lee (2014b), Lopatin (2002b, 2005a), Medvedev (2001c), Mohamedsaid (2000a, 2001e), Reid (1998b), Wang, Li, and Yang (2000), Yang and Li (1998) |
| *Sphenorella* Medvedev and Sprecher-Uebersax, 1998 | 1 | 0 | 0 | PAR (Nepal) |  | Kimoto and Takizawa (1972, 1983), Medvedev and Sprecher-Uebersax (1998) |
| *Spilocephalus* Jacoby, 1888 | 4 | 0 | 0 | AFR |  |  |
| *Spilonotella* Cockerell, 1905 | 1 | 0 | 0 | AFR | ^*^*Spilonota* Weise, 1902; ^*^*Spilonotula* Weise, 1924 | Silfverberg (1975a) |
| *Spitiella* Laboissière, 1931 | 2 | 0 | 0 | PAR |  | Kimoto (1977, 1979) |
| *Stenellina* Cockerell, 1905 | 4 | 0 | 0 | AFR | ^*^*Stenella* Weise, 1902 |  |
| *Stenoplatys* Baly, 1861 | 4 | 0 | 0 | AFR |  | Berti (1970a) |
| *Stictocema* Jacoby, 1906 | 5 | 0 | 0 | AFR |  |  |
| *Striganovia* Medvedev, 2002 | 1 | 0 | 0 | ORR (Sulawesi) |  | Medvedev (2002c) |
| *Strobiderus* Jacoby, 1884 | 22 | 0 | 0 | AFR, ORR, PAR | ^*^*Syoplia* Jacoby, 1886 | Basu and Halder (1987), Berti (1986b), Kimoto (1977, 1989a), Laboissière (1936b), Medvedev (2015b), Medvedev and Beenen (2010), Yang (1992a, 1992e) |
| *Strumatea* Baly, 1886 | 1 | 0 | 0 | ORR (Borneo) |  |  |
| *Sumatrasia* Jacoby, 1884 | 2 | 0 | 0 | ORR |  |  |
| *Synetocephalus* Fall, 1910 | 11 | 0 | 0 | NAR |  | Gilbert and Clark (2012), Riley, Clark and Gilbert (2001) |
| *Synodita* Chapuis, 1875 | 5 | 0 | 0 | AUR, ORR |  |  |
| *Syphaxia* Baly, 1866 | 2 | 0 | 0 | NTR |  |  |
| *Taenala* Silfverberg, 1978 | 2 | 0 | 0 | AFR |  | Silfverberg (1978a) |
| *Taiwanaenidea* Kimoto, 1984 | 4 | 0 | 0 | PAR (China: Taiwan) |  | Kimoto (1984), Lee and Beenen (2015b) |
| *Taphina* Duvivier, 1885 | 1 | 0 | 0 | ORR |  |  |
| *Tarachodia* Weise, 1902 | 1 | 0 | 0 | AFR |  |  |
| *Taumacera* Thunberg, 1814 | 88 | 0 | 0 | AFR, ORR, PAR | ^*^*Nacrea* Baly, 1886; ^*^*Metellus* Jacoby, 1886; ^*^*Neocharis* Jacoby, 1881; ^*^*Platyxantha* Baly, 1864; ^*^*Doridea* Baly, 1864; ^*^*Platyxanthoides* Laboissière, 1933; ^*^*Luperomorphella* Chûjô, 1964 | Aslam (1972), Beenen (2010b, 2011), Bezděk (2006c), Bryant (1958), Jacoby (1891, 1895), Kimoto (1989a, 2000b, 2004), Medvedev (2001b, 2002a, 2008b), Medvedev and Romantsov (2013), Mohamedsaid (1993a, 1994d, 1995b, 1998d, 1998f, 1999d, 2001a, 2001c, 2001e, 2002b, 2004, 2010), Mohamedsaid and Constant (2007), Medvedev and Sprecher-Uebersax (1998), Reid (1998b, 1999, 2001a) |
| *Theone* Gistl, 1857 | 4 | 12 | 0 | PAR | ^*^*Leptosonyx* Weise, 1885; ^*^*Leptonyx* Jacobson, 1895 | Mandl (1970) |
| *Theopea* Baly, 1864 | 32 | 2 | 0 | ORR, PAR | ^*^*Ozomena* Chevrolat, 1836 | Kimoto (1989a, 1989b, 1989c), Medvedev (2007a, 2002a, 2012b, 2015a), Mohamedsaid (1998c, 2000a), Takizawa (1978, 1988b) |
| *Theopella* Laboissière, 1940 | 1 | 0 | 0 | ORR (Bojo Island) |  | Aslam (1972) |
| *Therpis* Weise, 1900 | 1 | 0 | 0 | AFR | ^*^*Mesotoma* Jacoby, 1903 |  |
| *Trachyscelida* Horn, 1893 | 7 | 2 | 0 | NAR, NTR | ^*^*Racenisa* Bechyné, 1958 | Bechyné and Springlová-Bechyné (1970) |
| *Triarius* Jacoby, 1887 | 6 | 0 | 0 | NAR, NTR (Mexico) |  | Riley, Clark and Gilbert (2001) |
| *Trigonexora* Bechyné and Bechyné, 1970 | 4 | 0 | 0 | NTR |  |  |
| *Trichobalya* Weise, 1924 | 5 | 0 | 0 | ORR, PAR | ^*^*Trichidea* Baly, 1890 | Kimoto (1977, 1982a, 1989a), Mohamedsaid (1999f) |
| *Trichobrotica* Bechyné, 1956 | 21 | 3 | 0 | NTR | ^*^*Iceloceras* Blake, 1958 | Bechyné (1997) |
| *Trichocneorane* Laboissière, 1935 | 3 | 0 | 0 | AFR |  |  |
| *Tricholochmaea* Laboissière, 1932 | 21 | 2 | 0 | NAR, PAR |  | Beenen (2010c), Xue and Yang (2010) |
| *Trichomimastra* Weise, 1922 | 22 | 1 | 0 | ORR, PAR |  | Bezděk (2009a, 2010c, 2013c), Kimoto (1989a), Kimoto and Takizawa (1972), Lopatin (1979, 2003b), Medvedev (1974, 2000d, 2010, 2011c, 2012e, 2013c, 2013e), Mohamedsaid (1998h, 1999d, 2000a), Takizawa (1985a, 1986a) |
| *Trichosepharia* Laboissière, 1936 | 1 | 0 | 0 | ORR (Vietnam) |  |  |
| *Trirhabda* Leconte, 1865 | 31 | 1 | 3 | NAR, NTR (Mexico) |  | Hatch (1971), Riley, Clark and Gilbert (2001), Riley, Clark and Seeno (2003), White (1979) |
| *Tschitscherinula* Jacobson, 1908 | 1 | 0 | 0 | PAR |  |  |
| *Uaupesia* Bechyné, 1958 | 8 | 0 | 0 | NTR |  | Moura (2009) |
| *Vietoluperina* Medvedev, 2015 | 1 | 0 | 0 | ORR (Vietnam) |  | Medvedev (2015a) |
| *Vietoluperus* Medvedev and Dang, 1981 | 1 | 0 | 0 | ORR |  | Bezděk (2012b), Medvedev and Dang (1981), Medvedev (2009f) |
| *Vitruvia* Jacoby, 1903 | 4 | 0 | 0 | AFR |  |  |
| *Witteochaloenus* Laboissière, 1940 | 1 | 0 | 0 | AFR (Congo) |  |  |
| *Xanthogaleruca* Laboissière, 1934 | 9 | 0 | 0 | PAR |  | Beenen (2010c), Gök, Aslan and Aslan (2007), Nie et al (2012), Silfverberg (1974), Xue and Yang (2010) |
| *Xenarthra* Baly, 1861 | 4 | 0 | 0 | ORR (Sri Lanka) |  |  |
| *Xenarthracella* Laboissière, 1940 | 1 | 1 | 0 | AFR |  |  |
| *Xenoda* Baly, 1877 | 24 | 0 | 0 | ORR | ^∆^*Xenodania* Medvedev, 2004; ^∆^*Xenodella* Weise, 1922; ^∆^*Xenodina* Medvedev, 2004; ^∆^*Paraxenidea* Medvedev, 2004 | Medvedev (2004c), Mohamedsaid (2001b) |
| *Xingeina* Chen, Jiang and Wang, 1987 | 4 | 0 | 0 | PAR |  | Chen, Jiang and Wang (1987), Lopatin (2006b) |
| *Yingaresca* Bechyné, 1956 | 51 | 0 | 0 | NTR |  | Bechyné (1997), Blake (1970b, 1971), Cabrera (1998), Moura (2016) |
| *Yulenia* Jacoby, 1886 | 7 | 0 | 0 | AUR, ORR |  | Shute (1983) |
| *Yunaspes* Chen, 1976 | 2 | 0 | 0 | PAR |  | Chen et al (1976), Jiang (1988c) |
| *Yunnaniata* Lopatin, 2009 | 1 | 0 | 0 | PAR (China: Yunnan) |  | Bezděk and Beenen (2009), Lopatin and Konstantinov (2009) |
| *Zangastra* Chen and Jiang, 1981 | 7 | 0 | 0 | PAR |  | Chen and Jiang (1981), Jiang (1988c), Lopatin (2007), Takizawa (1988a) |
| *Zangia* Chen, 1976 | 5 | 0 | 0 | PAR |  | Chen et al. (1976), Jiang (1990c) |
| *Zepherina* Bechyné, 1958 | 44 | 0 | 0 | NTR |  | Bechyné (1997), Bechyné and Springlová-Bechyné (1967, 1976) |
| *Zischkaita* Bechyné, 1956 | 9 | 0 | 0 | NTR |  | Bechyné and Springlová-Bechyné (1970), Moura (2003, 2005) |
| *Zizonia* Chen, 1976 | 1 | 0 | 0 | PAR (China: Xizang) |  | Chen et al. (1976) |
| Total | 7132 | 192 | 12 |  |  |  |

Note：*#* Currently only one species in the genus *Barombiella* (see Wagner & Freund 2003), seven species will be transferred elsewhere in upcoming revisions (Wagner 2017, pers. comm.)

**References**

Abdullah M, Qureshi SS (1968a) The Chrysomelidae, Coleoptera of Pakistan. Part III. – A key to the genera and species of the Galerucinae (Coleoptera: Chrysomelidae), with descriptions of new genera and species. Pakistan Journal of Scientific and Industrial Research 11: 396-414.

Abdullah M, Qureshi SS (1968b) A new genus and species of the Galerucinae (Coleoptera: Chrysomelidae) from pear tree in West Pakistan. Pakistan Journal of Scientific and Industrial Research 11: 423-424.

Abdullah M, Qureshi SS (1968c) *Ashrafia anwarullahi*, a new genus and species of the Galerucinae (Coleoptera: Chrysomelidae) from West Pakistan. Pakistan Journal of Scientific and Industrial Research 11: 425-426.

Allard E (1890) Troisième note sur les galérucides. Bulletin ou Comptes-Rendus des Séances de la Société Entomologique de Belgique 1890: lxxx-xciv.

Anand RK, Cox ML (1986) Taxonomic revision of the genus *Aulacophora* Chevr. I. Species with yellow elytra (Coleoptera, Chrysomelidae, Galerucinae). Entomologische Abhandlungen 50: 81-91.

Andrews FG, Gilbert AJ (2005) A preliminary annotated checklist and evaluation of the diversity of the Chrysomelidae (Coleoptera) of the Baja California peninsula, Mexico. Insecta Mundi 19: 89-116.

Apfelbeck V (1912) Komponente balkanske faune iz roda Chrysomelidae. Glasnik Zemaljskog Muzeja u Bosni i Hercegovini 24: 235-263.

Aslam NA (1972) On the genus *Drasa* Bryant (Coleoptera, Chrysomelidae, Galerucinae) with some nomenclatorial notes on the Galerucinae. Journal of Natural History 6: 483-501. doi: 10.1080/00222937200770451.

Barroga GF (2001a) *Aulacophora nusantara*, a new species of Galerucinae from peninsular Malaysia (Coleoptera: Chrysomelidae). Serangga 6: 1-5.

Barroga GF (2001b) The genus *Aulacophora* Chevrolat (Coleoptera: Chrysomelidae: Galerucinae) from Bali, Indonesia, with a description of a new species. Serangga 6: 37-50.

Barroga GF (2001c) Redescriptions of *Aulacophora* species (Coleoptera: Chrysomelidae: Galerucinae) described by Chapuis from the Philippines. Serangga 6: 321-345.

Barroga GF (2002a) *Aulacophora indica* (Gmelin), the correct name for the squash beetle in the Philippines (Coleoptera: Chrysomelidae: Galerucinae). Philippine Entomologist 16: 25-32.

Barroga GF (2002b) Corrections to the article "Revision of the genus *Aulacophora* Chevrolat (Coleoptera: Chrysomelidae: Galerucinae) in Sundaland". Philippine Entomologist 16: 185-194.

Barroga GF, Mohamedsaid MS (2002) Revision of the genus *Aulacophora* Chevrolat (Coleoptera: Chrysomelidae: Galerucinae) in Sundaland. Serangga 7: 15-194.

Basu CR (1985) Insecta: Coleoptera: Chrysomelidae. Records of the Zoological Survey of India 82: 201-214.

Basu CR (1996) Insecta Coleoptera Chrysomelidae. In: Ghosh AK (Ed) Fauna of West Bengal. Part 6B (Insecta: Coleoptera). State Fauna Series 3. Zoological Survey of India, Calcutta, 559-773.

Basu CR, Halder SK (1987) Insecta: Coleoptera: Chrysomelidae. In: Lamba BS (Ed) Fauna of Orissa, Part 1. Zoological Survey of India, Calcutta 213-240.

Bauer S, Wagner T (2010) Revalidation and revision of *Dyolania* Laboissière, 1931 (Coleoptera: Chrysomelidae, Galerucinae). Entomologische Zeitschrift 120: 165-170.

Bechyné J (1964) Notizen zu den madagassischen Chrysomeloidea (Col. Phytophaga). Mitteilungen der Münchner Entomologischen Gesellschaft 54: 68-161. http://www.zobodat.at/pdf/MittMuenchEntGes_054_0068-0161.pdf.

Bechyné J (1971) *Diabrotica* nouveaux ou peu connus (Col. Phytophaga, Galerucidae). Bulletin Mensuel de la Société Linnéenne de Lyon 40: 288-292.

Bechyné J (1997) Evaluación de los datos sobre los Phytophaga dañinos en Venezuela (Coleoptera). Parte II. Boletín de Entomología Venezolana, Serie Monografías 1: 279-459.

Bechyné J, Springlová-Bechyné B (1961) Insecta Amapaensia: Chrysomeloidea (Col.). Studia Entomologica 4: 409-428.

Bechyné J, Springlová-Bechyné B (1967) Notes sur les Phytophaga neotropicaux (Coleoptera). Revista de la Facultad de Agronomía 4 (2): 5-47.

Bechyné J, Springlová-Bechyné B (1969) Notas sobre Phytophaga Americanos (Coleoptera). Revista de la Facultad de Agronomía 5 (3): 5-64.

Bechyné J, Springlová-Bechyné B (1970) Beiträge zur Galerucidenfauna Boliviens (Col. Phytophaga). Veröffentlichungen der zoologischen Staatssammlung München 14: 121-190. http://www.zobodat.at/pdf/VeroeffZSM_014_0121-0190.pdf.

Bechyné J, Springlová-Bechyné B (1976) Phytophages (Coléopteres) récoltés en Guyane Française par la mission du Muséum National d´Histoire Naturelle. Annales de la Société Entomologique de France (N. S.) 12: 527-556.

Beenen R (1992) The identity of *Luperus* (*Calomicrus*) *minutus* Joannis, 1865 (Coleoptera: Chrysomelidae). Entomologische Berichten (Amsterdam) 52: 141-143.

Beenen R (1996) The forgotten *Adimonia scutellata* Chevrolat (Coleoptera: Chrysomelidae). Entomologische Blätter 92: 85-89.

Beenen R (1998) *Galerucella placida* a proper species from south east Asia (Coleoptera: Chrysomelidae: Galerucinae). Serangga 3: 107-110.

Beenen R (1999) Revisional notes on *Galeruca* 1 (Coleoptera, Chrysomelidae). Entomologische Blätter 95: 85-92.

Beenen R (2002) Revisional notes on *Galeruca* 2 (Coleoptera, Chrysomelidae). Entomologische Blätter 98: 21-28.

Beenen R (2003a) *Galeruca* (*Galemira* n. subgen.) *subcostata* n. sp. from Pakistan (Coleoptera: Chrysomelidae). Stuttgarter Beiträge zur Naturkunde Serie A (Biologie) 648: 1-9. http://www.naturkundemuseum-bw.de/sites/default/files/publikationen/serie-a/A648.pdf.

Beenen R (2003b) New records of *Xanthogaleruca subcoerulescens* (Weise) in southern Turkey (Coleoptera, Chrysomelidae, Galerucinae). Entomologische Blätter 99: 99-103.

Beenen R (2004) *Oides* *bertiae*, a new Galerucinae species from Central Africa (Coleoptera, Chrysomelidae). Journal of Afrotropical Zoology 1: 73-76.

Beenen R (2005a) *Cerophysa erberi* n. sp. from Swat in Pakistan (Coleoptera: Chrysomelidae: Galerucinae). Genus 16: 383-388. http://www.biol.uni.wroc.pl/cassidae/Cerophysa%20erberi.pdf

Beenen R (2005b) The identity of *Galeruca circassica* Reitter and *G. bicolor* Kollar & Redtenbacher (Coleoptera, Chrysomelidae) (Revisional notes on *Galeruca* 4). Entomologische Blätter 101: 13-20.

Beenen R (2007a) *Pseudoshaira*, a new leaf beetle genus occurring in Borneo (Coleoptera: Chrysomelidae: Galerucinae). Genus 18: 597-602. http://www.biol.uni.wroc.pl/cassidae/Beenen_Pseudoshaira.pdf

Beenen R (2007b) Revisional notes on *Galeruca* 6: the species group of *Galeruca interrupta* (Coleoptera, Chrysomelidae). Entomologische Blätter 102 (2006): 157-164.

Beenen R (2007c) Un intéressant Coléoptère phytophage au Muséum d´Histoire Naturelle de Perpignan (Coleoptera: Chrysomelidae). Annales du Muséum d'Histoire Naturelle de Perpignan 15: 3-8.

Beenen R (2008a) Taxonomical and nomenclatural changes in Palaearctic Galerucinae and description of a new species (Chrysomelidae). Entomologische Blätter 103: 63-80.

Beenen R (2008b) Contribution to the knowledge of Galerucinae of New Caledonia (Coleoptera: Chrysomelidae). Genus 19: 65-87. http://www.biol.uni.wroc.pl/cassidae/Beenen_Galerucinae%20New%20Caledonia.pdf.

Beenen R (2009) A new genus and new species of Galerucinae from Chin State, Myanmar (Coleoptera: Chrysomelidae). Genus 20: 327-333. http://www.biol.uni.wroc.pl/cassidae/Beenen_Leptarthroides_low.pdf.

Beenen R (2010a) Three new galerucine species from high altitude habitats in Africa and additional information on described species (Coleoptera: Chrysomelidae, Galerucinae). Entomologische Zeitschrift 120: 76-80.

Beenen R (2010b) New acts and comments. Chrysomelidae: Galerucinae. In: Löbl I, Smetana A (Eds) Catalogue of Palaearctic Coleoptera, Volume 6. Chrysomeloidea. Apollo books, Stenstrup, 74-75.

Beenen R (2011a) Chrysomelidae: Galerucinae. In: Löbl I, Smetana A (Eds) Catalogue of Palaearctic Coleoptera, Volume 7. Chrysomeloidea. Apollo books, Stenstrup, 443-491.

Beenen R (2011b) Two new *Rohania* species from Central Africa and additional information on other species in this genus (Coleoptera, Chrysomelidae, Galerucinae). Entomologische Blätter 107: 7-16.

Beenen R (2011c) Errata for volume 6. Chrysomelidae: Galerucinae. In: Löbl I, Smetana A (Eds) Catalogue of Palaearctic Coleoptera, Volume 7 Curculionoidea I. Apollo books, Stenstrup, 35-61.

Beenen R (2012) Bestimmungsschlüssel der afrikanische Gattung *Eupachytoma* Laboissière nebst faunistisch Bemerkungen (Coleoptera, Chrysomelidae). Entomologische Blätter und Coleoptera 108: 133-140.

Beenen R (2013a) Contribution to the knowledge of Galerucinae of New Caledonia 2 (Coleoptera: Chrysomelidae). Genus 24: 65-108. http://www.biol.uni.wroc.pl/cassidae/Beenen_Galerucinae%20of%20New%20Caledonia%202_low.pdf.

Beenen R (2013b) New species and a new genus of brachelytrous Galerucinae (Coleoptera: Chrysomelidae). Entomologische Zeitschrift 123: 173-183.

Beenen R (2014a) *Clitenososia vazquezi* sp. nov. from Kenya (Coleoptera: Chrysomelidae: Galerucinae). Genus 25: 451-457. http://www.biol.uni.wroc.pl/cassidae/Beenen_Clitenososia%20vazquezi.pdf.

Beenen R (2014b) *Diacantha kippenbergi* a new African galerucine leaf beetle (Coleoptera, Chrysomelidae). Entomologische Blätter und Coleoptera 110: 83-86.

Beenen R (2014c) Key to the species of *Radymna* Reitter, 1913 with taxonomic and faunistic comments and desription of two new species (Coleoptera, Chrysomelidae, Galerucinae). Entomologische Blätter und Coleoptera 110: 87-100.

Beenen R (2015) A new species of the genus *Mahutia* Laboissière and considerations on the distribution of the species of this African genus (Coleoptera, Chrysomelidae, Galerucinae). Entomologische Blätter und Coleoptera 111: 1-4.

Beenen R (2016) Notes on African Galerucini and descriptions of new species (Coleoptera, Chrysomelidae, Galerucinae). Entomologische Blätter und Coleoptera 112: 25-33.

Beenen R, Bezděk J (2007) A new species of *Scelolyperus* from Central Asia and a key to the Palaearctic species (Coleoptera, Chrysomelidae). Entomologische Blätter für Biologie und Systematik der Käfer 102: 87-93.

Beenen R, Hawkeswood TJ (2004) *Cydippa balyi* Chapuis, 1875 (Coleoptera, Chrysomelidae), an interesting galerucine from Australia rediscovered in the Northern Territory, with redescription and notes on its habitat and host plant. In: Jolivet P, Santiago-Blay JA, Schmitt M (Eds) New developments in the Biology of Chrysomelidae. SPB Academic Publishing, Hague, 469-473.

Beenen R, Lee CF (2010) Two new *Erganoides* species from P. R. China and Taiwan (Coleoptera: Chrysomelidae: Galerucinae). Genus 21: 257-264. http://www.biol.uni.wroc.pl/cassidae/Beenen_Two%20Erganoides.pdf.

Beenen R, Warchałowski A (2010) *Charaea pseudominutum* n. sp., an undescribed but not unknown galerucine beetle (Coleoptera, Chrysomelidae, Galerucinae). Entomologische Blätter 106: 57-62.

Beenen R, Yang XK (2007) A new species of the genus *Galeruca* Müller from China (Coleoptera, Chrysomelidae, Galerucinae). Acta Zootaxonomica Sinica 32: 67-69.

Berti N (1970a) Une nouvelle espèce du genre *Stenoplatys* Baly (Col. Chrysomelidae Galerucinae). Bulletin de la Société Entomologique de France 75: 36-39.

Berti N (1970b) *Protocoelocrania nigra* n. sp. un chrysomélide nouveau de République Centrafricaine (Coléoptère, Galerucinae). Annales du Centre d'Enseignement Supérieur de Brazzaville 6: 157-159.

Berti N (1983) Les *Medythia* afrotropicaux et malgaches. Description de deux espèces nouvelles (Coleoptera, Chrysomelicae, Galerucinae). Revue Francaise d'Entomologie (N. S.) 5: 95-100.

Berti N (1986a) Galerucinae afrotropicaux quatre espèces confondues sous le nom d´*Oides* minor description des espèces et sous-espèces nouvelles (Coleoptera, Chrysomelidae). Nouvelle Revue d'Entomologie (N. S.) 3: 47-64.

Berti N (1986b) Les *Strobiderus* Afrotropicaux et description d'une espèce nouvelle: *Strobiderus carayoni*, n. sp (Coleoptera, Chrysomelicae, Galerucinae). Annales de la Société Entomologique de France 22: 291-299.

Berti N (1987) *Impensa fulva* (Laboissière) nov. comb (Col. Chrysomelidae). Nouvelle Revue d’Entomologie (N. S.) 4: 36.

Berti N (1988) Taxonomie et note synonymique sur les *Impensa* afrotropicaux (Coleoptera, Chrysomelidae). Nouvelle Revue d’Entomologie (N. S.) 5: 339-348.

Berti N (1989a) Types du genre *Lamprocopa* Hincks examines et designes. Note synonymique (Col. Chrysomelidae). Nouvelle Revue d’Entomologie (N. S.) 6: 207-208.

Berti N (1989b) Types du genre *Lamprocopa* Hincks examines et designes (Col. Chrysomelidae). Nouvelle Revue d’Entomologie (N. S.) 6: 230.

Berti N (1990a) A propos de *Leptaulaca labiata* Jacoby, Galerucinae Luperini africain, nouveau synonyme de *Lamprocopa praecox* (Klug) (Coleoptera: Chrysomelidae). Annales de la Société Entomologique de France 26: 124.

Berti N (1990b) Contribution à l´étude des Galerucinae afrotropicaux. IX. - Le genre *Aulacophora* Chevrolat, description d´un genre nouveau, *Chosnia* gen. nov (Coleoptera, Chrysomelidae). Journal of African Zoology 104: 109-126.

Berti N (1993) Galerucinae afrotropicaux. Étude des *Oides* Weber, 1801. II. Le groupe collaris (Coleoptera, Chrysomelidae). Bulletin de la Société Entomologique de France 98: 233-262.

Berti N, Rapilly M (1973) Contribution a la faune de l'Iran. Voyages de MM. R. Naviaux et M. Rapilly (Col. Chrysomelidae). Annales de la Société Entomologique de France 9: 861-894.

Berti N, Rapilly M (1983) Une nouvelle espèce de *Galerima* pour la faune espagnole: *G. villiersi* (Coléoptères, Chrysomelidae, Galerucinae). L’Entomologiste 39: 273-279.

Bezděk J (1998) *Monolepta anatolica* sp. n. – a new species of leaf beetle (Coleoptera: Chrysomelidae: Galerucinae) from Turkey. Klapalekiana 34: 149-152.

Bezděk J (2002) *Phyllobrotica aslani* Warchalowski, 1998, a new synonym of *P. binotata* Ogloblin, 1936 (Coleoptera: Chrysomelidae: Galerucinae). Genus 13: 351-352. http://www.biol.uni.wroc.pl/cassidae/Phyllobroticasynonym.pdf.

Bezděk J (2003a) Studies on Asiatic *Apophylia*. Part 1: new synonyms, lectotype designations, redescriptions, descriptions of new species and notes (Coleoptera: Chrysomelidae: Galerucinae). Genus 14: 69-102. http://www.biol.uni.wroc.pl/cassidae/Apophylia.pdf

Bezděk J (2003b) Studies on Asiatic *Apophylia* (Insecta: Coleoptera: Chrysomelidae). Part 2: Revision of the *aeruginosa* and *lebongana* species groups. Stuttgarter Beiträge zur Naturkunde Serie A (Biologie) 649: 1-11. http://www.naturkundemuseum-bw.de/sites/default/files/publikationen/serie-a/A649.pdf.

Bezděk J (2003c) Studies on asiatic *Apophylia*. Part 3. Revisional study of type materials and descriptions of eight species new to science (Coleoptera: Chrysomelidae: Galerucinae). Genus 14: 191-230. http://www.biol.uni.wroc.pl/cassidae/Apophylia%20part2.pdf

Bezděk J (2003d) Studies on asiatic *Apophylia*. Part 5: Revisional study of type materials and descriptions of five species new to science (Chrysomelidae: Galerucinae). Genus 14: 479-510. http://www.biol.uni.wroc.pl/cassidae/Apophylia5.pdf.

Bezděk J (2004a) A review of the *Lochmaea crataegi* (Forster, 1771) species group from Asia Minor, Near East and Caucasus (Coleoptera: Chrysomelidae: Galerucinae). Annales Zoologici 54: 561-566.

Bezděk J (2004b) Studies on asiatic *Apophylia* Thomson, 1858 (Chrysomelidae: Galerucinae). Part 4. Revision of *A. celebensis* Pic, 1927 and *A. pallipes* (Jacoby, 1892) groups. Russian Entomological Journal 13: 43-51.

Bezděk J (2004c) Revisional study on African *Apophylia*. Part 1 (Coleoptera: Chrysomelidae: Galerucinae). Genus 15: 91-108. http://www.biol.uni.wroc.pl/cassidae/Apophylia%20africana01.pdf.

Bezděk J (2005a) Revisional study of African *Apophylia*. Part 2: Bequaertinia Laboissière, 1922, a new synonym of *Apophylia* Thomson, 1858 (Coleoptera: Chrysomelidae: Galerucinae). Genus 16: 209-222. http://www.biol.uni.wroc.pl/cassidae/Apophylia%20afrika2.pdf

Bezděk J (2005b) Contribution to the knowledge of the genus *Cneorella* Medvedev & Dang, 1981, with descriptions of five species new to science (Coleoptera: Chrysomelidae: Galerucinae). Genus 16: 223-246. http://www.biol.uni.wroc.pl/cassidae/Cneorella.pdf.

Bezděk J (2005c) Revisional study on African *Apophylia*. Part 3 (Coleoptera: Chrysomelidae: Galerucinae). Genus 16: 395-412. http://www.biol.uni.wroc.pl/cassidae/Apophylia%20Africa03.pdf.

Bezděk J (2005d) Revisional study on African *Apophylia* (Coleoptera: Chrysomelidae: Galerucinae). Part 4. Acta Entomologica Musei Nationalis Pragae 45: 165-182. http://aemnp.eu/PDF/45_0/45_0_165.pdf.

Bezděk J (2005e) New and interesting *Apophylia* species from South-East Asia (Coleoptera: Chrysomelidae: Galerucinae). Raffles Bulletin of Zoology 53: 35-45. https://lkcnhm.nus.edu.sg/nus/pdf/PUBLICATION/Raffles%20Bulletin%20of%20Zoology/Past%20Volumes/RBZ%2053(1)/53rbz035-045.pdf

Bezděk J (2006a) *Apophylia phuphanensis* sp. nov (Coleoptera: Chrysomelidae: Galerucinae) from Laos. Folia Heyrovskyana 13: 215-219.

Bezděk J (2006b) Three new species and a new record of *Apophylia* Thomson, 1858 from eastern India (Coleoptera: Chrysomelidae: Galerucinae). Annales Zoologici 56: 259-264.

Bezděk J (2006c) Revisional study on African *Apophylia*. Part 5 (Coleoptera: Chrysomelidae: Galerucinae). Genus 17: 211-244. http://www.biol.uni.wroc.pl/cassidae/Apophylia%20africa5.pdf.

Bezděk J (2006d) *Calomicrus atrocephalus* (Reitter, 1895), a new synonym of *Calomicrus apicalis* Demaison, 1891 (Chrysomelidae: Galerucinae). Genus 17: 359-362. http://www.biol.uni.wroc.pl/cassidae/Calomicrus%20apicalis.pdf.

Bezděk J (2006e) Resurrection of *Hoplasoma simplicipennis* and *H. ventralis*, previously synonymized with *H. unicolor* (Coleoptera: Chrysomelidae: Galerucinae). Acta Entomologica Musei Nationalis Pragae 46: 133-140.

Bezděk J (2007a) Taxonomical changes in Palaearctic Luperini (Coleoptera: Chrysomelidae: Galerucinae). Annales Zoologici 57: 257-266.

Bezděk J (2007b) Two new *Apophylia* species from Maharashtra state, India (Coleoptera: Chrysomelidae: Galerucinae). Genus 18: 271-277. http://www.biol.uni.wroc.pl/cassidae/Bezdek_Two%20new%20Apophylia.pdf.

Bezděk J (2007c) Notes on the Galerucini from India and Sri Lanka, with description of *Pyrrhalta warchalowskii* n. sp. from Tamil Nadu State, India (Coleoptera: Chrysomelidae: Galerucinae). Genus 18: 603-612. http://www.biol.uni.wroc.pl/cassidae/Bezdek_Pyrrhalta%20warchalowskii.pdf.

Bezděk J (2008a) A review of the genus *Hoplasoma* (Coleoptera: Chrysomelidae: Galerucinae) from Sulawesi, Indonesia, with the description of *H. bosi* sp. nov. Zootaxa 1941: 55-66.

Bezděk J (2008b) Taxonomical and faunistical notes on Asian *Apophylia* (Coleoptera: Chrysomelidae: Galerucinae). Annales Zoologici 58: 595-606. doi: 10.3161/000345408X364436.

Bezděk J (2008c) New species and subspecies of *Nymphius* (Coleoptera: Chrysomelidae: Galerucinae) from Iran and Turkey. Acta Entomologica Musei Nationalis Pragae 48: 79-93. http://aemnp.eu/PDF/48_1/48_1_79.pdf.

Bezděk J (2009a) Taxonomic and faunistic notes on Oriental Galerucinae (Coleoptera: Chrysomelidae). Genus 20: 85-103. http://www.biol.uni.wroc.pl/cassidae/Bezdek_Oriental%20Galerucinae.pdf.

Bezděk J (2009b) Revision of the genus *Munina* (Coleoptera: Chrysomelidae: Galerucinae), with the description of *M. laotica* sp. nov. from Laos. Zootaxa 2032: 55-62. doi: 10.5281/zenodo.186287.

Bezděk J (2009c) Revisional study on the genus *Mimastra* (Coleoptera: Chrysomelidae: Galerucinae): Species with unmodified protarsomeres in male. Part 1. Acta Entomologica Musei Nationalis Pragae 49: 819-840. http://aemnp.eu/PDF/49_2/49_2_819.pdf.

Bezděk J (2009d) *Agetocera silva* sp. nov. from the *A. lobicornis* species group (Coleoptera, Chrysomelidae, Galerucinae). Entomologica Basiliensia et Collectionis Frey 31: 209-218.

Bezděk J (2010a) *Phyllobrotica malinka* sp. nov. from Turkey and Iran and a review of allied species (Coleoptera: Chrysomelidae: Galerucinae). Acta Entomologica Musei Nationalis Pragae 50: 563-575. http://aemnp.eu/PDF/50_2/50_2_563.pdf.

Bezděk J (2010b) *Haplomela* Chen, 1942, a new synonym of *Hoplasoma* Jacoby, 1884 (Coleoptera: Chrysomelidae, Galerucinae). Entomologische Zeitschrift 120: 81-84.

Bezděk J (2010c) Revisional study on the genus *Mimastra* (Coleoptera: Chrysomelidae: Galerucinae). Part 2. Annales Zoologici 60: 35-46. doi: 10.3161/000345410x499506.

Bezděk J (2010d) New acts and comments. Chrysomelidae: Galerucinae. In: Löbl I, Smetana A (Eds) Catalogue of Palaearctic Coleoptera, Volume 6. Chrysomeloidea. Apollo books, Stenstrup, 73.

Bezděk J (2011) Revisional study on the genus *Mimastra* (Coleoptera: Chrysomelidae: Galerucinae). Part 3: *Mimastra oblonga* and *M. tarsalis* species groups. Zootaxa 2766: 30-56.

Bezděk J (2012a) Revision of *Hoplasoma* (Coleoptera: Chrysomelidae: Galerucinae) of the Philippines, with descriptions of five new species. Zootaxa 3382: 1-19. doi: 10.5281/ZENODO.210044.

Bezděk J (2012b) Taxonomic and faunistic notes on Oriental and Palaearctic Galerucinae and Cryptocephalinae (Coleoptera: Chrysomelidae). Genus 23: 375-418. http://www.biol.uni.wroc.pl/cassidae/Bezdek_Taxonomic%20notes%20on%20Chrysomelidae_low.pdf.

Bezděk J (2012c) Galerucinae (Coleoptera: Chrysomelidae) of Socotra Island, with a review of taxa recorded from Yemen. In: Hájek J, Bezděk J (Eds) Insect biodiversity of the Socotra Archipelago. Acta Entomologica Musei Nationalis Pragae 52 (supplementum 2): 403-428. http://aemnp.eu/PDF/52_s2/52_s2_403.pdf.

Bezděk J (2013a) Revision of the genus *Hesperopenna* (Coleoptera: Chrysomelidae: Galerucinae). I. Generic redescription, definition of species groups and taxonomy of *H. medvedevi* species group. Acta Entomologica Musei Nationalis Pragae 53: 715-746. http://aemnp.eu/PDF/53_2/53_2_715.pdf.

Bezděk J (2013b) A contribution to knowledge of the genus *Liroetoides* Kimoto, 1989 (Coleoptera, Chrysomelidae, Galerucinae), with description of *L. geiseri* sp. nov. from Laos. Entomologica Basiliensia et Collectionis Frey 34: 341-349. https://biogeography.unibas.ch/PDF/20Bezdek%20EB34.pdf.

Bezděk J (2013c) Redescription of *Mimastra kumatai* (Kimoto & Takizawa, 1972), comb. nov (Coleoptera: Chrysomelidae: Galerucinae). Genus 24: 315-323. http://www.biol.uni.wroc.pl/cassidae/Bezdek_Mimastra%20kumatai_low.pdf

Bezděk J (2014) A revision of *Hoplasoma acuminatum* and *H. thailandicum* species groups, and re-definition of H. unicolor species group (Coleoptera: Chrysomelidae: Galerucinae). Zootaxa 3794: 419-434. doi: 10.11646/zootaxa.3794.3.5.

Bezděk J (2015a) A review of Palaearctic *Scelolyperus* (Coleoptera: Chrysomelidae: Galerucinae), with description of *S. perreus* sp. nov. from Turkey. Annales Zoologici 65: 21-39. doi: 10.3161/00034541ANZ2015.65.1.003.

Bezděk J (2015b) *Charaea luzonicum* sp. nov. (Coleoptera: Chrysomelidae: Galerucinae): the first record of *Charaea* in the Philippines. Revue Suisse de Zoologie 122: 371-375. doi: 10.5281/zenodo.30005.

Bezděk J (2016a) Redescription and identity of *Taphinella bengalensis* Jacoby, 1900 (Coleoptera: Chrysomelidae: Galerucinae). Turkish Journal of Zoology 40: 120-124. doi: 10.3906/zoo-1502-9.

Bezděk J (2016b) Revision of the genus *Coeligetes* from Malaysia and Indonesia, and description of *Coeligetoides* gen. nov (Coleoptera: Chrysomelidae: Galerucinae). Zootaxa 4085: 504-524. doi: 10.11646/zootaxa.4085.4.3.

Bezděk J (2016c) Revision of the *Clytra subfasciata* species group (Coleoptera: Chrysomelidae: Cryptocephalinae: Clytrini). Zoology in the Middle East 62: 148-157. doi: 10.1080/09397140.2016.1182772.

Bezděk J (2016d) Revision of the genus *Hesperopenna* Medvedev et Dang, 1981 (Coleoptera: Chrysomelidae: Galerucinae). II. *H. vietnamica* species group and new taxonomical changes. Studies and Reports, Taxonomical Series 12: 7-27. http://wwwold.fld.czu.cz/studiesandreports/pdf/2016-1/authors/7-28_bezdek.pdf.

Bezděk J, Beenen R (2009) Additions to the description of *Yunnaniata konstantinovi* Lopatin, 2009 and its classification in the section Capulites (Coleoptera: Chrysomelidae: Galerucinae: Hylaspini). Zootaxa 2303: 45-52. doi: 10.5281/ZENODO.191775.

Bezděk J, Lee CF (2009) *Apophylia kaoi* sp. nov. from Taiwan (Coleoptera: Chrysomelidae: Galerucinae). Genus 20: 429-434. http://www.biol.uni.wroc.pl/cassidae/Bezdek_Apophylia%20kaoi_lowres.pdf

Bezděk J, Lee CF (2011) Revisional study on the genus *Mimastra* (Coleoptera: Chrysomelidae: Galerucinae). Part 4. Annales Zoologici 61: 709-729. doi: 10.3161/000345411X622543.

Bezděk J, Lee CF (2014) Revision of *Charaea* (Coleoptera: Chrysomelidae: Galerucinae) from Taiwan. Zootaxa 3861: 1-39. doi: 10.11646/zootaxa.3861.1.1.

Bezděk J, Romantsov PV, Medvedev LN (2014) A review of *Luperogala* Medvedev & Samoderzhenkov, 1989, with description of a new species from Borneo (Coleoptera: Chrysomelidae: Galerucinae). Genus 25: 459-479. http://www.biol.uni.wroc.pl/cassidae/Bezdek%20et%20al%20_Luperogala%20review_low.pdf.

Bezděk J, Sen I, Gök A (2013) *Calomicrus velai* sp. nov. from Iran and redescription of *C. koenigi* (Jacobson, 1897) (Chrysomelidae, Galerucidae, Luperini). Annales Zoologici 63: 365-370. doi: 10.3161/000345413X669612.

Bezděk J, Zhang LJ (2006a) Two new species of *Apophylia* from China (Coleoptera: Chrysomelidae: Galerucinae). Acta Entomologica Musei Nationalis Pragae 46: 145-150. http://aemnp.eu/PDF/46_0/46_0_145.pdf.

Bezděk J, Zhang LJ (2006b) *Yunomela* Chen, 1964 and *Vietocerus* Lopatin, 2003, new synonyms of *Sikkimia* Duvivier, 1891 (Coleoptera: Chrysomelidae: Galerucinae). Genus 17: 351-358. http://www.biol.uni.wroc.pl/cassidae/Sikkimia%20synonyms.pdf.

Bezděk J, Zhang LJ (2007) Taxonomical changes in the genera *Hoplasoma* and *Haplosomoides* (Coleoptera: Chrysomelidae: Galerucinae). Acta Entomologica Musei Nationalis Pragae 47: 189-193. http://aemnp.eu/PDF/47_0/47_0_189.pdf.

Bieńkowski AO (1997) New distributional records for several Palaearctic Chrysomelidae species with some systematic remarks (Insecta: Coleoptera). Faunistische Abhandlungen 21: 91-104.

Bieńkowski AO, Orlova-Bienkowskaja MYa (2013) New data on the composition and distribution of the genus *Leptomona* Bechyné, 1958 (Coleoptera, Chrysomelidae: Galerucinae). Entomological Review 93: 901-903.

Biondi M, D´Alessandro P (2012) Afrotropical flea beetle genera: a key to their identification, updated catalogue and biogeographical analysis (Coleoptera, Chrysomelidae, Galerucinae, Alticini). ZooKeys 253: 1-158. doi: 10.3897/zookeys.253.3414.

Blake DH (1970a) Notes on some chrysomelid beetles from the United States and Argentina. Proceedings of the Entomological Society in Washington 72: 320-324.

Blake DH (1970b) Some new chrysomelid beetles from Cuba. Acta Musei Moraviae 55: 115-126.

Blake DH (1971) Fifteen new West Indian chrysomelid beetles. Proceedings of the Entomological Society in Washington 73: 269-282.

Bolz H, Wagner T (2005) Revision of *Galerudolphia* Hincks, 1949 (Coleoptera: Chrysomelidae, Galerucinae). Insect Systematics & Evolution 35: 361-400. doi: 10.1163/187631204788912436.

Bolz H, Wagner T (2012) *Neobarombiella*, a diverse, newly described genus of Afrotropical Galerucinae (Coleoptera, Chrysomelidae). Zootaxa 3463: 1-112.

Bolz H, Wagner T (2014a) A new Afrotropical *Neobarombiella* species from Socotra Island (Coleoptera: Chrysomelidae: Galerucinae). In: Hájek J, Bezděk J (Eds) Insect biodiversity of the Socotra Archipelago II. Acta Entomologica Musei Nationalis Pragae 54(Supplementum): 277-281. http://aemnp.eu/PDF/54_s/54_s_277.pdf.

Bolz H, Wagner T (2014b) Revision of the Afrotropical *Pimentelia* Laboissière, 1939 (Coleoptera, Chrysomelidae, Galerucinae). Zootaxa 3881: 49-62. doi: 10.11646/zootaxa.3881.1.4.

Bolz H, Wagner T (2014c) *Afrorudolphia* gen. nov., a newly described group of Afrotropical galerucines (Coleoptera: Chrysomelidae, Galerucinae). Entomologische Zeitschrift 124: 149-156.

Borowiec L (2005) A new species of *Scelolyperus* Crotch, 1874 from Kirgizstan (Coleoptera: Chrysomelidae: Galerucinae). Genus 16: 379-382. http://www.biol.uni.wroc.pl/cassidae/Scelolyperus%20kroliki.pdf

Bryant GE (1955) New species of Chrysomelidae (Galerucinae) from Africa. Annals and Magazine of Natural History (12)8: 911-916.

Bryant GE (1956) Contributions à l´étude de la faune entomologique du Ruanda-Urundi (Mission P. Basilewsky 1953). XCVII. Coleoptera Chrysomelidae Donaciinae, Criocerinae, Megalopodinae, Clytrinae, Cryptocephalinae (part), Eumolpinae and Gallerucinae. Annales du Musée Royal du Congo Belge (Tervuren) (Zool.) 81: 535-568.

Bryant GE (1958) Galerucinae (Coleoptera Chrysomelidae). Exploration du Parc National Upemba, I. Mission G. F. de Witte 49 (5): 41-65.

Bryant GE (1959a) Coleoptera: Chrysomelidae I. South African Animal Life 6: 194-226.

Bryant GE (1959b) Chrysomelidae. Ruwenzori Expedition 1952 2: 1-15.

Bryant GE (1960) Mission zoologique de l´I.R.S.A.C. en Afrique orientale (P. Basilewsky et N. Leleup, 1957). XXI. Coleoptera Chrysomelidae. Annales du Musée Royal du Congo Belge (Tervuren) (Zool.) 51: 395-421.

Bukejs A, Bezděk J (2014) *Calomicrus eocenicus* sp. nov (Coleoptera: Chrysomelidae: Galerucinae) from Baltic amber. Baltic Journal of Coleopterology 14: 73-78. http://www.bjc.sggw.pl/arts/2014v14n1/08.pdf.

Cabrera N (1991a) Identidad de *Anisobrotica nordenskioldi* (Jacoby, 1904) (Coleoptera, Chrysomelidae, Galerucinae). Neotrópica (La Plata) 37: 67‑73.

Cabrera N (1991b) Contribucion para el conocimiento de *Itaitubana spinipennis* (Bechyne, 1963) (Coleoptera, Chrysomelidae, Galerucinae). Neotrópica (La Plata) 37: 123‑126.

Cabrera N (1994) Sobre tres especies argentinas del género *Paranapiacaba* Bechyné (Coleoptera, Chrysomelidae, Galerucinae). Neotrópica (La Plata) 40: 19‑27.

Cabrera N (1995) Nuevos aportes para el conocimiento del género *Synbrotica* Bechyné (Coleoptera: Chrysomelidae: Galerucinae). Neotrópica (La Plata) 41: 9‑18.

Cabrera N (1998) Redescripción de *Yingaresca difficillis* (Bowditch, 1923) y descripción de *Yingaresca pindapoyensis* sp. nov (Coleoptera: Chrysomelidae: Ga1erucinae). Neotrópica (La Plata) 44: 107-112.

Cabrera N (1999a) Redescripción de *Cochabamba polychroma* Bechyné y *C. chacoensis* (Bowditch) (Coleoptera: Chrysomelidae: Galerucinae). Neotrópica (La Plata) 45: 69-75.

Cabrera N (1999b) Contribución para el conocimiento del género *Acalymma* en la Argentina (Coleoptera: Chrysomelidae). Revista de la Sociedad Entomologica Argentina 58: 91-105.

Cabrera N (2001a) Estudio sistemático de *Diabrotica* Chevrolat grupo fucata en la Argentina I (Coleoptera, Chrysomelidae). Physis (Secc. C) (Buenos‑Aires) 58: 47‑56.

Cabrera N (2001b) Estudio sistemático de *Diabrotica* Chevrolat grupo fucata en la Argentina II (Coleoptera, Chrysomelidae). Physis (Secc. C) (Buenos‑Aires) 58: 57‑66.

Cabrera N (2001c) *Acalymma xanthographa* sinónimo junior de *A. bivittula bivittula* (Coleoptera: Chrysomelidae: Galerucinae). Neotrópica (La Plata) 47: 107‑108.

Cabrera N, Cabrera Walsh G (2004a) *Platybrotica misionensis* a new genus and species of Luperini (Coleoptera: Chrysomelidae: Galerucinae) from Argentina. Annals of the Entomological Society of America 97: 6-14. doi: 10.1603/0013-8746(2004)097[0006:PMANGA]2.0.CO.

Cabrera N, Cabrera Walsh G (2004b) *Diabrotica calchaqui*, a new species of Luperini (Coleoptera: Chrysomelidae: Galerucinae), from Argentina. Annals of the Entomological Society of America 97: 889-897. doi: 10.1603/0013-8746(2004)097[0889:DCANSO]2.0.CO.

Cabrera N, Cabrera Walsh G (2010) *Diabrotica collicola* (Coleoptera: Chrysomelidae), a new species of leaf beetle from Argentina and key to species of the Diabrotica virgifera group and relatives. Zootaxa 2683: 45-55. doi: 10.5281/zenodo.199370.

Cabrera N, Durante S (2004) The neotropical genus *Caraguata* Bechyné: Description of the previously unknown male of *Caraguata bella* Bechyné (Coleoptera: Chrysomelidae: Galerucinae). Transactions of the American Entomological Society 130: 155‑163.

Cabrera N, Sosa Gómez DR, Micheli A (2008) Morphological and molecular characterization of a new species of *Diabrotica* (Coleoptera, Chrysomelidae, Galerucinae). Zootaxa 1922: 33-46. doi: 10.5281/zenodo.184727.

Chapuis F (1876) Diagnoses des espèces du genre *Aulacophora recueillies* aux iles Philippines par le Dr Semper. Comptes-Rendus des Séances de la Société Entomologique de Belgique 19: xcix-ci.

Chen SH (1964) New genera and species of Galerucinae from China. Acta Entomologica Sinica 13: 201-211.

Chen SH (1978) Atysa cinnamomi - a new galerucine beetle injurious to camphor trees in Fukien. Acta Entomologica Sinica 21: 55-56.

Chen SH, Jiang SQ (1981) Coleoptera: Chrysomelidae - Galerucinae. In: Chen CH (Ed) Insects of Xizang. Volume 1. Peking, Sciences Press, 457-489.

Chen SH, Jiang SQ (1984) New species of Chinese Galerucinae (Coleoptera: Chrysomelidae). Entomotaxonomia 6: 83-92.

Chen SH, Jiang SQ (1985) [new taxa]. In: Huang DS, Han Y, Zhang X (Eds) The insect fauna of the Mt. Tuomuer area in Tianshan. Xinjiang People’s Press, Beijing, 53-165.

Chen SH, Jiang SQ (1986a) On the Chinese species of the galerucine genus *Japonitata* (Coleoptera: Chrysomelidae). Acta Zootaxonomica Sinica 11: 72-79.

Chen SH, Jiang SQ (1986b) Two new species of Galerucinae from Sichuan and Yunnan Provinces (Coleoptera: Chrysomelidae). Acta Zootaxonomica Sinica 11: 198-200.

Chen SH, Jiang SQ (1987a) New galerucine beetles from Xizang (Coleoptera: Chrysomelidae). Sinozoologia 5: 53-59.

Chen SH, Jiang SQ (1987b) Coleoptera: Chrysomelidae-Galerucinae. Agricultural Insects Spiders Plant Diseases and Weeds of Xizang 2: 47-56.

Chen SH, Jiang SQ, Wang SY (1987) New alpine Galerucinae from Hengduan Mountains of Yunnan and Sichuan (Coleoptera: Chrysomelidae). Sinozoologia 5: 61-71.

Chen SH, Wang SY, Jiang SQ (1985) A new genus of Galerucinae from West China (Coleoptera: Chrysomelidae). Acta Zootaxonomica Sinica 31: 372-376.

Chen SH, Wang SY, Jiang SQ (1986a) New leaf beetles from Mount Tuomuer, Xinjiang. Entomotaxonomia 8: 55-58.

Chen SH, Wang SY, Jiang SQ (1986b) The galerucine genus *Capula* from west China (Coleoptera: Chrysomelidae). Acta Zootaxonomica Sinica 11: 398-400.

Chen SH, Yang XK (1992) Descriptions of new and first recorded species of the genus Gallerucida (Coleoptera: Chrysomelidae: Galerucinae). Zoological Research 13: 133-138.

Chen SH, Yu PY, Wang SY, Jiang SQ (1976) New leaf beetles from west China. Acta Entomologica Sinica 19 205-224.

Chûjô M (1962) Coleoptera from Southeast Asia, 24. Family Chrysomelidae. In: Kira T, Umesao T (Eds) Nature and life in Southeast Asia, Vol. 2. Fauna and flora research society, Kyoto, 103-106.

Chûjô M (1964) 32. Family Chrysomelidae. In: Kira T, Umesao T (Eds) Nature and life in Southeast Asia, Vol. 3. Fauna and flora research society, Kyoto, 252-315.

Chûjô M (1966) Chrysomelid-beetles from Northeast Nepal. Memoirs of the Faculty of Education Kagawa University 2 (145): 1-35.

Clark SM (1987) An unusual new genus and species of galerucine beetle from Haiti (Coleoptera: Chrysomelidae). The Coleopterists Bulletin 41: 167-170.

Clark SM (1993) A new genus and two new species of Luperini (Coleoptera: Chrysomelidae: Galerucinae) from Costa Rica. Insecta Mundi 7: 215-218.

Clark SM (1996) The genus *Scelolyperus* Crotch in North America (Coleoptera: Chrysomelidae: Galerucinae). Insecta Mundi 10: 261-280.

Clark SM (1998) Descriptions of new luperine genera and species from Mexico, with keys to related taxa (Coleoptera: Chrysomelidae: Galerucinae). Insecta Mundi 12: 189-206.

Clark SM (1999) The Western North American Genus Androlyperus Crotch, 1873 (Coleoptera: Chrysomelidae: Galerucinae). Insecta Mundi 13: 217-227.

Clark SM, Lillrose T, Belo Neto LA (2013) Leaf beetles of the Cayman Islands (Coleoptera: Chrysomelidae). Insecta Mundi 279: 1-41. http://centerforsystematicentomology.org/insectamundi/PDF-download.asp?FileName=0279Clarketal.pdf

Clark SM, Rattu A, Cillo D (2014) *Monoxia obesula* Blake, 1939, a species native to the U.S.A. and adventive to Sardinia, Italy (Coleoptera: Chrysomelidae: Galerucinae: Galerucini). Zootaxa 3774: 83-89. doi: 10.11646/zootaxa.3774.1.6.

Csiki E (1940) Bogarak. Coleopteren. In: Teleki P, Csiki E (Eds) Csiki Ernő állattani kutatásai Albániában. Explorationes zoologicae ab E. Csiki in Albania peracte. A Magyar Tudományos Akadémia Balkán-kutatásainak tudományos eredményei. Vol 2. E. Csiki, Budapest, 208-288.

Dalstein V, Schulze M, Wagner T (2016) A further new species of *Afrocrania* Hincks, 1949 from Western and Central Africa (Coleoptera: Chrysomelidae, Galerucinae). Entomologische Zeitschrift 126: 165-168.

Dejean PFAM (1836) Catalogue des coléoptères de la collection de M. le Comte Dejean. Deuxième édition. Paris, Méquignon-Marvis Père et Fils, 442 pp.

Derunkov A, Konstantinov AS (2013) Taxonomic changes in the genus *Diabrotica* Chevrolat (Coleoptera: Chrysomelidae: Galerucinae): results of a synopsis of North and Central America Diabrotica species. Zootaxa 3686: 301-325. doi: 10.11646/zootaxa.3686.3.1.

Derunkov A, Konstantinov AS, Tishechkin A (2013) *Diabrotica lopatini*, a new species (Coleoptera: Chrysomelidae: Galerucinae) from Central America. Caucasian Entomological Bulletin 9: 116-117. http://www.ssc-ras.ru/files/files/21_%20Derunkov.pdf.

Derunkov A, Prado LR, Tishechkin AK, Konstantinov AS (2015) New species of *Diabrotica* Chevrolat (Coleoptera: Chrysomelidae: Galerucinae) and a key to *Diabrotica* and related genera: results of a synopsis of North and Central American *Diabrotica* species. Journal of Insect Biodiversity 3: 1-55. http://www.insectbiodiversity.org/index.php/jib/article/view/101/pdf_37.

Döberl M (1995) Eine neue galerucine aus China: *Parexosoma beeneni* sp. n (Col. Chrysomelidae). Acta Coleopterologica 11: 51-53.

Esch S, Wagner T (2009) Redescription of *Candezea nigrotibialis* Jacoby, 1899 from southern Africa, transferred to *Afropachylepta* gen. nov (Coleoptera: Chrysomelidae, Galerucinae). Entomologische Zeitschrift 119: 17-24.

Fogato W (1979) Note sui *Luperus bicolori* italiani e descrizione di *L. leonardii* n. sp (Coleoptera Chrysomelidae). Memorie della Società Entomologica Italiana 57: 46-64.

Fogato W (1981) Note sul genere *Nymphius* Weise (Coleoptera Chrysomelidae). Bollettino della Società Entomologica Italiana 113: 104-112.

Franz H (1974) Die Nordost-Alpen im Spiegel ihrer Landtierwelt. Eine Gebietsmonographie umfassend: Fauna, Faunengeschichte, Lebensbemeinschaften und Beeinflussung der Tiere durch den Menschen. Band IV. Coleoptera 2. Teil, umfassend die Familien Pselaphidae bis Scolytidae. Universitätsverlag Wagner, Insbruck-München, 707 pp.

Freund W, Wagner T (2003) Revision of *Bonesioides* Laboissière, 1925 (Coleoptera; Chrysomelidae; Galerucinae) from continental Africa. Journal of Natural History 37: 1915-1976. doi: 10.1080/00222930110096519.

Furth DG, Suzuki K (1994) Character correlation studies of problematic genera of Alticinae in relation to Galerucinae (Coleoptera: Chrysomelidae). In: Furth DG (Ed) Proceedings of the Third International Symposium on the Chrysomelidae, Beijing 1992. Backhuys Publishers, Leiden, 116-135.

Futuyma DJ (1990) Observations on the taxonomy and natural history of *Ophraella* Wilcox (Coleoptera: Chrysomelidae), with a description of a new species. Journal of the New York Entomological Society 98: 163-186.

Futuyma DJ (1991) A new species of *Ophraella* Wilcox (Coleoptera: Chrysomelidae) from the southeastern United States. Journal of the New York Entomological Society 99: 643-653.

Gahan CJ (1896) On Coleoptera from Aden and Somaliland. Annals and Magazine of Natural History (6)18: 448-461.

Gilbert AJ (2008) A new species of *Phyllobrotica* Chevrolat, 1836 (Coleoptera: Chrysomelidae) from California, USA, with notes on the western United States species. Pan-Pacific Entomologist 84: 269-279.

Gilbert AJ, Andrews FG (1999) Studies on the Chrysomelidae (Coleoptera) of the Baja California Peninsula: a new species of *Scelolyperus* (Galerucinae), with notes on the genus in Baja California. Pan-Pacific Entomologist 75: 8-12.

Gilbert AJ, Clark SM (2007) A new species of *Acalymma* Barber, 1947 (Chrysomelidae: Galerucinae: Luperini), from southeastern Arizona and New Mexico, U.S.A. Pan-Pacific Entomologist 83: 289-295.

Gilbert AJ, Clark SM (2012) *Synetocephalus penrosei* Gilbert & Clark (Chrysomelidae: Galerucinae: Luperini), a new species from California, U.S.A. Pan-Pacific Entomologist 88: 122-129.

Gök A, Aslan EG, Aslan B (2005) *Monolepta anatolica* Bezdek, 1998 (Coleoptera: Chrysomelidae): a new pest on some stone fruit trees (Rosaceae) in Turkey. Entomological News 116: 335-340.

Gök A, Aslan EG, Aslan B (2007) *Xanthogaleruca subcoerulescens* (Weise, 1884) (Coleoptera: Chrysomelidae), a little-known galerucine from Turkey, with a description of the female, additions to the description of the male, and ecological remarks. Entomological News 118: 259-262. doi: 10.3157/0013-872X(2007)118[259:XSWCCA]2.0.CO;2

Gök A, Aslan EG, Sen I, Ayvaz Y (2006) Redescription of *Lochmaea limbata* Pic, 1898 with a new synonym (Coleoptera: Chrysomelidae: Galerucinae). Annales Zoologici 56: 601-604.

Grobbelaar E (1993) A revision of the southern African species of *Megalognatha* Baly (Coleoptera: Chrysomelidae). Entomology Memoir of South Africa Department of Agriculture 86: 1-85.

Grobbelaar E (1995) A revision of the southern African genus *Pseudorupilia* Jacoby (Coleoptera: Chrysomelidae). African Entomology 3: 189-211.

Grobbelaar E (2008) On the identity of *Ootheca bennigseni* Weise, *O. mutabilis* (Schönherr) and *O. meridiana* sp. n (Chrysomelidae: Galerucinae), bean and cowpea pests in the Afrotropical Region. African Entomology 16: 7-22.

Groll E von, Moura LA de (2016) A new species of *Lilophaea* Bechyné (Coleoptera, Chrysomelidae, Galerucinae) with a historical background and a checklist of the genus. Zootaxa 4168: 195-200. doi: 10.11646/zootaxa.4168.1.12.

Hartmann M, Medvedev LN (2003) To the knowledge of Nepalese Chrysomelidae (Coleoptera). Veröffentlichungen des Naturkundemuseums Erfurt 22: 153-183.

Hasenkamp R, Wagner T (2000) Revision of *Afromaculepta* gen. n., a monophyletic group of Afrotropical galerucine leaf beetles (Coleoptera: Chrysomelidae). Insect Systematics & Evolution 31: 3-26. doi: 10.1163/187631200X00282.

Hatch MH (1971) The beetles of the Pacific Northwest. Part V. Rhipiceroidea, Sternoxi, Phytophaga, Rhynchophora, and Lamellicornia. University of Washington Publications in Biology 16: xiv + 662 pp.

Hazmi IR, Wagner T (2010a) Revalidation and revision of *Ochralea* Clark, 1865 (Coleoptera: Chrysomelidae: Galerucinae) from the Oriental region. Zootaxa 2530: 47-59. doi: 10.5281/zenodo.196518.

Hazmi IR, Wagner T (2010b) Revision of *Arcastes* Baly, 1865 from the Oriental region (Coleoptera, Chrysomelidae, Galerucinae). ZooKeys 42: 79-99. doi: 10.3897/zookeys.42.336.

Hazmi IR, Wagner T (2010c) *Rubrarcastes* gen. nov., a new group of Oriental galerucine leaf beetles (Coleoptera: Chrysomelidae, Galerucinae). Entomologische Zeitschrift 120: 85-88.

Hazmi IR, Wagner T (2013) Revision of *Neolepta* Jacoby, 1884 and related Galerucines from the Oriental Region, including descriptions of two new genera (Coleoptera: Chrysomelidae: Galerucinae). Raffles Bulletin of Zoology 61: 73-95. http://lkcnhm.nus.edu.sg/nus/pdf/PUBLICATION/Raffles%20Bulletin%20of%20Zoology/Past%20Volumes/RBZ%2061(1)/61rbz073-095.pdf.

Heller KM (1898) Neue Käfer von Celebes. III. Abhandlungen und Berichte des Konigl. Zoologischen und Anthropologisch-Ethnographischen Museums zu Dresden 7 (3): 1-41, 1 pl.

Heunemann DC, Dalstein V, Schultze M, Wagner T (2015) *Bicolorizea* gen. nov. from tropical Africa (Coleoptera: Chrysomelidae, Galerucinae). Entomologische Zeitschrift 125: 235-246.

Iablokoff-Khnzorian SM (1970) Novye vidy zhestkokrylykh iz Armenii i drugikh chastey SSSR. Zoologicheskiy Sbornik Akademii Nauk Armyanskoy SSR 15: 50-80.

ICZN (1984) Opinion 1273. *Anaspis*, *Luperus*, *Lampyris* and *Clerus* (Insecta, Coeloptera): Determination of authorship and fixation of type species. Bulletin of Zoological Nomenclature 41: 28-31.

ICZN (1993) Opinion 1709. *Diabrotica undecimpunctata* Mannerheim, 1843 and *D. undecimpunctata howardi* Barber, 1947 (Insecta, Coleoptera): specific and subspecific names conserved. Bulletin of Zoological Nomenclature 50: 77-78.

ICZN (1994) Opinion 1754. Histoire abrégée des insectes qui se trouvent aux environs de Paris (Geoffroy, 1762): some generic names conserved (Crustacea, Insecta). Bulletin of Zoological Nomenclature 51: 58-70.

Israelson G (1980) Taxonomical and nomeclatural notes on some Canarian Coleoptera. Vieraea 9: 183-210. http://islandlab.uac.pt/fotos/publicacoes/publicacoes_Vieraea_9_1978-80_Nr1-2_13.pdf.

Jacoby M (1879) Descriptions of new species of phytophagous Coleoptera. Proceedings of the Scientific Meetings of the Zoological Society of London 1879: 773-793.

Jacoby M (1884) Descriptions of new genera and species of phytophagous Coleoptera collected by Dr. B. Hagen at Serdang (East Sumatra). Notes from the Leyden Museum 6: 201-232.

Jacoby M (1886) Descriptions of new genera and species of phytophagous Coleoptera from the Indo-Malayan and Austra-Malayan subregions, contained in the Genoa Civic Museum. Third Part. Annali del Museo Civico di Storia Naturale di Genova 24: 41-121.

Jacoby M (1889) Viaggio di Leonardo Fea in Birmania e regioni vicine. - List of the phytophagous Coleoptera obtained by Signor L. Fea at Burmah and Tenasserim, with descriptions of the new species. Annali del Museo Civico di Storia Naturale di Genova 27: 147-237.

Jacoby M (1891) Descriptions of some new species of phytophagous Coleoptera. Entomologist 24(Suppl.): 62-65.

Jacoby M (1892) Description of the new genera and species of the phytophagous Coleoptera obtained by Sign. L. Fea in Burma. Annali del Museo Civico di Storia Naturale di Genova 32: 869-999.

Jacoby M (1894) Descriptions of new genera and species of phytophagous Coleoptera obtained by W. Doherty in the Malayan Archipelago. Novitates Zoologicae 1: 267-330.

Jacoby M (1895) Descriptions of some new species of phytophagous Coleoptera from the East. Entomologist 1895(Suppl.) (1893-1895): 105-111.

Jiang SQ (1988a) Five new species of the genus *Haplosomoides* (Coleoptera: Chrysomelidae). Sinozoologia 6: 177-182.

Jiang SQ (1988b) A study on the Chinese *Liroetis* (Coleoptera: Chrysomelidae). Sinozoologia 6: 183-198.

Jiang SQ (1988c) Four new species of Galerucinae from Xizang, China (Coleoptera: Chrysomelidae). Acta Zootaxonomica Sinica 13: 392-396.

Jiang SQ (1989) Four new Chinese species of *Japonitata* (Coleoptera: Chrysomelidae, Galerucinae). Acta Entomologica Sinica 32: 221-225.

Jiang SQ (1990a) A new species of the genus *Pseudosepharia* (Coleoptera: Chrysomelidae). Acta Entomologica Sinica 33: 455-456.

Jiang SQ (1990b) New Galerucinae from the Mt. Namjagbarwa region (Coleoptera: Chrysomelidae). Sinozoologia 7: 137-139.

Jiang SQ (1990c) On the Chinese species of the Galerucinae genus *Zangia* (Coleoptera: Chrysomelidae). Sinozoologia 7: 141-144.

Jiang SQ (1991) Six new species of the genus *Pseudadimonia* (Coleoptera: Chrysomelidae). Acta Entomologica Sinica 34: 83-88.

Jiang SQ (1992) Coleoptera: Chrysomelidae – Galerucinae. In: Chen SH (Ed) Insects of Hengduan Mountain Region. Volume I. Science Press, Beijing, 646-674.

Kimoto S (1970a) A list of the Nepalese chrysomelid specimens preserved in Zoologische Sammlung des Bayerischen Staates, München. Khumbu Himal 3: 412-421.

Kimoto S (1970b) Notes on the Chrysomelidae from Taiwan V. Kontyû 38: 292-313.

Kimoto S (1974a) Notes on the Chrysomelidae from Taiwan VII. Entomological Review of Japan 26: 21-26. http://coleoptera.sakura.ne.jp/ERJ/ERJ26-1974.pdf.

Kimoto S (1974b) New or little known Chrysomelidae (Coleoptera) from Japan and its adjacent regions I. Kontyû 42: 144-150.

Kimoto S (1976) Notes on the Chrysomelidae from Taiwan. VIII. Entomological Review of Japan 29: 1-9. http://coleoptera.sakura.ne.jp/ERJ/ERJ29-1976.pdf.

Kimoto S (1977) Ergebnisse der Bhutan-Expedition 1972 des Naturhistorischen Museums in Basel. Coleoptera: fam Chrysomelidae subfam Galerucinae. Entomologica Basiliensia 2: 351-392.

Kimoto S (1979) The Galerucinae (Coleoptera: Chrysomelidae) of Nepal, Bhutan and Northern Territories of India, in the Natural History Museum in Basel, I. Entomologica Basiliensia 4: 463-478.

Kimoto S (1981a) Revisional study on the Japanese species of genus *Medythia* Jacoby (Col., Chrysomelidae, Galerucinae). Entomological Review of Japan 35: 7-11. http://coleoptera.sakura.ne.jp/ERJ/ERJ35-1981.pdf.

Kimoto S (1981b) Notes on the Chrysomelidae from Taiwan, China. X. Entomological Review of Japan 36: 1-4. http://coleoptera.sakura.ne.jp/ERJ/ERJ36(1)1981.pdf.

Kimoto S (1982a) The Galerucinae of Nepal, Bhutan and Northern Territories of India, in the Natural History Museum in Basel, II (Coleoptera: Chrysomelidae). Entomological Review of Japan 37: 7-24. http://coleoptera.sakura.ne.jp/ERJ/ERJ37(1)1982.pdf.

Kimoto S (1982b) Description of a new species of galerucid beetle from Taiwan, China (Coleoptera: Chrysomelidae). In: Satô M, Hori Y, Arita Y, Okadome T (Eds) Special Issue to the Memory of Retirement of Emeritus Professor Michio Chûjô. Nagoya, 151-152.

Kimoto S (1983) New and little known Chrysomelidae (Coleoptera) from Japan and its adjacent regions, III. Entomological Review of Japan 38: 45-54.

Kimoto S (1984) Notes on the Chrysomelidae from Taiwan, China, XI. Entomological Review of Japan 39: 39-58. http://coleoptera.sakura.ne.jp/ERJ/ERJ39(1)1984.pdf

Kimoto S (1986) New or little known Chrysomelidae (Coleoptera) from Japan and its adjacent regions, IV. In: Ueno S (Ed) Entomological papers presented to Yoshihiko Kurosawa on the occasion of his retirement. Coleopterists' Association of Japan, 309-313. http://coleoptera.sakura.ne.jp/special-publication/Satonius.pdf.

Kimoto S (1989a) Chrysomelidae (Coleoptera) of Thailand, Cambodia, Laos and Vietnam. IV. Galerucinae. Esakia 27: 1-241. http://catalog.lib.kyushu-u.ac.jp/handle/2324/2511/1.pdf.

Kimoto S (1989b) Descriptions of a new genus and three new species of Taiwanese Chrysomelidae (Coleoptera) collected by Dr. Kintaro Baba, on the occasion of his entomological survey in 1986. Entomological Review of Japan 44: 73-78. http://coleoptera.sakura.ne.jp/ERJ/ERJ44(2)1989.pdf.

Kimoto S (1989c) The Taiwanese Chrysomelidae (Insecta: Coleoptera) collected by Dr. Kintari Baba, on the occasion of his entomological survey in 1983 and 1986. Kurume University Journal 38: 237-272.

Kimoto S (1990) Check-list of Chrysomelidae of south east Asia, south of Thailand and west of Irian-Jaya of Indonesia, VI. Galerucinae 2. Kurume University Journal 39: 201-237.

Kimoto S (1991a) Descriptions of a new genus and six new species of Chrysomelidae (Coleoptera) collected by Dr. Kintaro Baba in Taiwan, China. Entomological Review of Japan 46: 13-20. http://coleoptera.sakura.ne.jp/ERJ/ERJ46(1)1991.pdf.

Kimoto S (1991b) Notes on the Chrysomelidae from Taiwan, China, XII. Entomological Review of Japan 46: 115-124. http://coleoptera.sakura.ne.jp/ERJ/ERJ46(2)1991.pdf.

Kimoto S (1994) Description of a new galerucid species from Taiwan, China (Coleoptera: Chrysomelidae). Transactions of the Shikoku Entomological Society 20: 191-192.

Kimoto S (1996) Notes on the Chrysomelidae from Taiwan, China, XIII. Entomological Review of Japan 51: 27-51. http://coleoptera.sakura.ne.jp/ERJ/ERJ51(1)1996.pdf.

Kimoto S (2000a) Descriptions of some new genera and species of Chrysomelidae (Coleoptera) from Thailand, Laos and Vietnam. Serangga 5: 1-39.

Kimoto S (2000b) Chrysomelidae (Coleoptera) of Thailand, Cambodia, Laos and Vietnam. VII. Alticinae. Bulletin of the Institute of Comparative Studies of International Cultures and Societies 26: 103-299.

Kimoto S (2001) The Chrysomelidae (Insecta: Coleoptera) collected by the Kyushu University Scientific Expedition to the Nepal Himalaya in 1971 and 1972. Bulletin of the Kitakyushu Museum of Natural History 20: 17-80.

Kimoto S (2003) The Chrysomelidae (Insecta: Coleoptera) collected by Dr. Akio Otake, on the occasion of his entomological survey in Sri Lanka from 1973 to 1975. Bulletin of the Kitakyushu Museum of Natural History and Human History, Series A Natural History 1: 23-43.

Kimoto S (2004) New or little known Chrysomelidae (Coleoptera) from Nepal, Bhutan and the northern territories of Indian subcontinent. Bulletin of the Kitakyushu Museum of Natural History and Human History, Series A Natural History 2: 47-63.

Kimoto S (2005) Systematic catalogue of the Chrysomelidae (Coleoptera) from Nepal and Bhutan. Bulletin of the Kitakyushu Museum of Natural History and Human History Series A Natural History 3: 13-144.

Kimoto S, Chu YI (1996) Systematic catalog of Chrysomelidae of Taiwan (Insecta: Coleoptera). Bulletin of the Institute of Comparative Studies of International Cultures and Societies 16: 1-152.

Kimoto S, Takahashi Y (1992) Description of a new species of Galerucinae (Chrysomelidae, Col.) from Japan. Entomological Review of Japan 47: 99-101. http://coleoptera.sakura.ne.jp/ERJ/ERJ47(2)1992.pdf.

Kimoto S, Takizawa H (1972) Chrysomelid-beetles of Nepal, collected by the Hokkaido University Scientific Expedition to Nepal Himalaya, 1968. Part I. Kontyû 40: 215-223.

Kimoto S, Takizawa H (1981) Chrysomelid beetles of Nepal, collected by the Hokkaido University Scientific Expeditions to Nepal Himalaya, 1968 and 1975. Part III (Coleoptera). Entomological Review of Japan 35: 51-65. http://coleoptera.sakura.ne.jp/ERJ/ERJ35-1981.pdf.

Kimoto S, Takizawa H (1983) Chrysomelid beetles of Nepal, collected by the Himalaya Expedition 1979 of the National Science Museum, Tokyo (Part 1). Bulletin of the National Science Museum, Tokyo (A) 9: 83-96.

Kizub IV (2016) Notes on Oriental Galerucinae Latreille, 1802 with description of a new species of the genus *Palpoxena* Baly, 1861 (Coleoptera: Chrysomelidae). Munis Entomology & Zoology 11: 18-25. http://www.munisentzool.org/yayin/vol11/issue1/vol11issue1-6882898.pdf.

Komiya Y (2005) Description of a new species of the genus *Galerucella* (Coleoptera, Chrysomelidae, Galerucinae) from Hokkaido, Northern Japan. Elytra 33: 95-99. http://coleoptera.sakura.ne.jp/Elytra/Elytra33(1)2005.pdf.

Konstantinov AS, Lingafelter S (2002) Revision of the Oriental species of *Aphthona* Chevrolat (Coleoptera: Chrysomelidae). The Entomological Society of Washington, Washington, 349 pp.

Kortenhaus S, Wagner T (2010) Revision of *Ootheca* Chevrolat, 1837 from tropical Africa - redescriptions, descriptions of new species and identification key (Coleoptera: Chrysomelidae, Galerucinae). Zootaxa 2659: 1-52. doi: 10.5281/zenodo.276235.

Kortenhaus S, Wagner T (2011) *Oothecoides* gen.nov. from tropical Africa, with redescription and description of six species (Coleoptera: Chrysomelidae, Galerucinae). Entomologische Zeitschrift 121: 259-269.

Kortenhaus S, Wagner T (2012) Description of *Ootibia* gen. n. from tropical Africa with revision of two described species and description of three new species (Coleoptera: Chrysomelidae: Galerucinae). African Entomology 20: 350–370. doi: 10.4001/003.020.0210.

Kortenhaus S, Wagner T (2013) *Oosagitta* gen. nov. from tropical Africa, with revision of two species and description of four new species (Coleoptera: Chrysomelidae, Galerucinae). European Journal of Taxonomy 58: 1-24. doi: 10.5852/ejt.2013.58.

Krysan JL, Branson TF, Schroeder RFW, Steiner WE Jr (1984) Elevation of *Diabrotica sicuanica* (Coleoptera: Chrysomelidae) to the species level with notes on the altitudinal distribution of *Diabrotica* species in the Cuzco Department of Peru. Entomological News 95: 91-98.

Krysan JL, Smith RF (1987a) Systematics of the virgifera species group of *Diabrotica* (Coleoptera: Chrysomelidae: Galerucinae). Entomography 5: 375-484.

Krysan JL, Smith RF (1987b) Three new species of *Diabrotica* (Coleoptera: Chrysomelidae). Entomography 5: 523-530.

Krysan JL, Smith RF, Branson TF, Guss PL (1980) A new subspecies of *Diabrotica* *virgifera* (Coleoptera: Chrysomelidae): description, distribution and sexual compatibility. Annals of the Entomological Society of America 73: 123-130. doi: 10.1093/aesa/73.2.123.

Krysan JL, Smith RF, Guss PL (1983) *Diabrotica barberi* (Coleoptera: Chrysomelidae) elevated to species rank based on behavior, habitat choice, morphometrics, and geographical variation of color. Annals of the Entomological Society of America 76: 197-204. doi: 10.1093/aesa/76.2.197.

Laboissière V (1936a) Galerucinae africains de la Collection du Musée Civique de Gênes. Annales de l’Association des Naturalistes de Levallois-Perret 22(1935-1936): 139-184.

Laboissière V (1936b) Observations sur les Galerucini asiatiques principalement du Tonkin et du Yunnan et descriptions de nouveaux genres et espèces (5e partie). Annales de la Société Entomologique de France 105: 239-261.

Laboissière V (1940) Galerucinae (Coleoptera Phytophaga) Fam. Chrysomelidae. Exploration du Parc National Albert, Mission G. F. de Witte (1933-1935) 31: 93 pp.

Lee CF (2009) A taxonomic revision of M*onolepta pallidula* species group in Taiwan (Coleoptera: Chrysomelidae: Galerucinae). Zootaxa 2170: 15-27. doi: 10.5281/zenodo.189193.

Lee CF (2011) Taxonomic status of *Cneoranidea signatipes* (sensu Kimoto, 1976) (Coleoptera: Chrysomelidae: Galerucinae) in Taiwan. Japanese Journal of Systematic Entomology 17: 351-354.

Lee CF (2014a) Taxonomic notes on the genus *Cerophysa* Chevrolat in Japan and Taiwan (Coleoptera: Chrysomelidae: Galerucinae). Japanese Journal of Systematic Entomology 20: 91-94.

Lee CF (2014b) The genus *Sphenoraia* Clark, 1865 (Coleoptera: Chrysomelidae: Galerucinae) in Taiwan, with description of a new species. Coleopterists Bulletin 68: 143-151. doi: 10.1649/0010-065X-68.1.143.

Lee CF (2015) The genus *Paraplotes* Laboissière, 1933 in Taiwan, a speciose group with brachelytrous females (Coleoptera: Chrysomelidae: Galerucinae). Zootaxa 3904: 223-248. doi: 10.11646/zootaxa.3904.2.3.

Lee CF (2016a) Review of the genus *Siemssenius* Weise, 1922 (Coleoptera: Chrysomelidae: Galerucinae) from Taiwan, with descriptions of five new species. Zootaxa 4158: 367-384. doi: 10.11646/zootaxa.4158.3.4.

Lee CF (2016b) Redescription of *Monolepta sexlineata* Chûjô, 1938 (Coleoptera: Chrysomelidae: Galerucinae). Journal of Taiwan Agricultural Research 65: 1-7. http://ir.tari.gov.tw:8080/bitstream/345210000/7098/2/65-1-1.pdf

Lee CF, Beenen R (2009) The identity of *Neohylaspes rufofulva* Chûjô, 1962 (Coleoptera: Chrysomelidae Galerucinae). Genus 20: 105-107. http://www.biol.uni.wroc.pl/cassidae/Lee_Neohylaspes%20rufofulva.pdf

Lee CF, Beenen R (2012) *Calomicrus jungchangi* Lee and Beenen (Coleoptera: Chrysomelidae: Galerucinae), a new species from Taiwan, with redescription of a similar species, *Monolepta rufofulva* Chûjô, 1938. Coleopterists Bulletin 66: 123-130. doi: 10.1649/072.066.0207.

Lee CF, Beenen R (2015a) Revision of the genus *Aulacophora* from Taiwan (Coleoptera: Chrysomelidae: Galerucinae). Zootaxa 3949: 151-190. doi: 10.11646/zootaxa.3949.2.1.

Lee CF, Beenen R (2015b) Revision of *Taiwanaenidea* Kimoto, 1984 (Coleoptera: Chrysomelidae: Galerucinae). Zootaxa 4020: 153-168. doi: 10.11646/zootaxa.4020.1.6.

Lee CF, Beenen R, Staines CL (2009) Notes on the genus *Cerophysa* Chevrolat in Taiwan (Coleoptera: Chrysomelidae: Galerucinae). Coleopterists Bulletin 63: 456-466. doi: 10.1649/1159.1.

Lee CF, Bezděk J (2012) Taxonomic status of *Cneorella spuria* (sensu Kimoto, 1969) (Coleoptera, Chrysomelidae, Galerucinae) in Taiwan. Japanese Journal of Systematic Entomology 18: 347-353.

Lee CF, Bezděk J (2013a) Revision of *Gallerucida singularis* species group (Coleoptera: Chrysomelidae: Galerucinae). Zootaxa 3647: 358-370. doi: 10.11646/zootaxa.3647.2.7.

Lee CF, Bezděk J (2013b) Revision of the genus *Dercetina* from Taiwan and their similar species, with description of a new species from Myanmar (Insecta, Chrysomelidae, Galerucinae). ZooKeys 323: 1-33. doi: 10.3897/zookeys.323.5195.

Lee CF, Bezděk J (2013c) The genus *Cneorane* Baly, 1865 from Taiwan (Coleoptera: Chrysomelidae: Galerucinae), with notes on sexual dimorphism and its life history. Zoological Studies 52: 1-14. doi: 10.1186/1810-522X-52-9.

Lee CF, Bezděk J (2014a) Revision of the genus *Paridea* Baly, 1886 from Taiwan (Coleoptera, Chrysomelidae, Galerucinae). ZooKeys 405: 83-125. doi: 10.3897/zookeys.405.7458.

Lee CF, Bezděk J (2014b) Redescription of *Meristoides grandipennis* (Coleoptera: Chrysomelidae: Galerucinae). Japanese Journal of Systematic Entomology 20: 189-192.

Lee CF, Bezděk J (2014c) Taxonomic studies on the genus *Apophylia* from Taiwan (Coleoptera: Chrysomelidae: Galerucinae). Journal of Taiwan Agricultural Research 63: 1-16. http://ir.tari.gov.tw:8080/bitstream/345210000/6249/3/63-1-1.pdf.

Lee CF, Bezděk J (2015) Revision of "*Phyllobrotica*" from Taiwan with description of *Jolibrotica* gen. n (Coleoptera, Chrysomelidae, Galerucinae). ZooKeys 547: 75-92. doi: 10.3897/zookeys.547.9381.

Lee CF, Bezděk J (2016a) Revision of the wingless *Sikkimia* Duvivier (Coleoptera, Chrysomelidae, Galerucinae) from Taiwan, including a new generic synonymy and four new species descriptions. ZooKeys 553: 79-106. doi: 10.3897/zookeys.553.6576.

Lee CF, Bezděk J (2016b) Revision of the genus *Morphosphaera* Baly (Coleoptera: Chrysomelidae: Galerucinae). Zootaxa 4179: 1-41. doi: 10.11646/zootaxa.4179.1.1.

Lee CF, Bezděk J (2016c) A remarkable new species of the genus *Cassena* Weise from Vietnam (Coleoptera: Chrysomelidae: Galerucinae). Japanese Journal of Systematic Entomology 22: 87-89.

Lee CF, Bezděk J, Staines CL (2010) A review of the genus *Agetocera* (Coleoptera: Chrysomelidae: Galerucinae) in Taiwan - are there only two species? Zootaxa 2441: 1-19. doi: 10.5281/zenodo.194971.

Lee CF, Bezděk J, Staines CL (2011) A review of the genus *Haplosomoides* Duvivier, 1890 in Taiwan and Japan (Coleoptera: Chrysomelidae: Galerucinae). Zoological Studies 50: 118-138. http://zoolstud.sinica.edu.tw/Journals/50.1/118.pdf.

Lee CF, Bezděk J, Suenaga H (2012) Revision of *Menippus* (Coleoptera: Chrysomelidae: Galerucinae) of Taiwan and *Menippus dimidiaticornis* species group with a new generic synonymy. Zootaxa 3427: 1-16. doi: 10.5281/zenodo.210575.

Lee CF, Staines CL (2010) *Monolepta ongi*, a new species from Lanyu Island, with redescription of its allied species *Monolepta longitarsoides* Chûjô, 1938 (Coleoptera: Chrysomelidae: Galerucinae). Proceedings of the Entomological Society of Washington 112: 530-540. doi: 10.4289/0013-8797.112.4.530.

Lee CF, Tian PL, Staines CL (2010) *Monolepta meihuai*, a new species from Taiwan, with redescription of its sibling species *Monolepta mandibularis* Chûjô, 1962 (Coleoptera: Chrysomelidae: Galerucinae). Coleopterists Bulletin 64: 303-311. doi: 10.1649/0010-065X-64.4.303.

LeSage L (1986) A taxonomic monograph of the Nearctic galerucine genus *Ophraella* Wilcox (Coleoptera: Chrysomelidae). Memoirs of the Entomological Society of Canada 133: 3-75. doi: 10.4039/entm118133fv.

Li JK, Wang ZG (1993) The soil beetles in Anhui province. In: Li JK, Chen P (Eds) Studies on fauna and ecogeography on soil animal. Northeast Normal University, Shengyang, 151-167 (in Chinese).

Li WZ, Yang XK (2002) Chrysomelidae: Galerucinae. In: Li Z, Jin D (Eds) Insect fauna from National Nature Reserve of Guizhou Province, I. Insects from Maolan landscape. Guizhou Science and Technology Publishing House, Guiyang, 299-309.

Lohse GA (1989) Hydrogaleruca-Studien (Col. Chrysomelidae, Gattung Galerucella Crotch). Entomologische Blätter 85: 61-69.

Lopatin IK (1975) Novye vidy listoedov iz yuzhnogo Tadzhikistana (Coleoptera: Chrysomelidae). Izvestiya Akademii Nauk Tadzhikskoy SSR (Otdelenie Biologicheskikh Nauk) 3(60): 45-51.

Lopatin IK (1979) Neue und wenig bekannte Chrysomeliden (Coleoptera) von Nord-Indien aus der Sammlung des Naturhistorischen Museums Basel. Entomologica Basiliensia 4: 431-441.

Lopatin IK (1981) New genera and species of leaf-beetles (Coleoptera, Chrysomelidae) from Iran. Results of the Czechoslovak-Iranian expedition in 1973. II. Entomologicheskoe Obozrenie 60: 623-628.

Lopatin IK (1983) New species of the leaf-beetles (Coleoptera, Chrysomelidae) from Middle Asia and South-eastern Kazakhstan. Entomologicheskoe Obozrenie 62: 91-95.

Lopatin IK (1984) Leaf-beetles (Coleoptera, Chrysomelidae) of Iran. Results of the Czechoslovak-Iranian expeditions in 1973-1977. III. Entomologicheskoe Obozrenie 63: 79-93.

Lopatin IK (1988) Calomicrus ghilarovi sp. n. i blizkie k nemu vidy iz Sredney Azii i Irana (Coleoptera, Chrysomelidae). Trudy Vsesoyuznogo Entomologicheskogo Obshchestva 70: 65-67.

Lopatin IK (1990) Novye i maloizvestnie vidy zhukov-listoedov (Coleoptera, Chrysomelidae) fauny SSSR. In: Novosti faunistiki i sistematiki. Naukova Dumka, Kiev, 48-54.

Lopatin IK (1997) New species of chrysomelid beetles (Coleoptera, Chrysomelidae) of the Asiatic fauna. Entomologicheskoe Obozrenie 76: 363-377.

Lopatin IK (2001) Beitrag zur Kenntnis der Chrysomeliden des Jemen (Coleoptera: Chrysomelidae). Mitteilungen des Internationalen Entomologischen Vereins e. V (Frankfurt a. Main) 26: 13-19.

Lopatin IK (2002a) New data on the taxonomy and distribution of leaf beetles in Israel (Coleoptera: Chrysomelidae). Zoosystematica Rossica 10: 379-380.

Lopatin IK (2002b) New species of leaf-beetles (Coleoptera, Chrysomelidae) from China. II. Entomologicheskoe Obozrenie 81: 874-882.

Lopatin IK (2002c) New data on the leaf-beetles of the South and East Asia (Coleoptera, Chrysomelidae). Descriptions and synonymic remarks. Euroasian Entomological Journal 1: 83-86.

Lopatin IK (2003a) A new genus and its two new species of leaf-beetles from Vietnam (Coleoptera: Chrysomelidae: Galerucinae). Genus 14: 103-107. http://www.biol.uni.wroc.pl/cassidae/Vietocerus.pdf

Lopatin IK (2003b) New species of leaf-beetles from South-East Asia (Coleoptera, Chrysomelidae). Euroasian Entomological Journal 2: 301-304.

Lopatin IK (2004a) New species of leaf beetles (Coleoptera, Chrysomelidae) from China. III. Entomologicheskoe Obozrenie 83: 614-621.

Lopatin IK (2004b) New species of leaf-beetles (Coleoptera, Chrysomelidae) from China and Vietnam. Euroasian Entomological Journal 3: 187-191.

Lopatin IK (2005a) Three new species of leaf beetles (Coleoptera, Chrysomelidae) from Nepal, descriptions and synonymies. Euroasian Entomological Journal 4: 231-232.

Lopatin IK (2005b) New species of leaf beetles (Coleoptera, Chrysomelidae) from China. IV. Entomologicheskoe Obozrenie 84: 569-575.

Lopatin IK (2005c) New species of leaf-beetles (Coleoptera, Chrysomelidae) from China. V. Entomologicheskoe Obozrenie 84: 873-880.

Lopatin IK (2006a) *Hyphaenia medvedevi* sp. n. (Coleoptera: Chrysomelidae: Galerucinae), a new leaf-beetle species from China. Trudy Russkogo Entomologicheskogo Obshchestva 77: 211-212. https://www.zin.ru/animalia/coleoptera/pdf/lopatin_horae_77.pdf.

Lopatin IK (2006b) New species of leaf beetles (Coleoptera, Chrysomelidae) from China. VI. Entomologicheskoe Obozrenie 85: 593-601, 715.

Lopatin IK (2006c) A new species of *Calomicrus* from the United Arabian Emirates (Coleoptera: Chrysomelidae: Galerucinae). Zoosystematica Rossica 14: 261-262. https://www.zin.ru/journals/zsr/content/2005/zr_2005_14_2_Lopatin.pdf.

Lopatin IK (2007) New species of the leaf beetles (Coleoptera, Chrysomelidae) from China. VII. Entomologicheskoe Obozrenie 86: 176-184.

Lopatin IK (2008a) A new replacement name for junior homonym *Sichuania* Lopatin, 2002 (Coleoptera: Chrysomelidae: Galerucinae). Zoosystematica Rossica 17: 138. https://www.zin.ru/journals/zsr/content/2008/zr_2008_17_1_Lopatin_1.pdf

Lopatin IK (2008b) New species of the leaf beetles (Coleoptera, Chrysomelidae) from China. IX. Entomologicheskoe Obozrenie 87: 831-841.

Lopatin IK (2008c) *Sinoluperus vietnamicus* sp. n. - the first representative of the Chinese genera in Vietnam (Coleoptera: Chrysomelidae: Galerucinae). Zoosystematica Rossica 17: 150. https://www.zin.ru/journals/zsr/content/2008/zr_2008_17_1_Lopatin_2.pdf.

Lopatin IK (2011a) New genus and species of leaf-beetles (Coleoptera, Chrysomelidae, Galerucinae) from China. Euroasian Entomological Journal 10: 143-144.

Lopatin IK (2011b) New species of leaf-beetles (Coleoptera, Chrysomelidae) from China. X. Entomologicheskoe Obozrenie 90: 375-387.

Lopatin IK (2013) New species of leaf-beetles (Coleoptera, Chrysomelidae) from China. XI. Entomologicheskoe Obozrenie 92: 765-776.

Lopatin IK, Konstantinov AS (2009) New genera and species of leaf beetles (Coleoptera: Chrysomelidae) from China and South Korea. Zootaxa 2083: 1-18.

Lopatin IK, Nesterova O (2006) A new species of the genus *Calomicrus* Stephens, 1832 from United Arab Emirates (Coleoptera: Chrysomelidae: Galerucinae). Genus 17: 537-539. http://www.biol.uni.wroc.pl/cassidae/Lopatin_Calomicrus%20arabicus.pdf

Lopatin IK, Nesterova O (2013) Two new species of the genus *Calomicrus* Dillwyn, 1829 (Coleoptera: Chrysomelidae: Galerucinae) from Turkey. Caucasian Entomological Bulletin 9: 95-96. http://www.ssc-ras.ru/files/files/15_%20Lopatin.pdf.

Makhan D (2012) *Galerucella rishwani* sp. nov. (Coleoptera: Chrysomelidae), a new leaf beetle from Chal Godarzi, Borujerd Lorestan Province, Iran. Calodema 230: 1-4.

Mandl K (1970) Revision der Gattung *Theone* Gistl (Coleoptera - Chrysomelidae - Galerucinae). 2. Teil. Entomologische Arbeiten aus dem Museum G. Frey 21: 154-169. http://www.zobodat.at/pdf/Entomologische-Arbeiten-Museum-Frey_21_0154-0169.pdf

Mandl K (1974) Eine neue Art aus der Gattung *Pseudadimonia* Duvivier (Col. Chrysomelidae). Entomologische Arbeiten aus dem Museum G. Frey 25: 316-318. http://www.zobodat.at/pdf/Entomologische-Arbeiten-Museum-Frey_25_0316-0318.pdf.

Mandl K (1976) Beschreibung des Männchens von *Pallasiola theoneiformis* Mandl (Chrysomelidae: Galerucinae). Entomologische Arbeiten aus dem Museum G. Frey 27: 398-399. http://www.zobodat.at/pdf/Entomologische-Arbeiten-Museum-Frey_27_0398-0399.pdf.

Mandl K (1981) Neue Coleopteren-Taxa vom Nahen bis zum Fernen Osten. Entomologica Basiliensia 6: 167-182.

Mandl K (1986) *Pseudadimonia holzschuhi*, eine neue Galerucinae-Art aus Nepal, und Bemerkungen zu einer vergessene Art: Rhabdotilla rosti Jakobson 1911 (Chrysomelidae Col.). Mittleilungen der Entomologischen Gesellschaft Basel 36: 71-77.

Medvedev LN (1972) Chrysomelidae from Ceylon (Col.). Entomologische Arbeiten aus dem Museum G. Frey 23: 178-185. http://www.zobodat.at/pdf/Entomologische-Arbeiten-Museum-Frey_23_0178-0185.pdf.

Medvedev LN (1973) New leaf-beetles (Coleoptera, Chrysomelidae) from Palaearctic. Entomologicheskoe Obozrenie 52: 876-885.

Medvedev LN (1974) Contribution to the knowledge of Chrysomelidae (Coleoptera) from Ceylon. Revue Suisse de Zoologie 81: 797-802.

Medvedev LN (1975) Chrysomelidae collected by Dr. W. Wittmer in Turkey and Iran. Mitteilungen aus der Entomologischen Gesellschaft Basel (N. F.) 21: 12-19.

Medvedev LN (1978) Taksonomicheskie zametki o zhukakh-listoedakh (Coleoptera, Chrysomelidae) Sakhalina i Kurilskih ostrovov. Trudy Biologo-Pochvennogo Instituta (N. S.) 50: 82-86.

Medvedev LN (1979) New forms of leaf beetles (Coleoptera, Chrysomelidae) of Oceania. Zoologicheskii Zhurnal 58: 1741-1744.

Medvedev LN (1981) A new species of the genus *Agetocera* (Coleoptera, Chrysomelidae) from Vietnam. Zoologicheskii Zhurnal 60: 617-620.

Medvedev LN (1985) On the fauna of leaf-beetles (Coleoptera, Chrysomelidae) of Afghanistan. II. Entomologicheskoe Obozrenie 64: 370-377.

Medvedev LN (1990) Chrysomelidae from the Nepal Himalayas, II (Insecta: Coleoptera). Stuttgarter Beiträge zur Naturkunde, Serie A (Biologie) 453: 1-46.

Medvedev LN (1992a) Novye i maloizvestnye vidy listoedov (Coleoptera, Chrysomelidae) s ostrovov *Vietnamskogo poberezhia*. In: Medvedev LN (Ed) Sistematika i ekologiya nasekomykh Vietnama. Nauka, Moskva, 71-77.

Medvedev LN (1992b) Chrysomelidae from the Nepal Himalayas, III (Insecta: Coleoptera). Stuttgarter Beiträge zur Naturkunde, Serie A (Biologie) 485: 1-36.

Medvedev LN (1993) New species of Chrysomelidae from South Asia from the Natural History Museum in Basel. Entomologica Basiliensia 16: 359-376.

Medvedev LN (1995) Chrysomelidae (Coleoptera) from Leyte Island, Philippines. Stuttgarter Beiträge zur Naturkunde, Serie A (Biologie) 526: 1-22.

Medvedev LN (1996) The Chrysomelidae of Arabia. Fauna of Saudi Arabia 15: 211-263.

Medvedev LN (1997a) New Alticinae and Galerucinae (Coleoptera, Chrysomelidae) from South Asia. Acta Zoologica Academiae Scientiarum Hungaricae 43: 207-211.

Medvedev LN (1997b) New and interesting Chrysomelidae (Coleoptera) from the Philippines collected by Dr. M.Satô. Japanese Journal of Systematic Entomology 3: 99-104.

Medvedev LN (1998a) A new species of the genus *Luperus* (Coleoptera, Chrysomelidae) from the Far East. Zoologicheskiy Zhurnal 77: 613-614.

Medvedev LN (1998b) New Chrysomelidae (Coleoptera) from southeast Asia in the Hungarian Natural History Museum. Annales Historico-Naturales Musei Nationalis Hungarici 90: 163-174. http://publication.nhmus.hu/pdf/annHNHM/Annals_HNHM_1998_Vol_90_163.pdf.

Medvedev LN (1999) To the knowledge of Chrysomelidae (Coleoptera) from Nepal and adjacent regions. Veröffentlichungen des Naturkundemuseums Erfurt 18: 181-187.

Medvedev LN (2000a) A revision of the genus *Hoplasoma* Jacoby, 1884 (Coleoptera: Chrysomelidae). Russian Entomological Journal 8(1999): 123-128.

Medvedev LN (2000b) To the knowledge of Oriental Chrysomelidae. Russian Entomological Journal 8(1999): 259-264.

Medvedev LN (2000c) Chrysomelidae (Coleoptera) of Laos from the collection of the Hungarian Natural History Museum. Annales Historico-Naturales Musei Nationalis Hungarici 92: 161-182. http://publication.nhmus.hu/pdf/annHNHM/Annals_HNHM_2000_Vol_92_161.pdf.

Medvedev LN (2000d) Chrysomelidae from the Nepal Himalayas, with revision of the genus *Haplosomoides* (Insecta: Coleoptera). Stuttgarter Beiträge zur Naturkunde, Serie A (Biologie) 616: 1-32. http://www.naturkundemuseum-bw.de/sites/default/files/publikationen/serie-a/A616.pdf.

Medvedev LN (2001a) A revision of Oriental *Aulacophora* Chevrolat, 1837 (Chrysomelidae: Galerucinae) with metallic color. Russian Entomological Journal 10: 28-32.

Medvedev LN (2001b) Chrysomelidae of southern Asia (Coleoptera). Entomologica Basiliensia 23: 159-191.

Medvedev LN (2001c) Jacoby´s types of Chrysomelidae (Coleoptera) from Burma in the Museo Civico di Storia Naturale "Giacomo Doria", Genoa. Part 1. Annali del Museo Civico di Storia Naturale "Giacomo Doria" 93(1999-2000): 167-184.

Medvedev LN (2001d) Jacoby´s types of Chrysomelidae (Coleoptera) from Burma in the Museo Civico di Storia Naturale "Giacomo Doria", Genoa. Part 2. Annali del Museo Civico di Storia Naturale "Giacomo Doria" 93(1999-2000): 607-616.

Medvedev LN (2002a) Jacoby´s types of Chrysomelidae (Coleoptera) from Burma in the Museo Civico di Storia Naturale "Giacomo Doria", Genoa. Part 3. Annali del Museo Civico di Storia Naturale "Giacomo Doria" 94: 249-264.

Medvedev LN (2002b) New and poorly known Chrysomelidae (Coleoptera) from northern India. Entomologica Basiliensia 24: 245-253.

Medvedev LN (2002c) A new apterous genus of Galerucinae (Coleoptera: Chrysomelidae) from Sulawesi. Russian Entomological Journal 11: 3-4.

Medvedev LN (2002d) New and poorly known Chrysomelidae from the Philippines (Insecta, Coleoptera). Spixiana 25: 59-67. http://www.zobodat.at/pdf/Spixiana_025_0059-0067.pdf.

Medvedev LN (2003a) New taxa of Chrysomelidae from Afrotropical and Oriental regions (Insecta, Coleoptera). Spixiana 26: 149-153. http://www.zobodat.at/pdf/Spixiana_026_0149-0153.pdf.

Medvedev LN (2003b) New species of leaf beetles from Nepal. In: Hartmann M, Baumbach H (Eds) Biodiversität und Naturausstattung im Himalaya. Verein der Freunde und Förderer des Naturkundesmuseums Erfurt e. V., Erfurt, 317-322.

Medvedev LN (2004a) New and firstly recorded Chrysomelidae (Coleoptera) from Laos. Entomologica Basiliensia 26: 299-323.

Medvedev LN (2004b) New genera and species of Oriental Chrysomelidae (Coleoptera). Entomologica Basiliensia 26: 325-338.

Medvedev LN (2004c) Towards knowledge of the genus *Xenoda* Baly, 1877 (Chrysomelidae, Galerucinae). Entomologica Basiliensia 26: 339-348.

Medvedev LN (2004d) New species of Chrysomelidae (Coleoptera) from Nepal. Veröffentlichungen des Naturkundemuseums Erfurt 23: 203-206.

Medvedev LN (2005a) A revision of the continental Asian species of the genus *Atysa* Baly, 1864 (Chrysomelidae, Galerucinae). Entomologica Basiliensia et Collectionis Frey 27: 227-237.

Medvedev LN (2005b) New and poorly-known genera and species of Oriental Chrysomelidae (Coleoptera). Entomologica Basiliensia et Collectionis Frey 27: 279-295.

Medvedev LN (2005c) Revision of the genus *Monolepta* Chevrolat from the Philippines (Coleoptera, Chrysomelidae). Entomologica Basiliensia 27: 239-277.

Medvedev LN (2007a) New and poorly known Oriental Chrysomelidae (Coleoptera) of the Staatliches Museum für Naturkunde, Stuttgart. Stuttgarter Beiträge zur Naturkunde, Serie A (Biologie) 702: 1-19. http://www.naturkundemuseum-bw.de/sites/default/files/publikationen/serie-a/a_702.pdf.

Medvedev LN (2007b) New species of Oriental Chrysomelidae (Coleoptera). Entomologica Basiliensia 29: 289-305.

Medvedev LN (2007c) New taxa of Oriental Chrysomelidae (Coleoptera). Euroasian Entomological Journal 6: 433-438.

Medvedev LN (2007d) To the knowledge of Chrysomelidae (Coleoptera) described by V. Motschulsky. Russian Entomological Journal 15(2006): 409-417.

Medvedev LN (2008a) New species of Chrysomelidae (Coleoptera) from Sulawesi. Entomologica Basiliensia et Collectionis Frey 30: 243-261.

Medvedev LN (2008b) New and poorly known Chrysomelidae (Coleoptera) from the islands of Bali and Lombok (Indonesia). Stuttgarter Beiträge zur Naturkunde A, Neue Serie 1: 431-434. http://www.naturkundemuseum-bw.de/sites/default/files/publikationen/serie-a/ans01-18medvedev.pdf.

Medvedev LN (2009a) Chrysomelidae (Coleoptera) of high mountain regions of North-West Vietnam. Russian Entomological Journal 18: 201-208. http://kmkjournals.com/upload/PDF/REJ/18/ent18_3_201_208_Medvedev.pdf.

Medvedev LN (2009b) A revision of the genus *Cassena* Weise, 1892 (Coleoptera, Chrysomelidae). Entomologica Basiliensia 31: 219-238.

Medvedev LN (2009c) A revision of the fulvous species of the genus *Mimastra* Baly, 1865 from Vietnam (Chrysomelidae, Galerucinae). Entomologica Basiliensia 31: 255-266.

Medvedev LN (2009d) Taxonomical notes on leaf beetles (Coleoptera, Chrysomelidae). Euroasian Entomological Journal 8: 57-58.

Medvedev LN (2009e) New species of Chrysomelidae from Nepal in the collection of the Naturkundemuseum Erfurt (Insecta: Coleoptera). Vernate 28: 409-412.

Medvedev LN (2009f) New species of leaf beetles from the collection of the Naturkundemuseum Erfurt (Insecta: Coleoptera: Chrysomelidae). In: Hartmann M, Weipert J (Eds) Biodiversität und Naturausstattung im Himalaya III. Verein der Freunde & Förderer des Naturkundemuseums Erfurt e.V., Erfurt, 407-410.

Medvedev LN (2010) New taxa of Chrysomelidae (Coleoptera) collected in canopy trees of South Vietnam. Russian Entomological Journal 19: 241-244. http://kmkjournals.com/upload/PDF/REJ/19/ent19_3_241_244_Medvedev.pdf.

Medvedev LN (2011a) A contribution to knowledge of Oriental species of *Cneorane* Baly, 1865 (Chrysomelidae, Galerucinae). Entomologica Basiliensia et Collectionis Frey 33: 351-368.

Medvedev LN (2011b) On taxonomy and nomenclature of leaf beetle species (Coleoptera, Chrysomelidae) of Russia. Euroasian Entomological Journal 10: 475-478.

Medvedev LN (2011c) New taxa of Chrysomelidae (Coleoptera) from Nepal. Vernate 30: 249-254.

Medvedev LN (2012a) To the knowledge of the genera *Mandarella* Duvivier and Stenoluperus Ogloblin (Insecta: Chrysomelidae: Alticinae) from the Himalayas. In: Hartmann M, Weipert J (Eds) Biodiversität und Naturausstattung im Himalaya IV. Verein der Freunde und Förderer des Naturkundemuseums Erfurt e.V., Erfurt, 423-427.

Medvedev LN (2012b) New species of Chrysomelidae (Coleoptera) from Indochina. Euroasian Entomological Journal 11: 63-69.

Medvedev LN (2012c) New species of *Luperus* Geoffroi, 1762 (Coleoptera: Chrysomelidae: Galerucinae) from China and Vietnam. Caucasian Entomological Bulletin 8: 252-253. http://www.ssc-ras.ru/files/files/10_%20Medvedev.pdf.

Medvedev LN (2012d) New species of leaf-beetles (Chrysomelidae) from Arabia. Russian Entomological Journal 21: 415-418. http://kmkjournals.com/upload/PDF/REJ/21/ent21_4%20415_418%20Medvedev.pdf

Medvedev LN (2012e) New and interesting Chrysomelidae (Insecta: Coleoptera) from the collection of the Naturkundemuseum Erfurt. Vernate 31: 489-503.

Medvedev LN (2013a) New species of *Calomicrus* Stephens, 1834 (Chrysomelidae: Galerucinae) from China and Indochina. Russian Entomological Journal 22: 37-42. http://kmkjournals.com/upload/PDF/REJ/22/ent22_1%20037_042%20Medvedev%20for%20Inet.pdf

Medvedev LN (2013b) New and poorly known Oriental Chrysomelidae (Coleoptera). Acta Biologica Universitatis Daugavpiliensis 13 (2): 85-87.

Medvedev LN (2013c) New and interesting Chrysomelidae (Insecta: Coleoptera) from the collection of the Naturkundemuseum Erfurt. Vernate 32: 415-420.

Medvedev LN (2013d) New taxa of Oriental leaf beetles (Coleoptera: Chrysomelidae). Russian Entomological Journal 22: 293-296. http://kmkjournals.com/upload/PDF/REJ/22/ent22_4%20293_296%20Medvedev.pdf

Medvedev LN (2013e) New species of the chrysomelid genus *Trichomimastra* Weise, 1922 (Coleoptera, Chrysomelidae) from Pakistan. Entomologicheskoe Obozrenie 92: 777-779.

Medvedev LN (2013f) A key for identification with description of new species of the genus *Pyrrhalta* Joannis, 1865 (Coleoptera: Chrysomelidae: Galerucinae) from Indochina. Caucasian Entomological Bulletin 9: 267-272. http://www.ssc-ras.ru/files/files/9_%20Medvedev.pdf.

Medvedev LN (2014a) New and poorly known leaf beetles (Insecta: Coleoptera: Chrysomelidae) from China and Indochina. Vernate 33: 237-241.

Medvedev LN (2014b) Revision of the genus *Paleosepharia* Laboissiere, 1936 (Coleoptera: Chrysomelidae) from Indochina. Russian Entomological Journal 23: 45-51. http://kmkjournals.com/upload/PDF/REJ/23/ent23_1_045_051_Medvedev_for_Inet.pdf

Medvedev LN (2015a) New taxa of Chrysomelidae (Coleoptera) from Vietnam. Russian Entomological Journal 24: 67-72. http://kmkjournals.com/upload/PDF/REJ/24/ent24_1_067_072_Medvedev_for_Inet.pdf.

Medvedev LN (2015b) New and poorly known Oriental Chrysomelidae (Insecta: Coleoptera) in the collection of the Naturkundemuseum Erfurt. Vernate 34: 319-335.

Medvedev LN (2015c) Two new species of Oriental Chrysomelidae (Insecta: Coleoptera) in the collection of the Naturkundemuseum Erfurt. Vernate 34: 336-340.

Medvedev LN (2015d) New and poorly known Chrysomelidae (Insecta: Coleoptera) from the Himalayas. In: Hartmann M, Weipert J (Eds) Biodiversität und Naturausstattung im Himalaya V. Verein der Freunde und Förderer des Naturkundemuseums Erfurt e.V., Erfurt, 475-478.

Medvedev LN (2016) New and poorly known Oriental Chrysomelidae (Insecta: Coleoptera) in the collection of the Naturkundemuseum Erfurt. Vernate 35: 347-365.

Medvedev LN, Beenen R (2010) A contribution to knowledge of Oriental *Strobiderus* Jacoby, 1884 (Chrysomelidae, Galerucinae). Entomologica Basiliensia et Collectionis Frey 32: 289-305.

Medvedev LN, Bezděk J (2002) Two new Oriental genera of Galerucinae (Coleoptera: Chrysomelidae). Entomologische Zeitschrift 112: 9-11.

Medvedev LN, Dang DT (1981) New genera and species of leaf-beetles of the subfamily Galerucinae (Coleoptera, Chrysomelidae) from Vietnam. Entomologicheskoe Obozrenie 60: 629-635.

Medvedev LN, Javorskaya MI (2008) *Monolepta septentrionalis*, a new species from the Russian Far East (Coleoptera, Chrysomelidae, Galerucinae). Euroasian Entomological Journal 7: 284-286.

Medvedev LN, Mirzoeva NB (1969) Novyy vid iz roda *Galeruca* Geoffr (Chrysomelidae, Coleoptera) iz Azerbaydzhana. Doklady Akademii Nauk Azerbaydzhanskoy SSR 25: 56-58.

Medvedev LN, Romantsov P (2012) New and poorly known Chrysomelidae from the Oriental Region (Coleoptera). Entomologische Zeitschrift 122: 75-78.

Medvedev LN, Romantsov P (2013) New and poorly known Chrysomelidae (Coleoptera) from South-East Asia. Caucasian Entomological Bulletin 9: 137-140. http://www.ssc-ras.ru/files/files/25_%20Medvedev.pdf.

Medvedev LN, Romantsov P (2014). New and poorly known Chrysomelidae (Coleoptera) from Borneo. Stuttgarter Beiträge zur Naturkunde A, Neue Serie 7: 235-251. http://www.naturkundemuseum-bw.de/sites/default/files/publikationen/serie-a/ans07-12.pdf

Medvedev LN, Samoderzhenkov E (1989) New Galerucinae from Vietnam (Coleoptera, Chrysomelidae). Entomofauna 10: 453-462. http://www.zobodat.at/pdf/ENT_0010_0453-0462.pdf

Medvedev LN, Samoderzhenkov EV (1998) Revision of *Paridea* Baly, 1886 (Chrysomelidae, Galerucinae) from the Himalaya and adjacent regions. Russian Entomological Journal 6(1997): 57-65.

Medvedev LN, Sprecher-Uebersax E (1997) Chrysomelidae of Nepal and neighbouring regions (Coleoptera: Chrysomelidae). Coleoptera 1: 203-247.

Medvedev LN, Sprecher-Uebersax E (1998) New data on Chrysomelidae of Nepal (Insecta, Coleoptera). Spixiana 21: 25-42. http://www.zobodat.at/pdf/Spixiana_021_0025-0042.pdf

Medvedev LN, Sprecher-Uebersax E (1999) Taxonomical study of Chrysomelidae (Coleoptera) from Nepal. Entomologica Basiliensia 21: 355-370.

Mertgen S, Wagner T (2006) Redescription of *Candezea dahlmani* Jacoby, 1899 from tropical Africa, transferred to *Panafrolepta* n. gen (Coleoptera: Chrysomelidae: Galerucinae). Mitteilungen des Internationalen Entomologischen Vereins E.V. Frankfurt A.M. 31: 11-22.

Middelhauve J, Wagner T (2001) Revision of *Afrocrania*. Part I: Species in which the males have head cavities or extended elytral extrusions. European Journal of Entomology 98: 511-532. doi: 10.14411/eje.2001.066.

Mignot EC (1970) A replacement name for *Malacorhinus tripunctata* (Jacoby) (Galerucinae: Chrysomelidae). Coleopterists Bulletin 24: 59.

Mohamedsaid MS (1990) The genus *Aplosonyx* Chevrolat from Peninsula Malaysia (Coleoptera, Chrysomelidae, Galerucinae). Entomological Review of Japan 45: 37-42. http://coleoptera.sakura.ne.jp/ERJ/ERJ45(1)1990.pdf.

Mohamedsaid MS (1992) The genus *Mimastra* Baly from Peninsular Malaysia (Coleoptera, Chrysomelidae, Galerucinae). Malayan Nature Journal 46: 115-118.

Mohamedsaid MS (1993a) On the genus *Cerophysa* Chevrolat and *Taumacera* Thunberg from Peninsular Malaysia (Coleoptera: Chrysomelidae: Galerucinae). Genus 4: 113-119.

Mohamedsaid MS (1993b) New species of *Monolepta* Chevrolat from Borneo (Coleoptera, Chrysomelidae, Galerucinae). Entomological Review of Japan 48: 1-9. http://coleoptera.sakura.ne.jp/ERJ/ERJ48(1)1993.pdf.

Mohamedsaid MS (1993c) The genus *Coelocrania* and the resurrection of the genus *Pseudosastra* (Coleoptera: Chrysomelidae: Galerucinae). Psyche 100: 223-234. doi: 10.1155/1993/25247.

Mohamedsaid MS (1994a) Two new species of *Coeligetes* Jacoby from Malaysia (Coleoptera, Chrysomelidae, Galerucinae). Psyche 101: 85-92. doi: 10.1155/1994/80931.

Mohamedsaid MS (1994b) A new species of the genus *Metrioidea* from Malaysia (Coleoptera, Chrysomelidae, Galerucinae). Entomological Review of Japan 49: 25-28. http://coleoptera.sakura.ne.jp/ERJ/ERJ49(1)1994.pdf.

Mohamedsaid MS (1994c) The genus *Haplosomoides* Duvivier from Malaysia (Coleoptera, Chrysomelidae, Galerucinae). Entomological Review of Japan 49: 103-107. http://coleoptera.sakura.ne.jp/ERJ/ERJ49(2)1994.pdf.

Mohamedsaid MS (1994d) A new species of *Taumacera* Thunb. from Sarawak, Ma1aysia (Coleoptera: Chrysomelidae: Galerucinae). Genus 5: 169-172.

Mohamedsaid MS (1994e) The genus *Sastroides* Jacoby from Malaysia (Coleoptera: Chrysomelidae: Galerucinae). Sains Malaysiana 23: 29-35.

Mohamedsaid MS (1994f) New species of *Aulacophora* from Sabah, Malaysia (Coleoptera: Chrysomelidae: Galerucinae). Treubia 31: 1-9. doi: 10.14203/treubia.v31i1.567.

Mohamedsaid MS (1994g) A redescription of *Haplosomoides serena* (Boheman) (Coleoptera, Chrysomelidae, Galerucinae). Treubia 31: 47-50. doi: 10.14203/treubia.v31i1.631.

Mohamedsaid MS (1994h) The genus *Aulacophora* Chevrolat from Peninsular Malaysia (Coleoptera: Chrysomelidae: Galerucinae). Malayan Nature Journal 47: 375-392.

Mohamedsaid MS (1995a) New species of leaf beetles from the genus *Itylus* Jacoby (Coleoptera: Chrysomelidae: Galerucinae). Sains Malaysiana 24: 89-95.

Mohamedsaid MS (1995b) The Malaysian species of the genus *Platyxantha* Baly (Coleoptera: Chrysomelidae: Galerucinae). Raffles Bulletin of Zoology 43: 75-82. http://lkcnhm.nus.edu.sg/nus/pdf/PUBLICATION/Raffles%20Bulletin%20of%20Zoology/Past%20Volumes/RBZ%2043(1)/43rbz075-082.pdf

Mohamedsaid MS (1995c) The biodiversity profile of the leaf beetle of the subfamily Galerucinae (Insecta: Coleoptera: Chrysomelidae) from Danum Yalley, Sabah. Wallaceana 74: 1-5.

Mohamedsaid MS (1996a) *Paleosepharia* of Peninsular Malaysia (Coleoptera: Chrysomelidae: Galerucinae). Serangga 1: 7-21.

Mohamedsaid MS (1996b) A new genus and two new species of Galerucinae from Malaysia (Coleoptera: Chrysomelidae). Serangga 1: 79-89.

Mohamedsaid MS (1996c) The galerucine beetles of Java, with description of a new species (Coleoptera: Chrysomelidae: Galerucinae). Stobaeana 7: 1-4.

Mohamedsaid MS (1997a) The Malaysian species of the genus *Palpoxena* Baly (Coleoptera: Chrysomelidae: Galerucinae). Serangga 2: 53-64.

Mohamedsaid MS (1997b) Kinabalua, a new genus of chrysomelid beetle from Sabah, Malaysia (Coleoptera: Chrysomelidae: Galerucinae). Serangga 2: 131-135.

Mohamedsaid MS (1997c) Checklist of the Galerucinae from Taman Negara Lambir, Sarawak (Coleoptera: Chrysomelidae). Serangga 2: 153-175.

Mohamedsaid MS (1997d) The galerucine beetles of Banggi Islands, Sabah (Coleoptera: Chrysomelidae). Serangga 2: 195-207.

Mohamedsaid MS (1997e) An interesting new genus of chrysomelid beetle from Sarawak, Malaysia (Coleoptera: Chrysomelidae: Galerucinae). Genus 8: 621-624.

Mohamedsaid MS (1997f) The galerucine beetles of Sri Lanka, with descriptions of two new species (Coleoptera: Chrysomelidae). Stobaeana 9: 1-7.

Mohamedsaid MS (1998a) *Galeruca malakkana* spec. nov. from Malaysia (Coleoptera: Chrysomelidae: Galerucinae). Zoologische Mededelingen 72: 101-104.

Mohamedsaid MS (1998b) *Borneola*, a new genus of Galerucinae from Malaysia (Coleoptera: Chrysomelidae). Serangga 3: 15-22.

Mohamedsaid MS (1998c) Checklist of the Galerucinae from Taman Negara Gunung Gading, Sarawak (Coleoptera: Chrysomelidae). Serangga 3: 67-85.

Mohamedsaid MS (1998d) New species of *Taumacera* Thunberg from Borneo (Coleoptera: Chrysomelidae: Galerucinae). Serangga 3: 153-160.

Mohamedsaid MS (1998e) A new name for *Hoplosaenidea subcostata* (Laboissiere). Serangga 3: 169.

Mohamedsaid MS (1998f) Two new species of *Platyxantha* from Malaysia (Coleoptera: Chrysomelidae: Galerucinae). Serangga 3: 203-209.

Mohamedsaid MS (1998g) New species of leaf beetles from the MNS-Belum expedition (Coleoptera: Chrysomelidae: Galerucinae). Serangga 3: 227-238.

Mohamedsaid MS (1998h) Additional records of the Galerucinae from Sarawak, with descriptions of new species (Coleoptera: Chrysomelidae). Serangga 3: 247-268.

Mohamedsaid MS (1998i) *Paridea* (*Paraulaca*) *flavicornis* (Laboissiere), new combination. Serangga 3: 373-374.

Mohamedsaid MS (1999a) Two new species of *Medythia* Jacoby from Malaysia (Coleoptera: Chrysomelidae: Galerucinae). Genus 10: 415-420. http://www.biol.uni.wroc.pl/cassidae/medythia.pdf.

Mohamedsaid MS (1999b) *Sinoluperus beta*, n. sp., a second species for the genus (Coleoptera: Chrysomelidae: Galerucinae). Serangga 4: 17-20.

Mohamedsaid MS (1999c) The Galerucinae from Taman Kinabalu Sabah, Malaysia (Coleoptera: Chrysomelidae). Serangga 4: 87-145.

Mohamedsaid MS (1999d) New records of Galerucinae beetles from Peninsular Malaysia (Coleoptera: Chrysomelidae). Serangga 4: 221-238.

Mohamedsaid MS (1999e) *Azlania shehah*, new species, the fourth species for the genus. Raffles Bulletin of Zoology 47: 449-451. http://lkcnhm.nus.edu.sg/nus/pdf/PUBLICATION/Raffles%20Bulletin%20of%20Zoology/Past%20Volumes/RBZ%2047(2)/47rbz449-451.pdf

Mohamedsaid MS (1999f) Leaf beetles of the subfamily Galerucinae from Pulau Tioman, Peninsular Malaysia (Coleoptera: Chrysomelidae). Raffles Bulletin of Zoology Suppl. 6: 245-251. http://lkcnhm.nus.edu.sg/nus/pdf/PUBLICATION/Raffles%20Bulletin%20of%20Zoology/Supplements/Supplement%206/s6rbz245-251.pdf

Mohamedsaid MS (1999g) Notes on *Menippus* and *Issikia* from Sumatra and Java, Indonesia (Coleoptera: Chrysomelidae: Galerucinae). Zoologische Mededelingen 73: 187-188. http://www.repository.naturalis.nl/document/43554

Mohamedsaid MS (2000a) New species of Galerucinae from Borneo (Coleoptera: Chrysomelidae). Serangga 5: 281-308.

Mohamedsaid MS (2000b) The galerucine beetles from Tibow Sabah (Coleoptera: Chrysomelidae). Serangga 5: 309-314.

Mohamedsaid MS (2000c) List of Malaysian Chrysomelidae (Coleoptera) in the collection of UKM. Serangga 5: 343-360.

Mohamedsaid MS (2001a) *Cerophysa aseanica*, a new species of Chrysomelidae from Thailand (Coleoptera: Chrysomelidae: Galerucinae). Raffles Bulletin of Zoology 49: 251-252. http://lkcnhm.nus.edu.sg/nus/pdf/PUBLICATION/Raffles%20Bulletin%20of%20Zoology/Past%20Volumes/RBZ%2049(2)/49rbz251-252.pdf

Mohamedsaid MS (2001b) The genus *Xenoda* Baly from Malaysia (Coleoptera: Chrysomelidae: Galerucinae). Serangga 6: 17-36.

Mohamedsaid MS (2001c) The chrysomelid beetles of the subfamily Galerucinae from Bali, Indonesia (Coleoptera: Chrysomelidae). Serangga 6: 137-169.

Mohamedsaid MS (2001d) New species of *Parexosoma* Laboissiere from Malaysia (Coleoptera: Chrysomelidae: Galerucinae). Serangga 6: 171-180.

Mohamedsaid MS (2001e) New species of Galerucinae (Coleoptera: Chrysomelidae) from Malaysia. Serangga 6: 233-294.

Mohamedsaid MS (2002a) Two new species of leaf beetles from Singapore (Coleoptera: Chrysomelidae: Galerucinae). Malayan Nature Journal 56: 187-194.

Mohamedsaid MS (2002b) New species of Chrysomelidae from Sumatra and Sulawesi, Indonesia (Coleoptera: Chrysomelidae: Galerucinae). Serangga 7: 211-223.

Mohamedsaid MS (2003) New species of *Cerophysa* Chevrolat from Southeast Asia (Coleoptera: Chrysomelidae: Galerucinae). Genus 14: 381-391. http://www.biol.uni.wroc.pl/cassidae/Cerophysanew.pdf

Mohamedsaid MS (2004) Catalogue of the Malaysian Chrysomelidae (Insecta: Coleoptera). Pensoft, Sofia-Moscow, 239 pp.

Mohamedsaid MS (2005) *Monolepta wangkliana*, a new species of Galerucinae from Malaysia (Coleoptera: Chrysomelidae). Genus 16: 389-393. http://www.biol.uni.wroc.pl/cassidae/Monolepta%20wangkliana.pdf.

Mohamedsaid MS (2006) *Kumbornia tuberculata*, a new genus and species of Galerucinae from Borneo (Coleoptera: Chrysomelidae, Galerucinae). Entomologische Zeitschrift 116: 47-18.

Mohamedsaid MS (2007) *Haplosomoides annamitus* (Allard), a new record of genus and species from Cambodia (Coleoptera: Chrysomelidae: Galerucinae). Genus 18: 613-616. http://www.biol.uni.wroc.pl/cassidae/Mohamedsaid_Haplosomoides%20annamitus.pdf.

Mohamedsaid MS (2008) *Aplosonyx amorphophallus*, an interesting new species of Galerucinae from Timor, Indonesia (Coleoptera: Chrysomelidae). Zootaxa 1910: 53-58.

Mohamedsaid MS (2009a) A new species of *Hoplosaenidea* Laboissiere, 1933 from Malaysia (Coleoptera: Chrysomelidae: Galerucinae). Zootaxa 2300: 47-50.

Mohamedsaid MS (2009b) *Aulacophora sulaksonoi*, a new species of chrysomelid beetle from Sumba, Indonesia (Coleoptera: Chrysomelidae: Galerucinae). Genus 20: 335-339. http://www.biol.uni.wroc.pl/cassidae/Mohamedsaid_Aulacophora%20sulaksonoi_low.pdf

Mohamedsaid MS (2010) Three interesting new species of leaf beetles (Coleoptera: Chrysomelidae: Galerucinae) in BORNEENSIS collection at ITBC, Universiti Malaysia Sabah. Journal of Tropical Biology and Conservation 7: 59-64.

Mohamedsaid MS (2011) *Aplosonyx baliensis*, a new species of chrysomelid beetle from Bali, Indonesia (Coleoptera: Chrysomelidae: Galerucinae). Serangga 16: 1-6. http://ejournal.ukm.my/serangga/issue/viewIssue/553/76

Mohamedsaid MS, Constant J (2007) Chrysomelid beetles of the subfamily Galerucinae from Thailand and Cambodia in the collections of the Royal Belgian Institute of Natural Sciences (Coleoptera: Chrysomelidae). Bulletin de l´Institut Royal des Sciences Naturelles de Belgique, Entomologie 77: 163-177.

Mohamedsaid MS, Kimoto S (1993) An interesting new species of *Liroetiella* from Sabah, Malaysia (Coleoptera, Chrysomelidae, Galerucinae). Entomological Review of Japan 48: 45-46. http://coleoptera.sakura.ne.jp/ERJ/ERJ48(1)1993.pdf.

Mohamedsaid MS, Takizawa H (2007) *Kumbalia longicornis*, a new genus and species of Galerucinae (Coleoptera, Chrysomelidae). Serangga 12: 1-7. http://ejournal.ukm.my/serangga/issue/viewIssue/548/51

Moura LA de (1997) Nova espécie de *Aristobrotica* do Brasil central (Coleoptera, Chrysomelidae, Galerucinae). Iheringia, Ser. Zoologia 83: 195-198.

Moura LA de (1998a) Novo status de *Chlorolochmaea* (Coleoptera, Chrysomelidae, Galerucinae, Galerucini). Iheringia, Ser. Zoologia 84: 145-152.

Moura LA de (1998b) *Iucetima*, gênero novo de Galerucini da região neotropical. Iheringia, Ser. Zoologia 85: 75-88.

Moura LA de (1998c) Revisão do gênero *Neolochmaea* (Coleoptera, Chrysomelidae, Galerucinae, Galerucini). Iheringia, Ser. Zoologia 85: 169-188.

Moura LA de (2003) Nova espécie de *Zischkaita* Bechyné e notas taxonômicas em Galerucini (Coleoptera, Chrysomelidae, Galerucinae). Revista Brasileira de Zoologia 20: 643-645. doi: 10.1590/S0101-81752003000400014.

Moura LA de (2005) Novos táxons em Galerucini e redescrição de *Caraguata circumcincta* Clark (Coleoptera, Chrysomelidae, Galerucinae). Revista Brasileira de Zoologia 22: 1109-1115. doi: 10.1590/S0101-81752005000400042.

Moura LA de (2009) Morfologia comparada da genitália masculina de Galerucini (Coleoptera, Chrysomelidae, Galerucinae). Revista Brasileira de Entomologia 53: 15-22. doi: 10.1590/S0085-56262009000100005.

Moura LA de (2010) *Coronabrotica*, a new genus and species of Luperini, and a key to genera of Section Phyllecthrites (Coleoptera, Chrysomelidae, Galerucinae). Zootaxa 2675: 26-32. doi: 10.5281/zenodo.199200

Moura LA de (2011) A new species of *Aristobrotica* Bechyné and a checklist of the genus (Coleoptera, Chrysomelidae, Galerucinae). Revista Brasileira de Entomologia 55: 27-30. doi: 10.1590/S0085-56262011000100005.

Moura LA de (2016) New species, new combinations and synonymies of Neotropical Galerucini (Coleoptera, Chrysomelidae, Galerucinae). Zootaxa 4066: 63-70. doi: 10.11646/zootaxa.4066.1.4

Munroe DD, Smith RF (1980) A revision of the systematics of *Acalymma* sensu stricto Barber (Coleoptera: Chrysomelidae) from North America including Mexico. Memoirs of the Entomological Society of Canada 112: 1-92. doi: 10.4039/entm112112fv.

Nadein KS, Perkovsky EE, Moseyko AG (2016) New late eocene Chrysomelidae (Insecta: Coleoptera) from Baltic, Rovno and Danish ambers. Papers in Palaeontology 2: 117-137. doi: 10.1002/spp2.1034.

Nie RE, Zhou DK, Xue HJ, Yang XK (2013) Notes on black elytron species of *Pyrrhalta* Joannis and the description of a new species from China (Coleoptera, Chrysomelidae, Galerucinae). ZooKeys 289: 41-56. doi: 10.3897/zookeys.289.4266.

Ohno M (1963) Notes on *Aulacophora nigripennis* Motshulsky, with the description of a new subspecies (Coleoptera: Chrysomelidae: Galerucinae). Bulletin of the Biogeographical Society of Japan 22: 85-88.

Özdikmen H (2008) Substitute names for some preoccupied leaf beetles genus group names described by L. N. Medvedev (Coleoptera: Chrysomelidae). Munis Entomology & Zoology 3: 643-647. http://www.munisentzool.org/yayin/vol3/issue2/643-647.pdf

Pic M (1903) Deux captures intéressantes, diagnoses de divers Coléoptères. L’Échange 19: 145-147.

Prathapan KD (2016) Revision of the legume-feeding leaf beetle genus *Madurasia* Jacoby, including a new species description (Coleoptera, Chrysomelidae, Galerucinae, Galerucini). ZooKeys 597: 57-79. doi: 10.3897/zookeys.597.7520.

Radford WPK (1981) The Fabrician types of the Australian and New Zealand Coleoptera in the Banks collection at the British Museum (Natural History). Records of the South Australian Museum 18: 155-197.

Reid CAM (1998a) A new species of *Niasia* Jacoby (Coleoptera: Chrysomelidae: Galerucinae), with comments on the systematic position of the genus. Serangga 3: 191-202.

Reid CAM (1998b) The Chrysomeloidea of Taman Nasional Gede-Pangrango and environs, Jawa Barat, Indonesia. Serangga 3: 269-315.

Reid CAM (1999) Reappraisal of the genus *Taumacera* Thunberg with descriptions of two new species from South-East Asia (Coleoptera: Chrysomelidae: Galerucinae). Australian Journal of Entomology 38: 1-9. doi: 10.1046/j.1440-6055.1999.00074.x

Reid CAM (2001a) A new species of the genus *Taumacera* Thunberg, deusta species-group (Coleoptera: Chrysomelidae: Galerucinae) from Borneo. Raffles Bulletin of Zoology 49: 253-257. http://lkcnhm.nus.edu.sg/nus/pdf/PUBLICATION/Raffles%20Bulletin%20of%20Zoology/Past%20Volumes/RBZ%2049(2)/49rbz253-257.pdf

Reid CAM (2001b) *Gallerucella placida* Baly in Australia (Coleoptera: Chrysomelidae: Galerucinae). Australian Journal of Entomology 40: 331-334. doi: 10.1046/j.1440-6055.2001.00245.x.

Reid CAM (2003) Recognition of the genus *Hoplosaenidea* Laboissière in Australia, with a key to the Australian genera of Galerucini (Coleoptera: Chrysomelidae: Galerucinae). Australian Journal of Entomology 42: 40-45. doi: 10.1046/j.1440-6055.2003.00329.x

Reid CAM, Beatson M (2010) The genus *Atysa* Baly in Australia (Coleoptera: Chrysomelidae: Galerucinae). Australian Entomologist 36: 189-200.

Reid CAM, Nally SC (2008) Revision of the genus *Menippus* Clark in Australia (Coleoptera: Chrysomelidae: Galerucinae). Australian Journal of Entomology 47: 87-101. doi: 10.1111/j.1440-6055.2008.00634.x

Riley EG (1979) A new species of *Phyllobrotica* Chevrolat (Coleoptera: Chrysomelidae) from the prairies of southwestern Missouri. Coleopterists Bulletin 33: 331-335.

Riley EG, Clark SM, Gilbert AJ (2001) New records, nomenclatural changes, and taxonomic notes for select North American leaf beetles (Coleoptera: Chrysomelidae). Insecta Mundi 15: 1-17.

Riley EG, Clark SM, Seeno TN (2003) Catalog of leaf beetles of America north of Mexico. The Coleopterists Society, Sacramento, 290 pp.

Rizki A, Hazmi IR, Ruslan MY, Idris AB (2014) Redescription of *Paleosepharia azlani* (Mohamedsaid, 1998) (Coleoptera: Chrysomelidae: Galerucinae). Serangga 19: 31-38. doi: http://ejournal.ukm.my/serangga/article/view/11705/3756

Rizki A, Hazmi IR, Wagner T, Idris AB (2016) Redescription of *Paleosepharia trunctata* Laboissiere, 1936, type species of the genus *Paleosepharia* Laboissiere, 1936 (Coleoptera: Chrysomelidae: Galerucinae). Coleopterists Bulletin 70: 395-398. doi: 10.1649/0010-065X-70.2.395.

Rizvi SG, Kamaluddin S (2011) *Oides neobengalensis*: a new species of the genus *Oides* Weber (Coleoptera: Chrysomelidae: Galerucinae) from Pakistan. Pakistan Journal of Entomology (Karachi) 26: 57-60.

Rodrigues JMS, Mermudes JRM (2015) Comparative morphology of the type-species of *Isotes* and *Synbrotica* (Coleoptera, Chrysomelidae, Galerucinae), with a new synonymy of species. Iheringia Serie Zoologia 105: 439-452. doi: 10.1590/1678-476620151054439452

Romantsov PV (2004) A description of a new species of the leaf-beetle genus *Luperus* Geoffr (Coleoptera, Chrysomelidae) from eastern Kazakhstan and notes on some related species. Entomologicheskoe Obozrenie 83: 622-624.

Ruppel RF (1978) A new species of *Cerotoma* from Bolivia (Coleoptera, Chrysomelidae). Journal of the Kansas Entomological Society 51: 28-30.

Samoderzhenkov EV (1988) Zhuki-listoedy triby Galerucini (Chrysomelidae, Galerucinae) fauny Vietnama. In: Medvedev LN, Striganova BR (Eds) Fauna i ekologiya nasekomykh Vietnama. Moskva, Nauka, 70-95.

Samoderzhenkov EV (1992) Obzor zhukov-listoedov (Coleoptera, Chrysomelidae, Galerucinae) triby Luperini iz Vietnama. In: Medvedev LN (Ed) Sistematika i ekologiya nasekomykh Vietnama. Moskva, Nauka, 103-127.

Scherer G (1969) Die Alticinae des indischen Subkontinentes. Pacific Insects Monograph 22: 1-251. http://hbs.bishopmuseum.org/pim/pdf/pim22.pdf

Scherz X, Wagner T (2007) Revision of *Afrocandezea* Wagner & Scherz, 2002 from tropical Africa (Coleoptera: Chrysomelidae: Galerucinae). Entomologische Zeitschrift 117: 161-183.

Schlechtendal DHR von (1894) Beiträge zur kenntnis fossiler insekten aus dem Braunkohlengebirge von Rott am Siebengebierge. Abhandlungen der Naturforschenden Gesellschaft zu Halle 20: 197-228 + pls. XII-XIV.

Schlich C, Wagner T (2010) Species of *Monolepta* Chevrolat, 1836 from the Arabian Peninsula (Coleoptera, Chrysomelidae, Galerucinae). Entomologica Basiliensia et Collectionis Frey 32: 307-322.

Schmitz J, Wagner T (2001) *Afromegalepta* gen. nov. from tropical Africa (Coleoptera: Chrysomelidae, Galerucinae). Entomologische Zeitschrift 119: 283-286.

Selman BJ (1963) Coléoptères Chrysomélides récoltés par M. J. Mateu dans l'Ennedi et au Tchad. Bulletin de l’Institut Français d’Afrique Noire 25: 1148-1162.

Shute SL (1983) Key to the genera of galerucine beetles of New Guinea, with a review of *Sastra* and related new taxa (Chrysomelidae). Bulletin of the British Museum (Natural History), Entomology Series 46: 205-266.

Silfverberg H (1969) What is *Galleruca tumida* Gyllenhal (Coleoptera, Chrysomelidae)? Contribution to the study of the Galerucinae. 1. Notulae Entomologicae 49: 268.

Silfverberg H (1971) A revision of the genus *Cannonia* Hincks (Coleoptera, Chrysomelidae). Contribution to the study of the Galerucinae 2. Notulae Entomologicae 51: 59-70.

Silfverberg H (1972) *Aulacophorina* from North-East Africa (Coleoptera, Chrysomelidae). Contribution to the study of the Galerucinae 3. Notulae Entomologicae 52: 41-46.

Silfverberg H (1973a) A new Mahutia-species from East Africa (Col. Chrysomelidae). Contribution to the study of Galerucinae 5. Entomologica Scandinavica 4: 169-170. doi: 10.1163/1876312X74X00029

Silfverberg H (1973b) A revision of the genus *Prosmidia* Weise (Coleoptera, Chrysomelidae). Acta Zoologica Fennica 139: 1-54.

Silfverberg H (1974) The West Palaearctic species of *Galerucella* Crotch and related genera (Coleoptera, Chrysomelidae). Contribution to the study of Galerucinae 6. Notulae Entomologicae 54: 1-11.

Silfverberg H (1975a) The genera *Spilonotella* Cockerell, *Austrotella* gen. n. and *Shungwayana* gen. n (Coleoptera: Chrysomelidae). Contribution to the study of Galerucinae 9. Entomologica Scandinavica 6: 275-282. doi: 10.1163/187631275X00118.

Silfverberg H (1975b) A revision of the genus *Laetiacantha* Laboissière (Coleoptera, Chrysomelidae). Contribution to the study of Galerucinae 7. Notulae Entomologicae 55: 1-12.

Silfverberg H (1975c) A revision of the genus *Neolaetana* Laboissière (Coleoptera, Chrysomelidae). Contribution to the study of Galerucinae 8. Notulae Entomologicae 55: 33-44.

Silfverberg H (1978a) *Taenala*, a new genus of African Galerucinae (Coleoptera: Chrysomelidae). Contribution to the study of Galerucinae 11. Entomologica Scandinavica 9: 31-34. doi: 10.1163/187631278X00188.

Silfverberg H (1978b) *Oorlogia nigriceps* n. gen., n. sp. from Namibia (Coleoptera: Chrysomelidae). Contribution to the study of Galerucinae 12. Entomologica Scandinavica 9: 78-79. doi: 10.1163/187631278X00269.

Silfverberg H (1978c) The identity of *Aulacophora pannonica* Csiki (Coleoptera: Chrysomelidae). Contribution to the study of Galerucinae 13. Folia Entomologica Hungarica 31: 219-220. http://publication.nhmus.hu/pdf/folentom/FoliaEntHung_1978_Vol_31_2_205.pdf

Silfverberg H (1978d) The coleopteran genera of Müller 1764. Notulae Entomologicae 58: 117-119.

Silfverberg H (1979) *Anaspis* Müller, 1764; *Luperus* Müller, 1764; *Lampyris* Müller, 1764; and *Clerus* Müller, 1764 (Insecta: Coleoptera): proposed designation of type species. Z.N. (S.) 2240. Bulletin of Zoological Nomenclature 36: 161-166.

Silfverberg H (1980) *Mahutia rougemonti* n. sp. from Ethiopia (Coleoptera: Chrysomelidae). Contribution to the study of Galerucinae 14. Entomologica Scandinavica 11: 37-38. doi: 10.1163/187631280X00310.

Silfverberg H (1982a) Dismemberment of the genus *Laetana* Baly (Coleoptera: Chrysomelidae). Annales Entomologici Fennici 48: 17-30.

Silfverberg H (1982b) A generic revision of the group *Prosmidiites* (Coleoptera: Chrysomelidae). Entomologica Scandinavica 13: 173-182. doi: 10.1163/187631282X00075.

Silfverberg H (1990) Records of Galerucinae (Coleoptera, Chrysomelidae) from the Indian subcontinent. Part 1. Entomologica Fennica 1: 201-207.

Skomorokhov MO (2011) To the knowledge of Chrysomelidae (Coleoptera) from the islands of southern Vietnam. Russian Entomological Journal 20: 197-199. http://kmkjournals.com/upload/PDF/REJ/20/ent20_2_197_199_Skomorokhov.pdf

Sprecher-Uebersax E, Zoia S (2002) *Pyrrhalta medvedevi* sp. nov., a new species from the Nepal Himalayas (Coleoptera, Chrysomelidae, Galerucinae). Mitteilungen der Schweizerischen Entomologischen Gesellschaft 75: 161-167.

Stapel H, Wagner T (2000) Revision of *Monoleptocrania* Laboissière, 1940 (Coleoptera: Chrysomelidae: Galerucinae). Mitteilungen des Internationalen Entomologischen Vereins E.V. Frankfurt A.M. 25: 137-145.

Stapel H, Wagner T (2001) *Afrotizea* gen. nov. from tropical Africa (Coleoptera: Chrysomelidae: Galerucinae). Beiträge zur Entomologie 51: 365-373.

Steiner I, Wagner T (2005) *Afronaumannia* gen. nov., a new monophyletic group of leaf beetles from Africa (Coleoptera: Chrysomelidae, Galerucinae). Entomologische Zeitschrift 115: 15-24.

Takizawa H (1978) Notes on Taiwanese Chrysomelidae. I. Kontyû 46: 123-134.

Takizawa H (1985a) Notes on chrysomelid-beetles (Coleoptera, Chrysomelidae) of India and its neighbouring areas, part 1. Kontyû 53: 565-575.

Takizawa H (1985b) Notes on chrysomelid-beetles of India and its neighboring areas, part II (Coleoptera, Chrysomelidae). Entomological Review of Japan 40: 95-114. http://coleoptera.sakura.ne.jp/ERJ/ERJ40(2)1985.pdf.

Takizawa H (1985c) Notes on Korean Chrysomelidae, part 2. Nature and Life 15: 1-18.

Takizawa H (1986a) Notes on chrysomelid-beetles of India and its neighboring areas, part 3 (Coleoptera, Chrysomelidae). Entomological Review of Japan 41: 35-47. http://coleoptera.sakura.ne.jp/ERJ/ERJ41(1)1986.pdf.

Takizawa H (1986b) Chrysomelidae collected by the Japan-India Cooperative Survey in India, 1978. Part 3. Entomological Review of Japan 41: 93-105. http://coleoptera.sakura.ne.jp/ERJ/ERJ41(2)1986.pdf.

Takizawa H (1987) Notes on chrysomelid beetles (Coleoptera, Chrysomelidae) of India and its neighbouring areas, part 6. Kontyû 55: 521-529.

Takizawa H (1988a) Chrysomelid beetles of Nepal, collected by the Hokkaido University scientific expeditions to Nepal Himalaya Part IV (Coleoptera: Chrysomelidae). Entomological Review of Japan 43: 1-16. http://coleoptera.sakura.ne.jp/ERJ/ERJ43(1)1988.pdf.

Takizawa H (1988b) Notes on chrysomelid beetles (Coleoptera, Chrysomelidae) of India and its neighboring areas. Part 7. Kontyû 56: 534-552.

Takizawa H (1990) Chrysomelid beetles of Nepal, northeastern India and western Sikkim collected by the Himalaya Expeditions of the National Science Museum, Tokyo (Part 2). Japanese Journal of Entomology 58: 275-291.

Takizawa H (2011) Description of a new species of Borneola Mohamedsaid from Borneo with notes on their larvae (Coleoptera: Chrysomelidae: Galerucinae). Serangga 16 (2): 1-18. http://ejournal.ukm.my/serangga/issue/viewIssue/552/70

Takizawa H, Basu CR (1987) Notes on chrysomelid-beetles (Coleoptera, Chrysomelidae) of India and its neighbouring areas. Part 4. Kontyû 55: 266-283.

Takizawa H, Kimoto S (1990) Notes on chrysomelid beetles of S. India preserved in the collection of the Staatliches Museum of Munich (Coleoptera: Chrysomelidae). Entomological Review of Japan 45: 7-10. http://coleoptera.sakura.ne.jp/ERJ/ERJ45(1)1990.pdf.

Takizawa H, Mohamedsaid MS (2015) Descriptions of four new species of the *Hoplosaenidea takizawai* group from the Greater Sunda Islands area (Coleoptera: Chrysomelidae: Galerucinae). Journal of Tropical Biology and Conservation 12: 113-125. http://www.ums.edu.my/ibtpv2/images/publication/JTBC/JTBC-VOL-12/09_JTBC12_023_11.pdf

Tomov V (1975) Eine neue *Luperus*-Art aus Südjugoslawien (Coleoptera, Chrysomelidae, Galerucinae). Entomologische Arbeiten aus dem Museum G. Frey 26: 188-190. http://www.zobodat.at/pdf/Entomologische-Arbeiten-Museum-Frey_26_0188-0190.pdf

Tracy JL, Robbins TO (2009) Taxonomic revision and biogeography of the Tamarix-feeding *Diorhabda elongata* (Brullé, 1832) species group (Coleoptera: Chrysomelidae: Galerucinae: Galerucini) and analysis of their potential in biological control of Tamarisk. Zootaxa 2101: 1-152. http://www.mapress.com/zootaxa/2009/f/zt02101p152.pdf

Tubbs PK (1991) Case 1707. *Diabrotica undecimpunctata* Mannerheim, 1843 (Insecta, Coleoptera): proposed conservation of the specific name, and of the subspecific name howardi Barber, 1947. Bulletin of Zoological Nomenclature 48: 219-221.

Vachon A (1976) Galerucinae de la Nouvelle-Guinée (1^re^ note). Description d´un genre et de deux espèces nouveaux (Col. Chrysomelidae Oidini). Bulletin de la Société Entomologique de France 81: 88-96.

Vachon A (1977) Galerucinae de l´Asie du Sud-Est et de la Nouvelle-Guinée (2^e^ note) (Col. Chrysomelidae). Oidini - Nouvelles synonymies. Bulletin de la Société Entomologique de France 82: 12-14.

Vachon A (1980a) Les Adorium de Fabricius - Systématique et synonymies (Col. Chrysomelidae). Bulletin de la Société Entomologique de France 85: 14-19.

Vachon A (1980b) Galerucinae Oidini de la Nouvelle-Guinée (3^e^ note) (Col. Chrysomelidae). Etudes, synonymies et descriptions d´espèces nouvelles. Bulletin de la Société Entomologique de France 85: 195-207.

Vazirani TG (1970) A new species of *Merista* Chapuis (1895) (Insecta: Coleoptera: Chrysomelidae) from India. Records of the Zoological Survey of India 64: 111-112.

Vela JM, Bastazo G (1990) Morphological and genital patterns distinguishing *Luperus* Geoffroy, 1762 and *Calomicrus* Dillwyn, 1824 (Col., Chrysomelidae). Eos 66: 187-200.

Vela JM, García Beccera R (1996) *Calomicrus doramasensis* n. sp., a new leaf beetle from the Canary Islands (Coleoptera, Chrysomelidae, Galerucinae). Vieraea 25: 147-152. http://www.azoresbioportal.angra.uac.pt/files/publicacoes_Vieraea_25_1996_12.pdf.

Virkki N (1979) Brief notes on the cytology of Neotropical Coleoptera. III. “*Luperodes antillarum* Blake” = *Lysathia ludoviciana* (Fall). Journal of Agriculture of the University of Puerto Rico 63: 100-101.

Wagner T (2000a) New *Monolepta* species (Coleoptera: Chrysomelidae: Galerucinae) from Eastern Africa. Entomologische Zeitschrift 110: 34-40.

Wagner T (2000b) Revision of Afrotropical *Monolepta* Chevrolat, 1837 (Coleoptera: Chrysomelidae, Galerucinae). Part I: species with red and black coloured elytra, pronotum and head, with description of new species. Entomologische Zeitschrift 110: 226-237.

Wagner T (2001a) Revision of Afrotropical *Monolepta* Chevrolat, 1837 (Coleoptera: Chrysomelidae, Galerucinae). Part II: Species with red elytra, pronotum and head, with descriptions of new species. Bonner Zoologische Beiträge 50: 49-65. http://www.zoologicalbulletin.de/BzB_Volumes/Volume_50_1_2/049_065_BZB50_1_2_Wagner_Thomas.PDF

Wagner T (2001b) New *Monolepta* species (Coleoptera, Chrysomelidae, Galerucinae) from Central and Southern Africa. Entomologische Blätter 96 (2000): 199-209.

Wagner T (2002) Revision of Afrotropical *Monolepta* species. Part III: species with red elytra and yellow prothorax, including descriptions of new species. Mitteilungen aus dem Museum für Naturkunde in Berlin 49: 27-45. doi: mmnd.20020490104.

Wagner T (2003) Revision of afrotropical *Monolepta* Chevrolat, 1837 (Coleoptera, Chrysomelidae, Galerucinae). - Part IV: Species with red head and thorax and black elytra or black elytra with red apex, with description of new species. Annales du Musée Royal de l'Afrique Centrale (Zoologie) 291: 37-89.

Wagner T (2005) Revision of the vincta Species-group of *Monolepta* Chevrolat, 1837 from Africa, Arabia and the Near East (Coleoptera: Chrysomelidae, Galerucinae). Bonner Zoologische Beiträge 53(2004): 255-282. http://www.zoologicalbulletin.de/BzB_Volumes/Volume_53_1_2/255_282_BZB53_1_2_Wagner_Thomas.PDF

Wagner T (2007a) Revision of *Afrocrania* (Coleoptera: Chrysomelidae: Galerucinae) Part II: species in which the males lack head cavities or extended elytral extrusions. European Journal of Entomology 104: 801-814. doi: 10.14411/eje.2007.101.

Wagner T (2007b) Revision of Afrotropical *Monolepta* Chevrolat, 1837 - Part VI: species with reddish or black cross-elytral pattern (Coleoptera: Chrysomelidae, Galerucinae). Journal of Afrotropical Zoology 3: 83-152.

Wagner T (2007c) *Monolepta* Chevrolat, 1837, the most speciose galerucine taxon: redescription of the type species Monolepta bioculata (Fabricius, 1781) and key to related genera from (Chrysomelidae, Coleoptera). Journal of Natural History 41: 81-100. doi: 10.1080/00222930601127384.

Wagner T (2011) Description of *Monoleptoides* gen. nov. from the Afrotropical Region, including the revision of nine species (Coleoptera: Chrysomelidae: Galerucinae). Bonn Zoological Bulletin 60: 169-199. http://www.zoologicalbulletin.de/BzB_Volumes/Volume_60_2/169-199_BzB%2060(2)_Wagner,%20Thomas.pdf.

Wagner T (2016) The *Monolepta* species of Namibia (Coleoptera, Chrysomelidae, Galerucinae). Entomologische Blätter und Coleoptera 112: 407-442.

Wagner T, Bieneck S (2012) Galerucine type material described by Victor Motschulsky in 1858 and 1866 from the Zoological Museum Moscow (Coleoptera: Chrysomelidae, Galerucinae). Entomologische Zeitschrift 122: 205-216.

Wagner T, Freund W (2003) Revision of *Barombiella violacea* (Jacoby, 1894). Entomologische Zeitschrift 113: 258-262.

Wagner T, Kurtscheid A (2005) Revision of *Candezea* Chapuis, 1879 (Coleoptera, Chrysomelidae, Galerucinae) from continental Africa. Journal of Natural History 39: 2591-2641. doi: 10.1080/00222930500102611.

Wagner T, Scherz X (2002) Afrocandezea gen. nov. from tropical Africa (Coleoptera: Chrysomelidae: Galerucinae). Entomologische Zeitschrift 112: 357-362.

Wang HJ, Li WZ, Yang XK (2000) Study on the genus *Sphenoraia* Clark (Coleoptera: Chrysomelidae: Galerucinae) from China. In: Zhang Y (Ed) Systematic and faunistic research on Chinese insects. Proceedings of the 5th National Congress of Insect Taxonomy. China Agriculture Press, place of publication not given, 117-121.

Wang JS, Yang XK (Eds) (1998) Fauna of Chrysomelidae of Wuyishan Nature Reserve in China. China Forestry Publishing House, 213 pp., 8 pls.

Warchałowski A (1991) Kurze Übersicht der oberseits hellen *Calomicrus* Steph.-Arten (Coleoptera: Chrysomelidae: Galerucinae). Genus 2: 41-55.

Warchałowski A (1998) Die westpaläarktischen Arten der Gattung Phyllobrotica Chevrolat, 1937 (Coleoptera: Chrysomelidae: Galerucinae). Annales Zoologici 48: 91-98.

Warchałowski A (2001) *Geinella trapezicollis* sp. nov., a new species from Tibet (Coleoptera: Chrysomelidae: Galerucinae). Annales Zoologici 51: 185-187.

Warchałowski A (2004) Nomenclatorial notes in Chrysomelidae (Coleoptera: Chrysomeloidea). Genus 15: 113-114. http://www.biol.uni.wroc.pl/cassidae/Nomenclature%20Chrysomelidae.pdf.

Warchałowski A (2008) Complementary description of *Liroetis clermonti* Laboissière, 1929 (Coleoptera: Chrysomelidae: Galerucinae: Luperini). Genus 19: 695-697. http://www.biol.uni.wroc.pl/cassidae/Warchalowski_Liroetis%20clermonti.pdf.

Weise J (1913) Chrysomeliden und Coccinelliden. Nova Guinea 9: 423-446.

Weise J (1926) Uber bekannte und neue Chrysomeliden und Coccinelliden aus dem Reichsmuseum zu Stockholm. Arkiv för Zoologi 18A (34): 1-34.

White RE (1979) A Neotropical leaf beetle established in the United States (Chrysomelidae). Annals of the Entomological Society of America 72: 269-270. doi: 10.1093/aesa/72.2.269.

Wu SY, Yang XK, Li WZ (1998) A new species of Galerucinae (Coleoptera: Chrysomelidae) from Wuyi Mountains. Entomotaxonomia 20: 182-184.

Xue HJ, Yang XK (2010) Species catalogue of *Pyrrhalta* Joannis (Coleoptera: Chrysomelidae: Galerucinae) of the World. Entomotaxonomia 32(Suppl.): 119-136.

Yang XK (1991a) Study on the genus *Paridea* Baly from China (Chrysomelidae: Galerucinae). Sinozoologia 8: 267-295.

Yang XK (1991b) Notes on Chinese species of the genus *Sermyloides* Jacoby (Coleoptera, Chrysomelidae: Galerucinae). Sinozoologia 8: 297-305.

Yang XK (1991c) Study on the genus *Pseudocophora* Jacoby from China (Coleoptera, Chrysomelidae: Galerucinae). Sinozoologia 8: 307-312.

Yang XK (1991d) Review on the genus *Cneoranidea* (Chrysomelidae: Galerucinae). Scientific Treatise on Systematic and Evolutionary Zoology 1: 199-206.

Yang XK (1991e) *Sermyloidea pilifera*, a new species from China (Coleoptera: Chrysomelidae, Galerucinae). Acta Entomologica Sinica 34: 349-351.

Yang XK (1992a) The Chinese species of the genus *Strobiderus* (Coleoptera: Chrysomelidae, Galerucinae). Sinozoologia 9: 187-189.

Yang XK (1992b) The Chinese known species of the genus *Macrima* and description of one new species (Coleoptera: Chrysomelidae, Galerucinae). Zoological Research 13: 257-261.

Yang XK (1992c) Galerucid beetles of Mount Mogan and descriptions of two new species (Coleoptera: Chrysomelidae, Galerucinae). Journal of Zhejiang Forestry College 9: 409-413.

Yang XK (1992d) Coleoptera: Chrysomelidae–Galerucinae. In: Huang F (Ed) Insects of Wuling Mountains area, southwestern China. Science Press, Beijing, 331-346.

Yang XK (1992e) Chrysomelidae Galerucinae. In: Peng J, Liu Y (Eds) Iconography of forest insects in Hunan China. Academia Sinica & Hunan Forestry Institute, Hunan, 552-589.

Yang XK (1993a) A new species of the genus *Sermyloides* (Coleoptera: Chrysomelidae: Galerucinae). Acta Zootaxonomica Sinica 18: 456-458.

Yang XK (1993b) Notes on the genus *Fleutiauxia* Laboissiere (Coleoptera: Chrysomelidae: Galerucinae). Entomotaxonomia 15: 219-227.

Yang XK (1993c) Three new species of the genus *Paridea* from China (Coleoptera: Chrysomelidae: Galerucinae). Acta Zootaxonomica Sinica 18: 196-200.

Yang XK (1993d) Study on the genus *Laphris* and its phylogenetic relationship with related genera (Coleoptera: Chrysomelidae: Galerucinae). Acta Zootaxonomica Sinica 18: 362-369.

Yang XK (1994a) Study on the genus *Gallerucida* (I). Species of which the elytra with black punctures (Coleoptera: Chrysomelidae: Galerucinae). Acta Zootaxonomica Sinica 19: 202-205.

Yang XK (1994b) Study on the genus *Gallerucida* (II) Descriptions of five new species (Coleoptera: Chrysomelidae: Gallerucinae). Acta Zootaxonomica Sinica 19: 343-350.

Yang XK (1995a) Studies on the subfamily Galerucinae I. Complementary description of the genus *Aplosonyx* and the descriptions of two new species (Coleoptera: Chrysomelidae). Acta Zootaxonomica Sinica 20: 90-94.

Yang XK (1995b) Coleoptera: Chrysomelidae - Galerucinae. In: Wu H (Ed) Insects of Baishanzu Mountain, Eastern China. China Forestry Publishing house, Beijing, 259-263.

Yang XK (1996) Revision of the genus *Arthrotidea* Chen (Coleoptera: Chrysomelidae: Galerucinae). Bulletin of the National Museum of Natural Science (Taichung) 8: 29-34.

Yang XK (2002) Chrysomelidae: Galerucinae. In: Huang PK (Ed) Fauna of Insects of Fujian Province of China. Vol. 6. Fujian Science & Technology Press, Fuzhou, 621-663.

Yang XK, Gan YL (1993) Discussion on the status of three allied species of the genus *Monolepta* (Chrysomelidae: Galerucinae). Scientific Treatise on Systematic and Evolutionary Zoology 2: 99-105.

Yang XK, Ge SQ, Li WZ (2001) Revision of the genus *Agetocera* Hope (Coleoptera: Chrysomelidae: Galerucinae). Oriental Insects 35: 105-154. doi: 10.1080/00305316.2001.10417293.

Yang XK, Li WZ (1998) Coleoptera: Chrysomelidae: Galerucinae. In: Wu H (Ed) Insects of Longwangshan Nature Reserve. China Forestry Publishing House, Beijing, 128-135.

Yang XK, Li WZ (2004) Coleoptera: Chrysomelidae-Galerucinae. In: Yang XK (Ed) Insects of the Great Yarlung Zangbo Canyon of Xizang. Beijing, 73-77.

Yang XK, Li WZ, Yang J, Yu Y, Cao Z (1995) *Sastracella* Jacoby, the first record from China, and description of one new species (Coleoptera: Chrysomelidae). Sinozoologia 12: 210-214.

Yang XK, Li WZ, Yao J (1997) The galerucine beetles of Xishuangbanna, Yunnan province (Coleoptera: Chrysomelidae). Acta Zootaxonomica Sinica 22: 384-391.

Yang XK, Li WZ, Zhang BQ, Xiang ZQ (1997) Coleoptera: Chrysomelidae: Galerucinae. In: Yang XK (Ed) Insects of the Three Gorge reservoir area of Yangtze River. Part 1. Chongqing Publishing House, Chongqing, 863-904.

Yang XK, Wang J, Wu Y (1998) Three new species of Galerucinae (Coleoptera: Chrysomelidae) from Wuyi Mountain. Entomotaxonomia 20: 261-264.

Yu PY, Wang SY, Yang XK (1996) Coleoptera: Chrysomeloidea (II). Economic Insect Fauna of China. Fasc. 54. Science Press, Beijing, xvi + 324 pp.

Zhang LJ, Beenen R, Yang XK (2009) *Clitenella yunnana* (Yang and Li, 1997), new combination (Coleoptera: Chrysomelidae: Galerucinae). Proceedings of the Entomological Society of Washington 111: 274-275. doi: 10.4289/0013-8797-111.1.274.

Zhang LJ, Li WZ, Yang XK (2008a) A new species of *Siemssenius* Weise (Coleoptera: Chrysomelidae: Galerucinae) from China, and a key to the known species. Proceedings of the Entomological Society of Washington 110: 126-129. doi: 10.4289/0013-8797-110.1.126.

Zhang LJ, Li WZ, Yang XK (2008b) Taxonomic changes in the genus *Paraplotes* Laboissière, 1933 (Coleoptera: Chrysomelidae: Galerucinae). Pan-Pacific Entomologist 84: 17-21.

Zhang LJ, Li WZ, Yang XK (2008c) Two new species of the genus *Liroetis* Weise, 1889, from China (Coleoptera: Chrysomelidae: Galerucinae). Pan-Pacific Entomologist 84: 22-25.

Zhang LJ, Li WZ, Zhang Y, Yang XK (2008) A new species of genus *Aplosonyx* Chevrolat (Coleoptera: Chrysomelidae: Galerucinae) from China, with a key to the Chinese known species. Zootaxa 1898: 63-68. doi: 10.5281/zenodo.184475.

Zhang LJ, Yang XK (2002) Study on the genus *Meristata* Strand (Coleoptera: Chrysomelidae: Galerucinae) of China. Entomotaxonomia 24: 245-253.

Zhang LJ, Yang XK (2004) A review of the genus *Paragetocera* Laboissiere (Coleoptera: Chrysomelidae: Galerucinae). Oriental Insects 38: 289-302. doi: 10.1080/00305316.2004.10417395.

Zhang LJ, Yang XK (2005a) A new species of the genus *Agetocera* Hope (Coleoptera: Chrysomelidae: Galerucinae) from China. Proceedings of the Entomological Society of Washington 107: 119-122.

Zhang LJ, Yang XK (2005b) A new species of the genus *Cneoranidea* Chen, 1942 from China with a key to the known species (Coleoptera: Chrysomelidae: Galerucinae). Pan-Pacific Entomologist 81: 47-53.

Zhang LJ, Yang XK, Cui JZ, Li WZ (2006) A key to the genus *Mimastra* Baly (Coleoptera: Chrysomelidae: Galerucinae) from China, with the description of a new species. Entomological News 117: 203-210. doi: 10.3157/0013-872X(2006)117[203:AKTTGM]2.0.CO;2

Zulfadli M, Izfa RH, Alia RA (2015) Morphology and genitalia characters of *Nadrana* Baly, 1865 (Coleoptera: Chrysomelidae: Galerucinae) from Sundaland. Malaysian Applied Biology 44: 97-108. http://mabjournal.com/images/44_3_October_2015/44_1_18.pdf
